# Global, regional, and national metabolic risk factors associated with multiple myeloma, 1990-2021: analysis via the global burden of disease study 2021

**DOI:** 10.3389/fonc.2025.1659527

**Published:** 2025-09-09

**Authors:** Guozhi Zhang

**Affiliations:** Clinical Medical College, Shaanxi University of Chinese Medicine, Xianyang, China

**Keywords:** multiple myeloma, burden, epidemiology, mortality, global

## Abstract

**Background and objectives:**

This study aimed to examine the global disease burden of multiple myeloma (MM) linked to metabolic factors. Using data from GHDx spanning 1990–2021, MM cases were identified via ICD-10 codes (C90.0). Key metrics, including mortality rate and disability-adjusted life years (DALYs) rate, were analyzed; age-standardized rates (ASMR, ASDR) and estimated annual percentage change (EAPC) were computed using R to compare disparities across regions, genders, and age groups.

**Methods and data sources:**

Thirty-one years of data from GHDx were utilized to capture temporal trends. MM cases were coded according to ICD-10 (C90.0), and age-standardized rates were applied to reduce demographic biases. Temporal changes were assessed via EAPC, while differences across regions, genders, and age groups were analyzed through comparisons of ASMR and ASDR.

**Key findings:**

From 1990 to 2021, global deaths from metabolic-related MM tripled, with DALYs increasing by 2.8 times. Mortality rates, ASMR, and ASDR showed significant upward trends—slowing between 2000 and 2010 before rebounding. Males exhibited higher ASDR and ASMR, attributed to lifestyle factors and estrogen-mediated protection in females. Middle-SDI countries saw sharp increases in ASDR (driven by population aging and limited healthcare access), whereas High-SDI countries exhibited slower growth (due to advanced treatment options). Most regions recorded rising ASDR, except in high-income Asia Pacific and North America (attributed to novel therapeutic agents). The United States, China, and Germany recorded the highest number of deaths (linked to population size and aging), while countries such as the Bahamas had elevated rates. Underdeveloped regions grappled with underdiagnosis problems. Age-specific analysis showed a rising burden among those aged 70–74 years and older, with elderly males being the most affected group in 2021.

**Conclusions:**

The global burden of metabolic-related MM is shaped by demographic factors, economic conditions, lifestyle, and healthcare provision. High-risk groups (elderly males, Middle-SDI countries, and regions with poor metabolic health) necessitate tailored, region-specific prevention strategies.

## 1 Introduction

Clinically, multiple myeloma (MM) ranks as the second most common hematological malignancy, arising from the abnormal lineage of plasma cells (1). It originates from monoclonal gammopathy of undetermined significance and can progress to plasma cell leukemia and extramedullary myeloma (2). MM has a median age of onset of 69 years, with the patient population predominantly comprising older adults. These elderly patients frequently have comorbid metabolic syndrome (MetS)-related underlying diseases (3), MetS is primarily characterized by central obesity, insulin resistance, diabetes, dyslipidemia, and hypertension. A study by Gavriatopoulou M et al. (4) demonstrated that the prevalence of MetS and its individual components is significantly higher in MM patients than in the general population. MetS is a pathological state characterized by the clustering of multiple metabolic abnormalities, primarily including abdominal obesity, hyperglycemia, hypertension, and dyslipidemia (such as hypertriglyceridemia, low high-density lipoprotein cholesterol). These factors are interrelated and interact synergistically, significantly increasing the risk of chronic diseases such as cardiovascular disease and type 2 diabetes (5). MetS is a common metabolic disorder syndrome (5). MetS exerts a profound influence on MM. Current literature lacks analyses of the global burden of MM attributable to MetS. Therefore, this study aims to investigate the disease burden of multiple myeloma in the general population across global, regional, and national levels, integrating metabolic factors, to characterize the global distribution of MM linked to metabolic factors, identify high-risk populations and regions (6), assess their associations with metabolic factors, inform public health policy development, and provide a foundation for optimizing prevention and control strategies.

## 2 Methods

### 2.1 Study population

This study focuses on patients of all ages with multiple myeloma attributable to metabolic risk factors including High fasting plasma glucose (≥7.0mmol/L), High low density lipoprotein (LDL) cholesterol (≥4.1mmol/L), High systolic blood pressure (≥140mmHg), High body-mass index (≥30.0kg/m^2^), Low bone mineral density (T-score<-1.0) and Kidney dysfunction (Glomerular filtration rate (GFR), GFR<60 mL/min/1.73m² persisting for more than 3 months).

### 2.2 Data collection

The multiple myeloma diagnosis defined in this study corresponds to the ICD-10 codes “C90.0”. The data was obtained from the Global Health data Exchange(Global Health data Exchange, GHDx) (http://ghdx.healthdata.org/). The search parameters were “multiple myeloma” for cause; “deaths and DALYs(Disability-Adjusted Life Years)” for measures; “all locations” for location; “1990–2021” for years; “number, rate and percentage” for metrics; “male, female and both” for sex; “metabolic risks” for risk; and “Age-standardized and all ages” for age. We followed the Guidelines for Accurate and Transparent Health Estimates Reporting guidelines for cross-sectional studies. Besides, integrating metabolic risk into the prognostic staging system for multiple myeloma (such as R-ISS) requires the combination of relevant biomarkers with existing staging factors (such as high-risk cytogenetics, renal function, etc.) to form a model (such as R-ISS-Met), in order to improve the accuracy of prognostic assessment.

### 2.3 Statistical analysis

Employing the data from the GBD database, describe the mortality and DALYs rates of metabolic risk factors associated with multiple myeloma at the global, regional, and national levels from 1990 to 2021. Calculate the age - standardized mortality rate (ASMR) and age - standardized disability - adjusted life - year rate (ASDR) of metabolic risk factors associated with multiple myeloma globally, regionally, and nationally, and plot the world maps of ASMR and ASDR. The EAPC was calculated based on the formula 100* (exp(β) -1), and the 95% CI was obtained from the linear regression model. Calculate the estimated annual percentage change (EAPC) of multiple myeloma attributable to metabolic risk factors at global, regional, and national levels. Plot world maps of EAPC for ASMR and ASDR. Categorize the age distribution of multiple myeloma attributable to metabolic risk factors into 16 groups: 20-24, 25-29, 30-34, 35-39, 40-44, 45-49, 50-54, 55-59, 60-64, 65-69, 70-74, 75-79, 80-84, 85-89, 90-94, and ≥95 years. Compare the age burden composition of metabolic risk factors associated with multiple myeloma across different Socio-demographic Index (SDI) regions from 1990 to 2021. Compare the gender composition of multiple myeloma attributable to metabolic risk factors globally in 2021. All statistics were performed using the R program (Version 4.5.0, R core team) to ensure the consistency of estimated values, including those of the mortality rate and the DALYs rate. As the GBD database officially uses the following formula to calculate the population attributable fraction (PAF) for composite populations, this article does not need to further calculate the confounding variables.


$$FAP_{combined}=1-\prod_{i=1}^{n}\left(1-PAF_{i}\right)$$

## 3 Results

### 3.1 Time and sex trends of multiple myeloma under the influence of metabolic factors from 1990 to 2021

**Figure 1:**
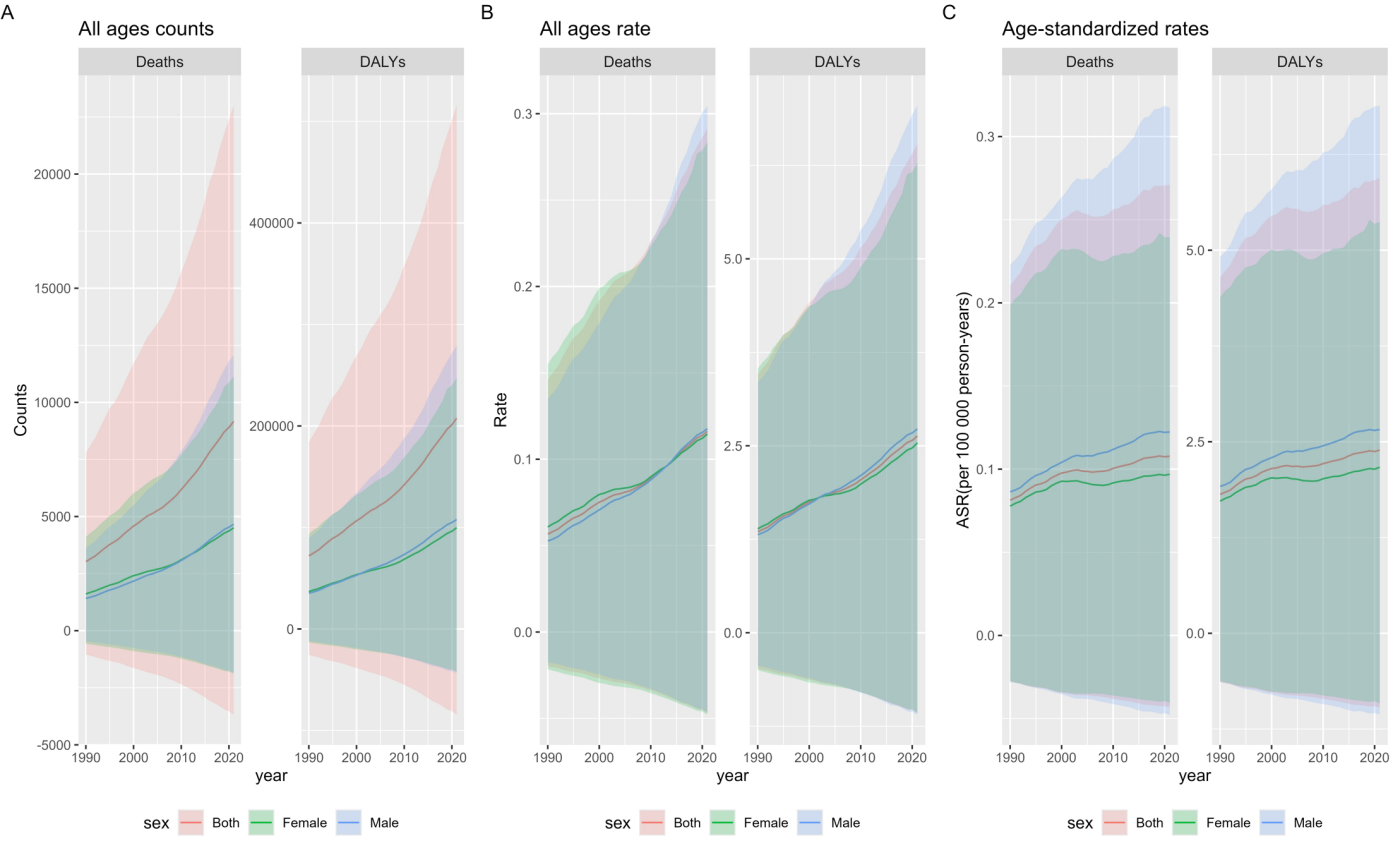
Global Burden of Multiple Myeloma Related to Metabolic Factors from 1990 to 2021: Counts, Rates, and Age-Standardized Measures. **(A)** The counts of deaths and DALYs due to multiple myeloma affected by metabolic factors among all ages and both genders globally from 1990 to 2021. **(B)** The death and DALYs rate of multiple myeloma affected by metabolic factors among all ages and both genders globally from 1990 to 2021. **(C)** The ASMR and ASDR rate of multiple myeloma affected by metabolic factors among all ages and both genders globally from 1990 to 2021. ASMR, age-standardized mortality (death) rate; ASDR, age-standardized disability-adjusted life years rate.

From 1990 to 2021, the global number of deaths from multiple myeloma related to metabolic factors in both sexes across all ages increased threefold, from 3,023 cases (95% UI: -1,046.8–7,782.8) in 1990 to 9,165 cases (95% UI: -3,674.6–22,992.2) in 2021, demonstrating a consistent upward trend (**Supplementary Table 1, pp1**). The disability-adjusted life years (DALYs) for this disease increased 2.8-fold from 1990 to 2021, rising from 72,009.9 (95% UI: -25,461–184,167) in 1990 to 207,634 (95% UI: -84,339.5–515,476.5) in 2021, with a consistent upward trend observed (**Supplementary Table 1, pp2**). The mortality rate of multiple myeloma across all ages exhibited a consistent upward trend over the years, with a flattening of the increase between 2000 and 2010, followed by a marked resurgence after 2021. It nearly doubled from 0.056 (95% UI: -0.019–0.145) in 1990 to 0.116 (95% UI: -0.046–0.291) in 2021, with an overall upward trajectory (**Supplementary Table 1, pp2**). The DALYs rate for this disease demonstrated a notable doubling, increasing from 1.350 (95% UI: -0.477–3.452) in 1990 to 2.631 (95% UI: -1.068–6.532) in 2021 (**Supplementary Table 1, pp2**). The age-standardized mortality rate (ASMR) for this disease demonstrated an overall upward trend (**Supplementary Table 1, pp3**), increasing 1.3-fold from 0.081 (95% UI: -0.027–0.210)/100,000 in 1990 to 0.107 (95% UI: -0.043–0.270)/100,000 in 2021. The age-standardized disability-adjusted life-year rate (ASDR) followed a similar upward trajectory (**Supplementary Table 1, pp3**), increasing 1.3-fold from 1.812 (95% UI: -0.635–4.645) in 1990 to 2.391 (95% UI: -0.968–5.942) in 2021. From 1990 to 2021, both the ASDR and ASMR for this disease were consistently higher in males than in females (**Supplementary Table 1, pp3**). In 2021, the ASMR in males was 1.27-fold higher than in females [0.122 (95% UI: -0.047–0.317)/100,000 vs. 0.096 (95% UI: -0.040–0.239)/100,000]. In 2021, the ASDR in males was 1.22-fold higher than in females [2.660 (95% UI: -1.061–6.885)/100,000 vs. 2.167 (95% UI: -0.914–5.371)/100,000] (**Figure 1**).

### 3.2 Region and sex trends of multiple myeloma from 1990 to 2021

Across all global regions categorized by Socio-demographic Index (SDI) quintiles, the DALYs for multiple myeloma related to metabolic factors exhibited an upward trend. The most pronounced increase in age-standardized DALYs rate occurred in Middle SDI countries, with an estimated annual percentage change (EAPC) of 3.1 (95% CI: 2.97–3.22) for the age-standardized DALYs rate. This rate rose from 0.6 (95% UI: -0.2–1.5)/100,000 in 1990 to 1.5 (95% UI: -0.6–3.9)/100,000 in 2021. The age-standardized DALYs rate increased least in High SDI Countries, with an EAPC of ASDR of 0.18 (95%CI 0.04–0.33), and the age-standardized DALYs rate increased from 4 (95%UI -1.4–10.4) in 1990 to 4.5 (95%UI -2–11.4) in 2021. At the regional level, between 1990 and 2021, the age-standardized DALYs rate for multiple myeloma related to metabolic factors increased in 19 of 21 global geographic regions. Only High-income Asia Pacific and High-income North America exhibited downward trends, with estimated EAPC for the age-standardized DALYs rate of -0.07 (95% CI: -0.19–0.06) and -0.17 (95% CI: -0.35–0.02), respectively. Among regions with upward trends, East Asia experienced the most pronounced increase, with an EAPC of 5.88 (95% CI: 5.3–6.47) for the age-standardized DALYs rate, which rose from 0.1 (95% UI: 0–0.4)/100,000 in 1990 to 0.9 (95% UI: -0.3–2.3)/100,000 in 2021. The age-standardized DALYs rate increased least in Western Europe Countries, with an EAPC of ASDR of 0.49 (95%CI 0.33–0.65), and the rate increased from 4 (95%UI -1.4–10.2)/100,000 in 1990 to 4.7 (95%UI -2–12)/100,000 in 2021.At the gender level, Western Sub-Saharan Africa exhibited a notable disparity in DALYs between males and females, whereas no significant gender differences were observed in other regions (**Table 1**).

**Table 1:** The disease burden of multiple myeloma associated with metabolic risk factors in each region from 1990 to 2021.

Location	Sex	ASDR(per100000), 1990	ASDR(per100000), 2021	EAPC of ASDR(95%CI), 1990-2021	DALY(95%UI) Case,1990	DALY(95%UI) Case,2021
Global	Both	1.8 (-0.6-4.6)	2.4 (-1-5.9)	0.77 (0.68-0.86)	72009.9 (-25461-184167)	207634 (-84339.5-515476.5)
	Male	2.7 (-1.1-6.9)	2.7 (-1-6.8)	0.99 (0.9-1.08)	35226.9 (-11928.9-89978.6)	107846.2 (-43340.5-279020.2)
	Female	1.7 (-0.6-4.4)	2.1 (-0.9-5.4)	0.55 (0.45-0.64)	36783.1 (-13252.6-93521.2)	99787.8 (-42171-247182.6)
High SDI	Both	4 (-1.4-10.4)	4.5 (-2-11.4)	0.18 (0.04-0.33)	44378.7 (-15625.3-113846.9)	91173.5 (-39599-232283.1)
	Male	5.5 (-2.4-13.9)	5.6 (-2.4-14)	0.34 (0.2-0.47)	22796.8 (-8204.9-58266.4)	51331.6 (-22365.2-130482.5)
	Female	3.5 (-1.2-8.9)	3.7 (-1.6-9.2)	-0.07 (-0.22-0.09)	21581.9 (-7702-55521.7)	39841.9 (-17214.8-101368.8)
High-middle SDI	Both	1.8 (-0.6-4.5)	2.6 (-1.1-6.5)	1.21 (1.15-1.27)	17943 (-6537.1-45639.9)	51882.1 (-22283.3-128250.4)
	Male	2.8 (-1.1-7.1)	2.8 (-1.1-7)	1.43 (1.36-1.49)	8030.8 (-2717.2-20093.1)	25262.5 (-10175.1-64610.9)
	Female	1.7 (-0.7-4.5)	2.5 (-1.1-6.1)	0.98 (0.92-1.05)	9912.2 (-3959-25646.2)	26619.5 (-11849.6-65514.3)
Middle SDI	Both	0.6 (-0.2-1.5)	1.5 (-0.6-3.9)	3.1 (2.97-3.22)	2166.6 (-483.6-5557.1)	4819.8 (-1151.6-12666.1)
	Male	1.6 (-0.6-4.1)	1.5 (-0.5-3.8)	3.45 (3.31-3.59)	3028.5 (-894.8-7615)	21609.5 (-7953.5-56116.7)
	Female	0.6 (-0.2-1.6)	1.4 (-0.6-3.6)	2.77 (2.65-2.89)	3456.9 (-1244.3-8968.6)	21896.1 (-8927.9-54277.3)
Low-middle SDI	Both	0.4 (-0.1-1)	1.1 (-0.4-2.8)	3.79 (3.74-3.84)	2387.2 (-674.8-6256.8)	16910.4 (-6031.4-42259.2)
	Male	1.1 (-0.3-2.8)	1 (-0.3-2.7)	4.2 (4.13-4.28)	1035 (-210.2-2697.1)	7844.4 (-2384.9-20413.6)
	Female	0.4 (-0.1-1.1)	1.1 (-0.4-3)	3.43 (3.39-3.47)	1352.1 (-458.3-3708.9)	9065.9 (-3404.4-24280.7)
Low SDI	Both	0.3 (-0.1-0.8)	0.7 (-0.2-1.8)	2.9 (2.76-3.05)	699.5 (-143.1-1951.6)	3875.9 (-1050.4-10326.4)
	Male	0.6 (-0.1-1.6)	0.6 (-0.1-1.5)	3.19 (3.04-3.34)	279.2 (-27.1-794.2)	1656.6 (-318.1-4555.1)
	Female	0.3 (-0.1-1)	0.7 (-0.2-2)	2.66 (2.51-2.8)	420.3 (-103.6-1237)	2219.3 (-696.7-5894.8)
Andean Latin America	Both	1.7 (-0.6-4.5)	3.5 (-1.5-9.1)	2.41 (2.26-2.56)	370.9 (-132-962.9)	2112.5 (-886.6-5521.7)
	Male	3.9 (-1.6-10.4)	3.9 (-1.6-10.6)	2.25 (2.06-2.44)	213.2 (-67.6-568)	1131.5 (-473.7-3048.2)
	Female	1.4 (-0.6-4)	3.2 (-1.4-8.4)	2.61 (2.49-2.73)	157.8 (-65.4-438.6)	981.1 (-428.1-2697.1)
Australasia	Both	4.7 (-1.7-12.2)	6.4 (-3-16)	1.1 (1.01-1.19)	1114.3 (-401.3-2870.3)	3387 (-1549.5-8416.5)
	Male	8 (-3.7-19.9)	8.2 (-3.8-20.5)	1.32 (1.22-1.43)	599.7 (-217.5-1543)	1987.2 (-916.3-4951.2)
	Female	4 (-1.5-10.6)	5.2 (-2.3-13)	0.75 (0.66-0.85)	514.6 (-195.1-1345.3)	1399.8 (-633.4-3503)
Caribbean	Both	2.6 (-0.9-6.6)	4.5 (-1.9-11.5)	1.75 (1.62-1.88)	695.5 (-238.9-1733.8)	2406.8 (-1030.9-6204.9)
	Male	4.4 (-1.8-11.6)	4.4 (-1.7-11)	4.4 (-1.7-11)	300.5 (-95.2-762.1)	1136.5 (-454-2962.2)
	Female	2.9 (-1-7.5)	4.5 (-1.9-11)	1.44 (1.3-1.57)	395 (-140.8-1012.8)	1270.2 (-567.9-3205.4)
Central Asia	Both	0.6 (-0.2-1.4)	1.2 (-0.5-3.1)	2.8 (2.47-3.12)	294.2 (-110.3-727.3)	1109.1 (-494.7-2829.6)
	Male	1.1 (-0.5-3)	1.2 (-0.5-3)	2.59 (2.27-2.91)	134.4 (-45.8-339.1)	482.7 (-205.4-1251.3)
	Female	0.6 (-0.2-1.4)	1.3 (-0.6-3.1)	2.99 (2.63-3.35)	159.7 (-64-408.1)	626.5 (-287.2-1595.1)
Central Europe	Both	2.8 (-1.1-7.1)	4.5 (-2-11.6)	1.41 (1.23-1.6)	4371 (-1741.9-10889.6)	9789.6 (-4444.9-25106.9)
	Male	5.1 (-2.3-13)	5.1 (-2.2-12.7)	1.48 (1.31-1.65)	2190.1 (-878.3-5537.9)	4831 (-2178.7-12383.1)
	Female	2.5 (-1-6.3)	3.9 (-1.8-9.7)	1.35 (1.15-1.55)	2180.9 (-880.7-5459)	4958.6 (-2250.9-12613.5)
Central Latin America	Both	2 (-0.7-5)	3.7 (-1.6-9.3)	1.88 (1.78-1.98)	1768.6 (-650.6-4504.5)	9543.8 (-4267-24165.1)
	Male	4 (-1.7-10.5)	3.8 (-1.6-9.8)	2.26 (2.13-2.38)	842.3 (-287.4-2165.3)	4886.3 (-2036.5-12703.1)
	Female	2 (-0.8-5.1)	3.2 (-1.5-8.1)	1.52 (1.42-1.62)	926.3 (-364.7-2335.2)	4657.5 (-2070.9-11775.2)
Central Sub-Saharan Africa	Both	0.2 (-0.1-0.7)	0.6 (-0.2-1.7)	2.99 (2.72-3.25)	59.7 (-12.7-166.7)	365.8 (-119.2-1040.1)
	Male	0.6 (-0.2-1.7)	0.5 (-0.1-1.5)	2.92 (2.6-3.23)	28.6 (-4.5-80.8)	170.4 (-46.6-489.7)
	Female	0.2 (-0.1-0.7)	0.5 (-0.2-1.5)	3.09 (2.86-3.32)	31.1 (-7.7-92.6)	195.4 (-73.8-545.7)
East Asia	Both	0.1 (0-0.4)	0.9 (-0.3-2.3)	5.88 (5.3-6.47)	1289.7 (-269.6-4258.8)	20160.5 (-6736.8-51230.9)
	Male	1 (-0.3-2.7)	0.9 (-0.3-2.5)	6.33 (5.78-6.87)	663.5 (-102.2-2192)	10905.1 (-3071.1-29536.8)
	Female	0.1 (0-0.4)	0.7 (-0.3-2)	0.7 (-0.3-2)	626.2 (-142.4-2027.7)	9255.5 (-3235.6-25334.6)
Eastern Europe	Both	2 (-0.8-4.9)	3.8 (-1.7-9.3)	2.17 (2-2.34)	5618.1 (-2250.9-13984.2)	13031.9 (-6072.8-32082.2)
	Male	3.4 (-1.5-8.7)	3.5 (-1.5-9)	2.33 (2.12-2.54)	1991.7 (-644.5-4859)	4798.3 (-2104.6-12126.1)
	Female	2.1 (-0.9-5.3)	3.9 (-1.8-9.6)	2.03 (1.87-2.2)	3626.4 (-1630.9-9274.6)	8233.6 (-3873.4-20120)
Eastern Sub-Saharan Africa	Both	0.5 (-0.1-1.4)	1.3 (-0.4-3.5)	3.22 (3.09-3.34)	407.2 (-70.2-1137.6)	2545.3 (-701.5-6596.9)
	Male	1.1 (-0.2-3.1)	1 (-0.2-2.8)	3.32 (3.2-3.44)	158.1 (-15.1-468.3)	1020.1 (-216.1-2887.3)
	Female	0.6 (-0.1-1.7)	1.4 (-0.5-3.8)	3.1 (2.97-3.24)	249 (-56.1-695.7)	1525.2 (-490.8-4106.6)
High-income Asia Pacific	Both	1.1 (-0.2-2.7)	1.1 (-0.3-2.8)	0.77 (0.68-0.86)	2166.6 (-483.6-5557.1)	4819.8 (-1151.6-12666.1)
	Male	1.3 (-0.3-3.4)	1.3 (-0.3-3.3)	0.28 (0.12-0.43)	1023.2 (-206.4-2664.7)	2528.3 (-594.1-6608.7)
	Female	1 (-0.2-2.5)	0.9 (-0.2-2.4)	-0.49 (-0.61–0.37)	1143.4 (-280.4-2893.8)	2291.5 (-632.6-6107.7)
High-income North America	Both	6.4 (-2.6-16.3)	6.6 (-3-16.4)	-0.17 (-0.35-0.02)	21714.2 (-8805.1-55564.6)	42763 (-19449.8-106584.3)
	Male	8.3 (-3.8-20.6)	8.3 (-3.8-20.4)	0.05 (-0.13-0.22)	11333.5 (-4523.1-28495.8)	24674.5 (-11301.7-61621.9)
	Female	5.4 (-2.2-13.7)	5.2 (-2.3-12.7)	-0.47 (-0.67–0.28)	10380.7 (-4249.9-26465.3)	18088.5 (-8148.1-45023.1)
North Africa and Middle East	Both	1.4 (-0.6-3.8)	3 (-1.4-7.7)	2.48 (2.41-2.56)	2578.1 (-1137-6969.7)	14597.9 (-6942.8-37405.1)
	Male	3.1 (-1.3-8)	3 (-1.3-7.5)	2.87 (2.77-2.97)	1219.8 (-384.8-3162.1)	7668.6 (-3394.6-19781.5)
	Female	1.5 (-0.7-4.5)	2.8 (-1.4-7.4)	2.1 (2.04-2.15)	1358.4 (-599.8-4067.7)	6929.3 (-3390.3-18382.6)
Oceania	Both	3 (-1.4-7.7)	0.8 (-0.3-1.9)	1.16 (1.07-1.25)	18.3 (-7.5-47.9)	65.4 (-30-166.6)
	Male	0.7 (-0.3-1.7)	0.7 (-0.3-1.7)	0.52 (0.43-0.62)	9.5 (-3.6-25.7)	28.9 (-11.3-73.2)
	Female	0.5 (-0.2-1.4)	0.9 (-0.4-2.2)	1.79 (1.65-1.92)	8.9 (-3.9-24.6)	36.5 (-18.6-96.5)
South Asia	Both	0.2 (0-0.7)	0.9 (-0.3-2.3)	4.34 (4.29-4.39)	1563 (-263.9-4135.5)	14287.6 (-4397.2-36078.3)
	Male	0.9 (-0.2-2.4)	0.9 (-0.2-2.3)	4.51 (4.47-4.55)	780 (-96-2153)	7098.6 (-1762.7-18751.3)
	Female	0.3 (-0.1-0.7)	0.9 (-0.3-2.3)	4.16 (4.09-4.24)	782.9 (-168.3-2228.8)	7189 (-2315.3-20010.8)
Southeast Asia	Both	0.2 (0-0.4)	0.5 (-0.2-1.4)	3.73 (3.61-3.85)	465.9 (-119.2-1249.8)	3711.4 (-1269.1-10082.3)
	Male	0.4 (-0.1-1.3)	0.4 (-0.1-1.2)	3.75 (3.62-3.88)	188.6 (-27.8-547.2)	1521.1 (-390.7-4379.3)
	Female	0.2 (-0.1-0.5)	0.6 (-0.2-1.7)	3.72 (3.6-3.84)	277.4 (-78.3-763.8)	2190.3 (-871.3-6388.4)
Southern Latin America	Both	4 (-1.6-10.4)	4.8 (-2.1-12.2)	0.64 (0.45-0.82)	1901.8 (-733.8-4887.5)	4126.4 (-1820.4-10460)
	Male	5.7 (-2.6-14.3)	6 (-2.8-15.3)	1.01 (0.85-1.18)	911.1 (-318.9-2359)	2197.1 (-989.3-5557.3)
	Female	3.8 (-1.6-9.8)	4.3 (-1.9-11)	0.25 (0.03-0.48)	990.7 (-404.1-2530.2)	1929.3 (-835.5-4891.8)
Southern Sub-Saharan Africa	Both	2.6 (-1-6.5)	5.7 (-2.5-14.8)	2.7 (2.58-2.81)	753.4 (-300.9-1891.7)	3544.7 (-1595-9269.5)
	Male	5 (-2.1-12.5)	4.8 (-2-12.7)	2.85 (2.59-3.12)	270.4 (-81-696.8)	1355.1 (-556-3372)
	Female	3 (-1.5-7.3)	5.9 (-2.7-14.5)	2.63 (2.52-2.74)	482.9 (-233.9-1169.9)	2189.6 (-1008.2-5455.4)
Tropical Latin America	Both	2.1 (-0.7-5.4)	4.1 (-1.7-10.5)	2.04 (1.87-2.21)	2055.8 (-713.1-5358.5)	10681.5 (-4574.1-27467.2)
	Male	4.4 (-1.9-11.1)	4.4 (-1.8-11.1)	2.58 (2.4-2.76)	946.8 (-309.9-2447.9)	5318.3 (-2244-13368.1)
	Female	2.2 (-0.8-5.7)	3.6 (-1.6-9.5)	1.56 (1.39-1.73)	1108.9 (-405.4-2891.9)	5363.2 (-2306.2-13680.8)
Western Europe	Both	4 (-1.4-10.2)	4.7 (-2-12)	0.49 (0.33-0.65)	22581.2 (-7755.5-58081.3)	42948.6 (-17872.1-109646.9)
	Male	5.7 (-2.4-14.6)	6 (-2.5-15.1)	0.63 (0.48-0.78)	11345.8 (-3852.8-29321.4)	23639.5 (-9834.9-59859.1)
	Female	3.4 (-1.2-8.8)	4 (-1.7-10.1)	0.26 (0.09-0.43)	11235.4 (-3832.5-29308.6)	19309 (-8037.2-49701.2)
Western Sub-Saharan Africa	Both	0.2 (-0.1-0.7)	0.8 (-0.3-2.3)	3.99 (3.85-4.12)	222.3 (-56.4-632.1)	1635.3 (-529.9-4823.4)
	Male	0.5 (-0.1-1.2)	0.4 (-0.1-1.2)	3.62 (3.55-3.69)	75.9 (-19.2-202.6)	467.1 (-143.6-1253.7)
	Female	0.3 (-0.1-1)	1 (-0.4-2.8)	4.03 (3.87-4.2)	146.4 (-41.9-429.1)	1168.2 (-385.6-3672.3)

### 3.3 The disease burden of multiple myeloma associated with metabolic risk factors at the national level

**Figure 2:**
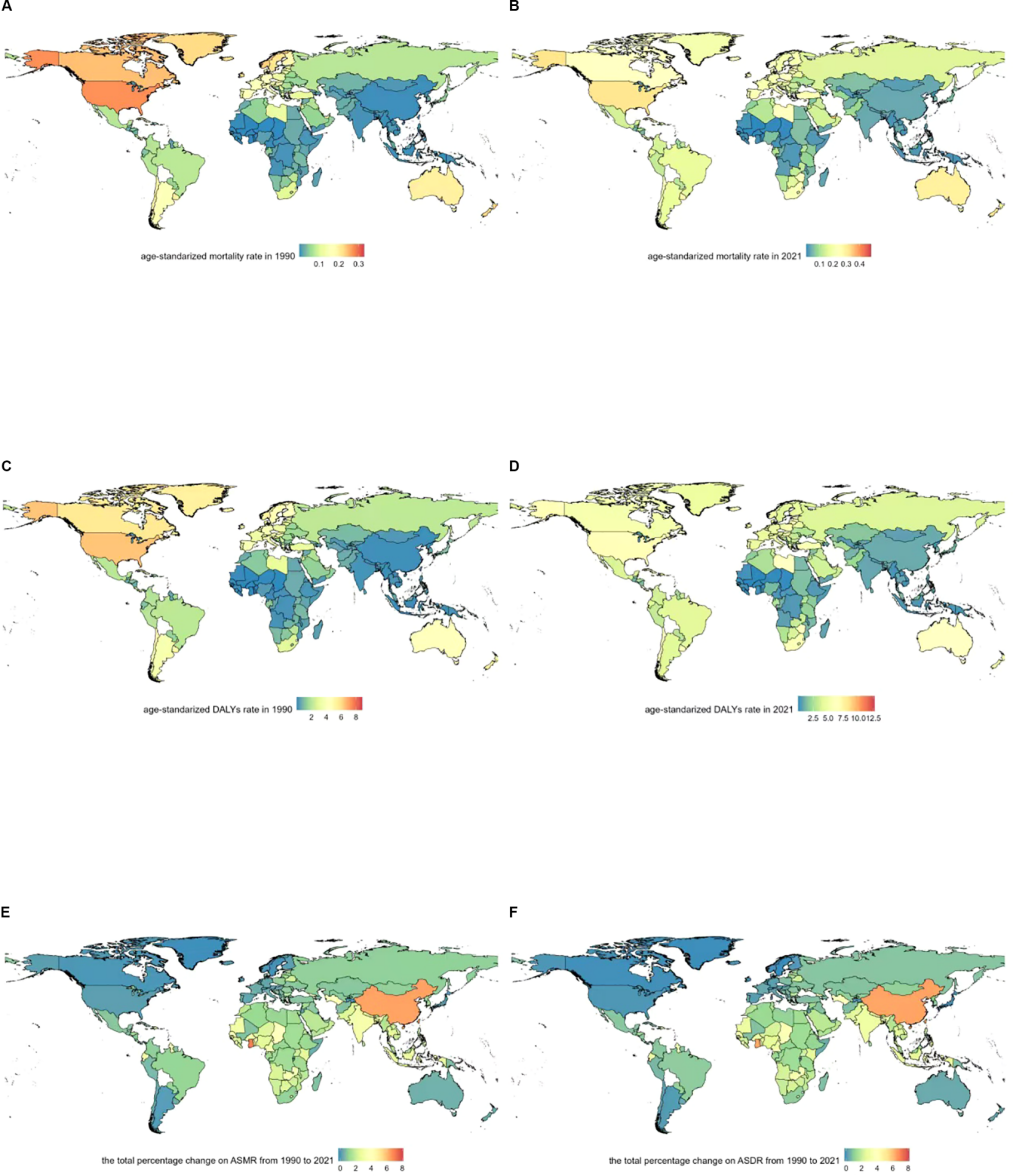
Geographical distribution of age-standardized rates of metabolic risk factors associated with multiple myeloma. **(A)** Age-standardized mortality rate in 1990. **(B)** Age-standardized mortality rate in 2021. **(C)** Age-standardized DALYs rate in 1990. **(D)** Age-standardized DALYs rate in 2021. **(E)** The total percentage change on ASMR from 1990 to 2021. **(F)** The total percentage change on ASDR from 1990 to 2021.

In 2021, the United States, China, and Germany reported the highest numbers of deaths from multiple myeloma related to metabolic factors: 1,935.8 (95% UI: -851.0–4,872.2) in the U.S., 710.0 (95% UI: -231.0–1,819.7) in China, and 445.9 (95% UI: -174.5–1,187.5) in Germany (**Supplementary Table 1, pp4–13**). The highest age-standardized mortality rates occurred in the Commonwealth of the Bahamas (0.48 [95% UI: -0.21–1.22]/100,000), Principality of Monaco (0.41 [95% UI: -0.18–1.14]/100,000), and United Arab Emirates (0.35 [95% UI: -0.17–0.87]/100,000). The lowest age-standardized mortality rates were found in Mali (2.17E-06 [95% UI: -5.10E-07~6.27E-06]/100,000), Niger (0.001852 [95% UI: -0.00043–0.006015]/100,000), and Burkina Faso (0.002055 [95% UI: -0.00043–0.006015]/100,000) (**Supplementary Table 1, pp14–23**). The highest age-standardized DALYs rates were recorded in the Commonwealth of the Bahamas (12.7 [95% UI: -5.78–32.1]/100,000), Principality of Monaco (8.96 [95% UI: -4.15–26.1]/100,000), and Jamaica (8.16 [95% UI: -3.56–21.07]/100,000). The lowest age-standardized DALYs rates were observed in Mali (5.44E-05 [95% UI: -1.34E-05–0.000158]/100,000), Niger (0.046 [95% UI: -0.011–0.156]/100,000), and Burkina Faso (0.053 [95% UI: -0.008–0.164]/100,000) (**Supplementary Table 1, pp24–33**).The age-standardized mortality rate for multiple myeloma relevant to metabolic factors showed an overall upward trend across 204 countries and territories. The most substantial increase occurred in Ghana, where the rate rose from 0.00952 (95% UI: -0.00026–0.002778)/100,000 in 1990 to 0.007615 (95% UI: -0.00204–0.0248)/100,000 in 2021, with an EAPC of 7.52 (95% CI: 7.31–7.73). Georgia experienced an increase from 0.02 (95% UI: -0.008–0.057)/100,000 in 1990 to 0.12 (95% UI: -0.04–0.30)/100,000 in 2021, with an EAPC of 6.73 (95% CI: 5.97–7.50). Equatorial Guinea witnessed an increase from 0.01 (95% UI: -0.003–0.03)/100,000 in 1990 to 0.07 (95% UI: -0.03–0.21)/100,000 in 2021, with an EAPC of 6.54 (95% CI: 6.29–6.79). However, three countries exhibited downward trends in age-standardized mortality rate: Northern Mariana Islands, where the rate declined from 0.142 (95% UI: -0.06–0.36)/100,000 in 1990 to 0.140 (95% UI: -0.06–0.35)/100,000 in 2021, with an EAPC of -0.46 (95% CI: -0.69–0.23). Greenland experienced a decline from 0.22 (95% UI: -0.08–0.26)/100,000 in 1990 to 0.19 (95% UI: -0.08–0.51)/100,000 in 2021, with an EAPC of -0.21 (95% CI: -0.38–0.04). Japan experienced a decline from 0.048 (95% UI: -0.012–0.126)/100,000 in 1990 to 0.051 (95% UI: -0.067–0.374)/100,000 in 2021, with an EAPC of -0.11 (95% CI: -0.24–0.03) (**Figures 2A, B**; **pp14–23, Supplementary Table 1, pp34–52**). The ASDR for multiple myeloma related to metabolic factors showed an upward trend across 204 countries and territories. The most substantial increase was observed in Ghana, where the rate rose from 0.025 (95% UI: -0.007–0.072)/100,000 in 1990 to 0.188 (95% UI: -0.052–0.601)/100,000 in 2021, with an EAPC of 7.22 (95% CI: 7.01–7.42). Georgia saw an increase from 0.689 (95% UI: -0.259–1.762)/100,000 in 1990 to 3.495 (95% UI: -1.424–8.975)/100,000 in 2021, with an EAPC of 6.59 (95% CI: 5.83–7.34). Turkmenistan witnessed an increase from 0.559 (95% UI: -0.209–1.412)/100,000 in 1990 to 2.662 (95% UI: -1.068–7.176)/100,000 in 2021, with an EAPC of 6.53 (95% CI: 5.94–7.14). ASDR declines were observed in several countries and regions. The most pronounced decrease occurred in the Northern Mariana Islands, where the rate dropped from 3.721 (95% UI: -1.761–9.841)/100,000 in 1990 to 3.637 (95% UI: -1.833–9.282)/100,000 in 2021, with an EAPC of -0.44 (95% CI: -0.69–0.19). Japan saw a decrease from 1.135 (95% UI: -0.251–2.941)/100,000 in 1990 to 1.063 (95% UI: -0.261–2.744)/100,000 in 2021, with an EAPC of -0.43 (95% CI: -0.26–0.29). Canada experienced a decline from 5.664 (95% UI: -2.108–14.550)/100,000 in 1990 to 5.216 (95% UI: -2.364–13.41)/100,000 in 2021, with an EAPC of -0.33 (95% CI: -0.46–0.19) (**Figures 2C, D**; **pp24–33, Supplementary Table 1, pp53–71**).

### 3.4 The disease burden of multiple myeloma associated with metabolic risk factors in different periods

**Figure 3:**
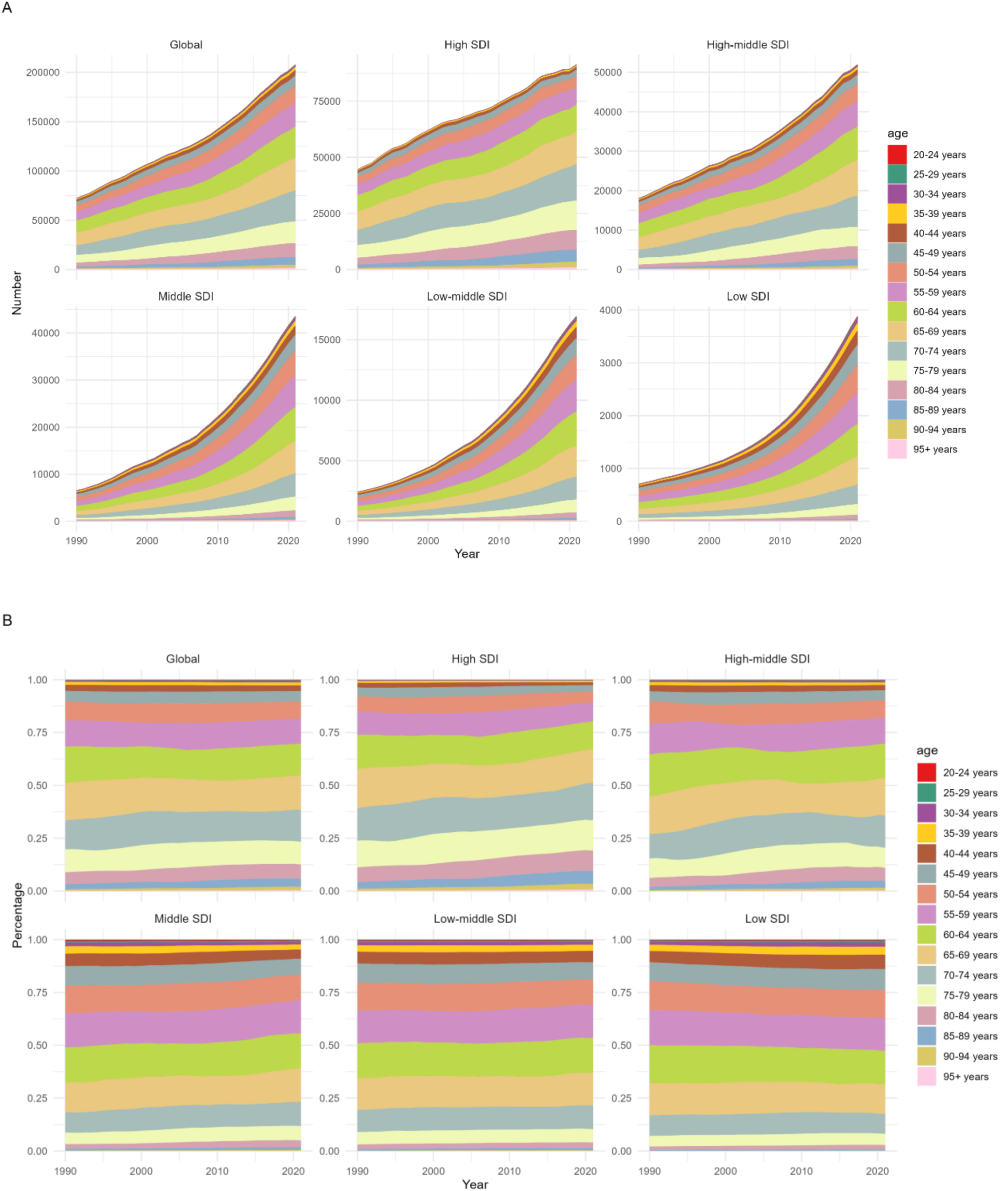
The disease burden of multiple myeloma associated with metabolic risk factors by Age and SDI,1990-2021. **(A)** Number of DALYs of Multiple Myeloma Attributable to Metabolic Factors by Age and SDI,1990-2021. **(B)** Percentage of DALYs of Multiple Myeloma Relevant to Metabolic Factors by Age and SDI,1990-2021.

From 1990 to 2021, the disease burden of multiple myeloma relevant to metabolic factors demonstrated a universal increase in magnitude across different SDI regions. Notably, the older age group (70–74 years and above) exhibited growth in both the absolute burden and its proportional share of the total, indicating that metabolic factors impose a growing disease burden on older populations and elevate their contribution to the overall burden (**Figure 3**).

### 3.5 The impact of age and gender on the disease burden of multiple myeloma associated with metabolic factors 2021

**Figure 4:**
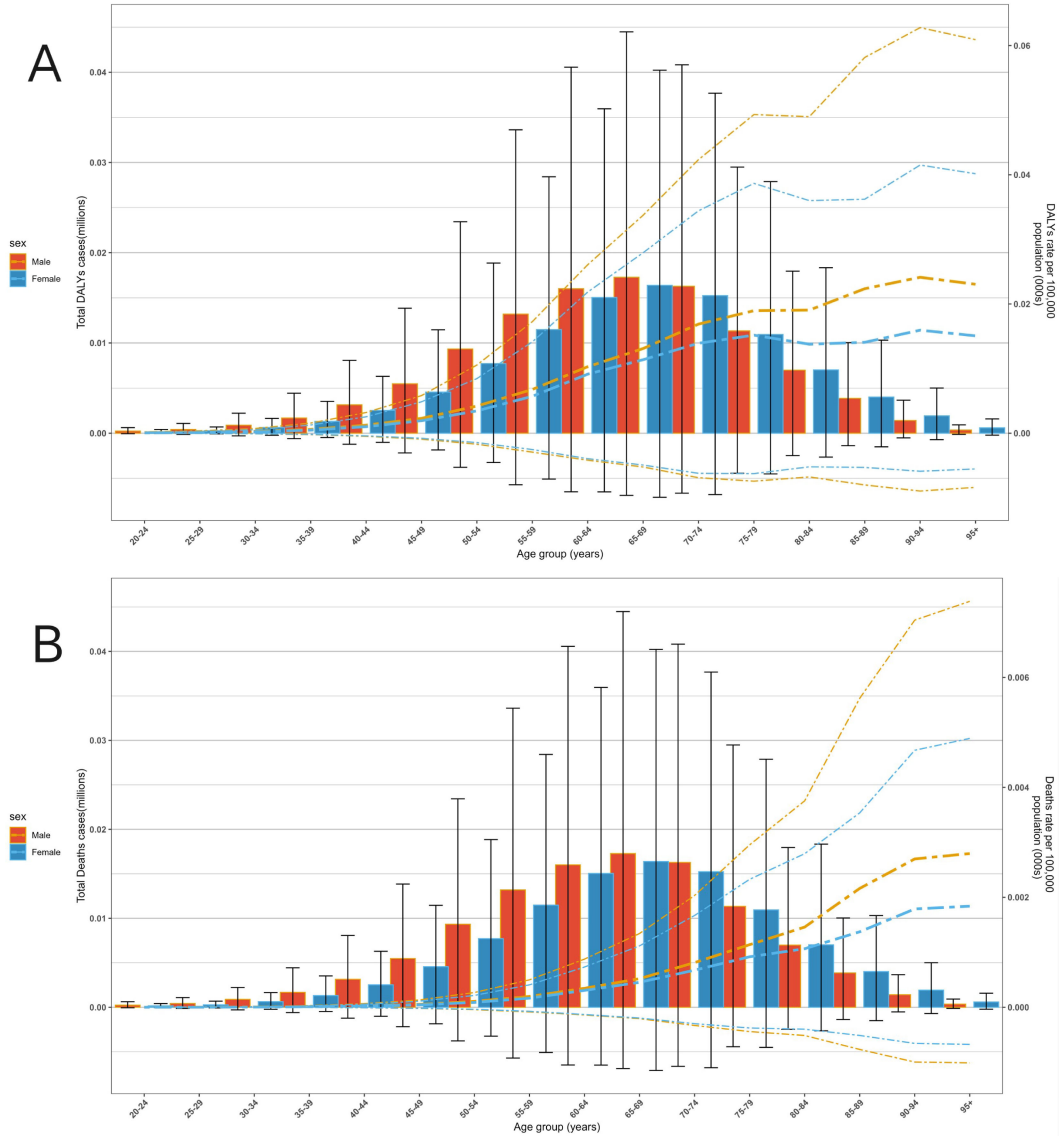
The disease burden of multiple myeloma associated with metabolic factors across different ages and genders globally in 2021. **(A)** DALYs level. **(B)** Deaths level.

Regarding age trends, both the DALYs rate and Deaths rate of multiple myeloma attributable to metabolic factors increased significantly with age. The burden was minimal in younger age groups (20–40 years) but rose rapidly from 50–54 years onward, with a particularly pronounced increase after 70–74 years. Regarding gender disparities, males exhibited slightly higher total DALYs and DALYs rates compared to females, with males showing significantly higher overall values than females in the advanced age group of 70–74 years and older (**Figure 4**).

## 4 Discussion

Multiple myeloma (MM) represents 10–15% of hematological malignancies. The biological crosstalk between metabolic syndrome and multiple myeloma (MM) involves multi-dimensional molecular interactions: obesity-induced bone marrow adipocytes (BMAs) secrete adipokines such as adiponectin. Studies by Zhaoyun Liu et al. (7) demonstrated that adiponectin may inhibit the differentiation and maturation of osteoclasts in MM patients by upregulating the expression of AdipoR1 while downregulating the phosphorylation levels of mTOR and 4EBP1. Furthermore, serine, a key amino acid involved in signal regulation, is closely linked to the progression of metabolic syndrome (8). Research by Chunmei Kuang et al. (9) indicated that excessive serine in the bone marrow microenvironment impairs megakaryopoiesis and thrombopoiesis in MM. Additionally, obesity has been shown to increase the risk of MM development and the progression from pre-myeloma to overt MM (10). Dyslipidemia promotes lipid uptake through the aberrant expression of LDL receptors (LDLR) and syndecan-1 on MM cells, while obesity-induced BMA remodeling inhibits apoptosis and exacerbates osteolytic lesions via the secretion of leptin and resistin (11). Collectively, these mechanisms reveal that metabolic syndrome systemically drives the initiation and progression of MM.

Leveraging GBD data (1990–2021), we observed significant increases in both the disability-adjusted of life years (DALYs) rate and mortality rate of MM relevant to metabolic risk factors. Therefore, characterizing the global burden of MM relevant to metabolic risk factors can inform the development of targeted prevention and treatment strategies across regions, holding significant implications for epidemiology and preventive medicine.

The global disease burden of MM relevant to metabolic factors showed a marked escalation from 1990 to 2021. The number of deaths tripled, and DALYs increased 2.8-fold, with significant rises in mortality rate, DALYs rate, Age-Standard Mortality Rate (ASMR), and Age-Standard Death Rate (ASDR). While the upward trajectory of mortality rate temporarily plateaued during 2000–2010, it reaccelerated thereafter. This shift is primarily attributed to global population growth and accelerated aging during 2000–2010, which expanded the pool of susceptible individuals (12, 13); Concurrently, economic development has been associated with unhealthy lifestyles—including high-calorie/high-fat diets and decreased physical activity—that relate to metabolic dysfunction, thereby exacerbating MM incidence (14, 15). Males exhibited significantly higher ASDR and ASMR for metabolic-factor-relevant MM than females, strongly linked to gender-specific lifestyle disparities. Males typically exhibit higher prevalence of metabolism-related risk behaviors (e.g., unhealthy diets, inadequate physical activity), which further elevate disease risk and burden (16–18). In addition, studies by Brigitte Sola et al. (19) have shown that estrogen has the effect of restricting the proliferation of MM cells, which may also be one of the reasons why the disease burden in males is higher than that in females.

Across SDI quintiles, the global disease burden of metabolic-factor-relevant MM exhibits striking regional and gender disparities. The marked rise in ASDR rates in Middle SDI countries is likely linked to accelerated population aging, suboptimal economic development, and limited healthcare accessibility (20, 21). The modest ASDR increase in High SDI countries is attributed to therapeutic advancements, including the adoption of proteasome inhibitors and immunomodulatory drugs (such as G Protein-Coupled Receptor Class C Group 5 Member D) (2, 21–24). Immunomodulatory drugs combat MM by suppressing immune escape (25, 26) while proteasome inhibitors exert therapeutic effects through mechanisms such as inducing tumor cell apoptosis and inhibiting the NF-κB signaling pathway (27–29). Since 2006, new drugs have significantly prolonged the survival period of MM patients in the West. However, from 1990 to 2021, the improvement in the global burden of MM and the progress in treatment did not match. This lag was caused by factors such as population structure, medical equity, treatment toxicity, and data methods. Geographically, the ASDR increase in most regions correlates with the proliferation of high-calorie, low-fiber diets and inadequate physical activity; High-income Asia Conversely, ASDR declines in High-income Asia Pacific and High-income North American regions are strongly associated with advanced therapeutic interventions (10, 17, 30). For instance, countries like Australia, New Zealand, and the United States report lower mortality rates among patients receiving initial therapy with novel agents (thalidomide, lenalidomide, or bortezomib) (31–33).

Data reveal striking heterogeneity in the disease burden of metabolic-factor-relevant MM across countries from 1990 to 2021. The United States, China, and Germany recorded the highest death tolls, likely linked to their large population sizes and aging demographics. Countries like the Commonwealth of the Bahamas and Principality of Monaco exhibited high age-standardized mortality and DALYs rates, whereas Mali and Niger showed lower rates—correlating with economic status and population metabolic health. Underdiagnosis in less developed nations may stem from limited healthcare resources (13, 34, 35).

From 1990 to 2021, ASDR increased in 190 out of 204 countries and regions, with notable growth in Ghana, Georgia, and other countries. In Ghana, monoclonal gammopathy is not included in routine physical examinations, possibly due to insufficient awareness among primary healthcare institutions and a lack of national testing facilities, which are also one of the reasons for the increased disease burden in the region (36). In Georgia, contributing factors likely include suboptimal fruit/vegetable consumption and high overweight/obesity prevalence among a substantial portion of the population (37); Additionally, the region’s reliance on complementary and alternative medicine (CAM)—which remains outside the formal healthcare system—may further exacerbate disease burden (38, 39). Declines in select countries like Japan are attributed to advanced therapeutic interventions, such as triplet regimens incorporating Carfilzomib, Ixazomib, Elotuzumab, and Daratumumab (17, 40). Collectively, the disease burden of metabolic-factor-relevant MM is shaped by population demographics, economic status, lifestyle patterns, and healthcare interventions, necessitating tailored prevention and control strategies across nations (16, 41).

Across SDI regions, the disease burden of metabolic-factor-relevant multiple myeloma (MM) demonstrated an overall upward trajectory in magnitude from 1990 to 2021, with the oldest age group (70–74 years and above) experiencing sustained increases in both absolute burden and proportional share of the total (16). This suggests that the influence of metabolic factors on MM in older adults has grown more pronounced over time, with their burden escalating and their contribution to the overall disease burden becoming increasingly significant (42–44).

Data from 2021 indicate a positive correlation between metabolic-factor-relevant multiple myeloma (MM) burden and age. Burden remained minimal in younger age groups (20–40 years), accelerated after 50–54 years, and was most pronounced in those aged 70–74 years and older, underscoring higher disease risk and severity from metabolic factors in older adults (16). Gender-wise, males exhibited higher total DALYs and DALYs rates than females, with significantly greater burden observed in males aged 70–74 years and older (45, 46). This highlights a heavier MM burden from metabolic factors in older males. Overall, older male populations should be prioritized in the prevention and control of metabolic factor-related multiple myeloma.

When patients have high blood sugar levels, society can provide systematic public health interventions (such as free blood sugar monitoring, subsidies for essential medications, etc.). The feasibility of these measures depends on the priority of resource allocation (47). Furthermore, studies conducted by YuCheng Chang (48) et al. and Nicholas Grandhi (49) et al. have revealed that glucagon-like peptide-1 (GLP-1) has a highly significant positive effect on patients with diabetes and multiple myeloma. This strategy not only optimizes blood sugar control but also, by inhibiting the tumor microenvironment and protecting heart and kidney functions, has the potential to become a comprehensive treatment plan for such patients. More clinical studies are needed to verify its efficacy and long-term safety in the future.

This study’s reliance on the GBD database entails limitations: GBD employed a multi-level methodology framework, integrating causal inference models with dynamic data calibration techniques, to systematically eliminate the confounding effects between risk factors and disease burden. Therefore, in this study, there is no need to conduct further analysis on the confounding variables. Findings should be interpreted cautiously and contextualized with complementary data and regional specifics. Moreover, health authorities and policymakers should prioritize implementing preventive measures and enhancing quality of life for patients, as underscored by the study’s findings. In high-risk regions, promoting healthy eating habits, regular physical activity, and weight management is critical for at-risk populations. Regular medical screenings, health education, and training programs are essential for early detection and prevention. Furthermore, in regions with a medium SDI, a high body mass index (BMI) has been identified as a key modifiable risk factor that significantly contributes to the burden of MM. Therefore, implementing public health measures focused on weight management, promoting lifestyle changes such as low-salt and low-fat diets, and enhancing physical exercise are of utmost importance for preventing MM before it occurs, improving prognosis, enhancing the quality of life of patients, and reducing the disease burden (50–52).

This study offers critical insights into the global distribution of MM burden. The research highlights striking heterogeneities in metabolic-factor-relevant MM burden across age groups, genders, and regions, alongside an overall upward trajectory. The analysis underscores the urgency of implementing preventive strategies like weight management and lifestyle interventions, and emphasizes the need for targeted prevention and control measures in high-risk populations and regions with elevated burden. The findings offer a scientific foundation for reducing risk factors, stratifying risk, and informing early intervention in MM management, particularly in regions with high disease burden and limited healthcare resources. Clinicians need to incorporate metabolic factors into the entire cycle of management for multiple myeloma (MM) diagnosis and treatment. By integrating biomarkers, targeted therapies, regional strategies and global collaboration, a paradigm shift from disease control to prevention can be achieved.

## Data Availability

The datasets presented in this study can be found in online repositories. The names of the repository/repositories and accession number(s) can be found in the article/**Supplementary Material**.
