# Supplementary material for: Global, regional, and national metabolic risk factors associated with multiple myeloma, 1990-2021: analysis via the global burden of disease study 2021
Source: Front Oncol. 2025 Sep 9;15:1659527. doi: 10.3389/fonc.2025.1659527 (PMC12455239; doi:10.3389/fonc.2025.1659527)
Supplement: Supplementary file 1 [file Table1.docx]

| measure | location | sex | age | cause | rei | metric | year | val | upper | lower |
| --- | --- | --- | --- | --- | --- | --- | --- | --- | --- | --- |
| Deaths | Global | Male | All ages | Multiple myeloma | Metabolic risks | Number | 1990 | 1414.052 | 3625.691 | -472.916 |
| Deaths | Global | Female | All ages | Multiple myeloma | Metabolic risks | Number | 1990 | 1609.435 | 4105.233 | -569.739 |
| Deaths | Global | Both | All ages | Multiple myeloma | Metabolic risks | Number | 1990 | 3023.487 | 7782.892 | -1046.84 |
| Deaths | Global | Male | All ages | Multiple myeloma | Metabolic risks | Number | 2021 | 4658.553 | 12060.21 | -1832.37 |
| Deaths | Global | Female | All ages | Multiple myeloma | Metabolic risks | Number | 2021 | 4506.927 | 11140.6 | -1878.68 |
| Deaths | Global | Both | All ages | Multiple myeloma | Metabolic risks | Number | 2021 | 9165.48 | 22992.25 | -3674.66 |

**Figure 1A** The counts of deaths due to multiple myeloma affected by metabolic factors among all ages and both genders globally from 1990 to 2021

**Figure 1A** The counts of DALYs of multiple myeloma affected by metabolic factors among all ages and both genders globally from 1990 to 2021

| measure | location | sex | age | cause | rei | metric | year | val | upper | lower |
| --- | --- | --- | --- | --- | --- | --- | --- | --- | --- | --- |
| DALYs (Disability-Adjusted Life Years) | Global | Male | All ages | Multiple myeloma | Metabolic risks | Number | 1990 | 35226.85 | 89978.56 | -11928.9 |
| DALYs (Disability-Adjusted Life Years) | Global | Female | All ages | Multiple myeloma | Metabolic risks | Number | 1990 | 36783.07 | 93521.24 | -13252.6 |
| DALYs (Disability-Adjusted Life Years) | Global | Both | All ages | Multiple myeloma | Metabolic risks | Number | 1990 | 72009.92 | 184167 | -25461 |
| DALYs (Disability-Adjusted Life Years) | Global | Male | All ages | Multiple myeloma | Metabolic risks | Number | 2021 | 107846.2 | 279020.2 | -43340.5 |
| DALYs (Disability-Adjusted Life Years) | Global | Female | All ages | Multiple myeloma | Metabolic risks | Number | 2021 | 99787.78 | 247182.6 | -42171 |
| DALYs (Disability-Adjusted Life Years) | Global | Both | All ages | Multiple myeloma | Metabolic risks | Number | 2021 | 207634 | 515476.5 | -84339.5 |

| measure | location | sex | age | cause | rei | metric | year | val | upper | lower |
| --- | --- | --- | --- | --- | --- | --- | --- | --- | --- | --- |
| Deaths | Global | Male | All ages | Multiple myeloma | Metabolic risks | Rate | 1990 | 0.05265 | 0.134998 | -0.01761 |
| Deaths | Global | Female | All ages | Multiple myeloma | Metabolic risks | Rate | 1990 | 0.060782 | 0.155038 | -0.02152 |
| Deaths | Global | Both | All ages | Multiple myeloma | Metabolic risks | Rate | 1990 | 0.056687 | 0.145921 | -0.01963 |
| Deaths | Global | Male | All ages | Multiple myeloma | Metabolic risks | Rate | 2021 | 0.117658 | 0.304598 | -0.04628 |
| Deaths | Global | Female | All ages | Multiple myeloma | Metabolic risks | Rate | 2021 | 0.114623 | 0.283334 | -0.04778 |
| Deaths | Global | Both | All ages | Multiple myeloma | Metabolic risks | Rate | 2021 | 0.116146 | 0.29136 | -0.04657 |

**Figure 1B** The death rate of multiple myeloma affected by metabolic factors among all ages and both genders globally from 1990 to 2021

**Figure 1B** The DALYs rate of multiple myeloma affected by metabolic factors among all ages and both genders globally from 1990 to 2021

| measure | location | sex | age | cause | rei | metric | year | val | upper | lower |
| --- | --- | --- | --- | --- | --- | --- | --- | --- | --- | --- |
| DALYs (Disability-Adjusted Life Years) | Global | Male | All ages | Multiple myeloma | Metabolic risks | Rate | 1990 | 1.311624 | 3.35023 | -0.44416 |
| DALYs (Disability-Adjusted Life Years) | Global | Female | All ages | Multiple myeloma | Metabolic risks | Rate | 1990 | 1.389151 | 3.531929 | -0.5005 |
| DALYs (Disability-Adjusted Life Years) | Global | Both | All ages | Multiple myeloma | Metabolic risks | Rate | 1990 | 1.350113 | 3.452944 | -0.47737 |
| DALYs (Disability-Adjusted Life Years) | Global | Male | All ages | Multiple myeloma | Metabolic risks | Rate | 2021 | 2.723807 | 7.047046 | -1.09462 |
| DALYs (Disability-Adjusted Life Years) | Global | Female | All ages | Multiple myeloma | Metabolic risks | Rate | 2021 | 2.537862 | 6.286496 | -1.07252 |
| DALYs (Disability-Adjusted Life Years) | Global | Both | All ages | Multiple myeloma | Metabolic risks | Rate | 2021 | 2.631158 | 6.532169 | -1.06876 |

**Figure 1C** The ASMR rate of multiple myeloma affected by metabolic factors among all ages and both genders globally from 1990 to 2021

| measure | location | sex | age | cause | rei | metric | year | val | upper | lower |
| --- | --- | --- | --- | --- | --- | --- | --- | --- | --- | --- |
| Deaths | Global | Male | Age-standardized | Multiple myeloma | Metabolic risks | Rate | 1990 | 0.086354 | 0.22289 | -0.02817 |
| Deaths | Global | Female | Age-standardized | Multiple myeloma | Metabolic risks | Rate | 1990 | 0.077747 | 0.198514 | -0.02736 |
| Deaths | Global | Both | Age-standardized | Multiple myeloma | Metabolic risks | Rate | 1990 | 0.081359 | 0.210152 | -0.02769 |
| Deaths | Global | Male | Age-standardized | Multiple myeloma | Metabolic risks | Rate | 2021 | 0.122367 | 0.317173 | -0.04759 |
| Deaths | Global | Female | Age-standardized | Multiple myeloma | Metabolic risks | Rate | 2021 | 0.096915 | 0.239548 | -0.04037 |
| Deaths | Global | Both | Age-standardized | Multiple myeloma | Metabolic risks | Rate | 2021 | 0.107811 | 0.270973 | -0.04307 |

| measure | location | sex | age | cause | rei | metric | year | val | upper | lower |
| --- | --- | --- | --- | --- | --- | --- | --- | --- | --- | --- |
| DALYs (Disability-Adjusted Life Years) | Global | Male | Age-standardized | Multiple myeloma | Metabolic risks | Rate | 1990 | 1.917711 | 4.915627 | -0.64113 |
| DALYs (Disability-Adjusted Life Years) | Global | Female | Age-standardized | Multiple myeloma | Metabolic risks | Rate | 1990 | 1.724559 | 4.385875 | -0.62021 |
| DALYs (Disability-Adjusted Life Years) | Global | Both | Age-standardized | Multiple myeloma | Metabolic risks | Rate | 1990 | 1.812155 | 4.645054 | -0.63541 |
| DALYs (Disability-Adjusted Life Years) | Global | Male | Age-standardized | Multiple myeloma | Metabolic risks | Rate | 2021 | 2.660188 | 6.885548 | -1.06114 |
| DALYs (Disability-Adjusted Life Years) | Global | Female | Age-standardized | Multiple myeloma | Metabolic risks | Rate | 2021 | 2.167712 | 5.371198 | -0.91438 |
| DALYs (Disability-Adjusted Life Years) | Global | Both | Age-standardized | Multiple myeloma | Metabolic risks | Rate | 2021 | 2.391285 | 5.94228 | -0.96859 |

**Figure 1C** The ASDR rate of multiple myeloma affected by metabolic factors among all ages and both genders globally from 1990 to 2021

The Deaths number of multiple myeloma affected by metabolic factors among all ages and both genders nationally in 2021

| measure | location | sex | age | cause | rei | metric | year | val | upper | lower |
| --- | --- | --- | --- | --- | --- | --- | --- | --- | --- | --- |
| Deaths | United States of America | Both | All ages | Multiple myeloma | Metabolic risks | Number | 2021 | 1935.807 | 4872.289 | -851.07 |
| Deaths | China | Both | All ages | Multiple myeloma | Metabolic risks | Number | 2021 | 710.0606 | 1819.739 | -231.036 |
| Deaths | Germany | Both | All ages | Multiple myeloma | Metabolic risks | Number | 2021 | 445.9444 | 1187.536 | -174.584 |
| Deaths | Brazil | Both | All ages | Multiple myeloma | Metabolic risks | Number | 2021 | 413.7413 | 1063.985 | -174.158 |
| Deaths | United Kingdom of Great Britain and Northern Ireland | Both | All ages | Multiple myeloma | Metabolic risks | Number | 2021 | 394.3273 | 1002.504 | -170.755 |
| Deaths | India | Both | All ages | Multiple myeloma | Metabolic risks | Number | 2021 | 381.9831 | 988.0756 | -125.47 |
| Deaths | Russian Federation | Both | All ages | Multiple myeloma | Metabolic risks | Number | 2021 | 366.1005 | 914.9218 | -164.436 |
| Deaths | France | Both | All ages | Multiple myeloma | Metabolic risks | Number | 2021 | 343.1905 | 855.6682 | -134.512 |
| Deaths | Italy | Both | All ages | Multiple myeloma | Metabolic risks | Number | 2021 | 339.1256 | 890.0456 | -138.546 |
| Deaths | Spain | Both | All ages | Multiple myeloma | Metabolic risks | Number | 2021 | 233.839 | 600.3142 | -99.4201 |
| Deaths | Japan | Both | All ages | Multiple myeloma | Metabolic risks | Number | 2021 | 216.4546 | 576.6147 | -49.3194 |
| Deaths | Turkey | Both | All ages | Multiple myeloma | Metabolic risks | Number | 2021 | 208.3187 | 545.8471 | -100.843 |
| Deaths | Canada | Both | All ages | Multiple myeloma | Metabolic risks | Number | 2021 | 188.2591 | 479.9979 | -81.753 |
| Deaths | Mexico | Both | All ages | Multiple myeloma | Metabolic risks | Number | 2021 | 179.8768 | 453.0703 | -82.8681 |
| Deaths | Poland | Both | All ages | Multiple myeloma | Metabolic risks | Number | 2021 | 179.7156 | 459.1312 | -78.6575 |
| Deaths | Australia | Both | All ages | Multiple myeloma | Metabolic risks | Number | 2021 | 139.9711 | 351.4239 | -61.195 |
| Deaths | Pakistan | Both | All ages | Multiple myeloma | Metabolic risks | Number | 2021 | 104.1735 | 285.5943 | -32.948 |
| Deaths | South Africa | Both | All ages | Multiple myeloma | Metabolic risks | Number | 2021 | 101.3506 | 259.8502 | -47.6481 |
| Deaths | Argentina | Both | All ages | Multiple myeloma | Metabolic risks | Number | 2021 | 95.5731 | 241.0163 | -40.907 |
| Deaths | Netherlands | Both | All ages | Multiple myeloma | Metabolic risks | Number | 2021 | 91.17205 | 235.5948 | -33.6002 |
| Deaths | Colombia | Both | All ages | Multiple myeloma | Metabolic risks | Number | 2021 | 78.41487 | 207.7529 | -32.9454 |
| Deaths | Ukraine | Both | All ages | Multiple myeloma | Metabolic risks | Number | 2021 | 75.4686 | 192.7799 | -36.8882 |
| Deaths | Chile | Both | All ages | Multiple myeloma | Metabolic risks | Number | 2021 | 65.69155 | 165.7722 | -28.766 |
| Deaths | Islamic Republic of Iran | Both | All ages | Multiple myeloma | Metabolic risks | Number | 2021 | 61.76723 | 157.7657 | -27.8306 |
| Deaths | Greece | Both | All ages | Multiple myeloma | Metabolic risks | Number | 2021 | 59.34424 | 154.4743 | -23.1101 |
| Deaths | Portugal | Both | All ages | Multiple myeloma | Metabolic risks | Number | 2021 | 54.72483 | 140.8635 | -21.2984 |
| Deaths | Belgium | Both | All ages | Multiple myeloma | Metabolic risks | Number | 2021 | 54.04091 | 140.4992 | -21.2557 |
| Deaths | Czech Republic | Both | All ages | Multiple myeloma | Metabolic risks | Number | 2021 | 53.83685 | 141.8279 | -23.6895 |
| Deaths | Sweden | Both | All ages | Multiple myeloma | Metabolic risks | Number | 2021 | 49.08673 | 126.4233 | -17.6373 |
| Deaths | Romania | Both | All ages | Multiple myeloma | Metabolic risks | Number | 2021 | 48.72455 | 123.392 | -20.5838 |
| Deaths | Bolivarian Republic of Venezuela | Both | All ages | Multiple myeloma | Metabolic risks | Number | 2021 | 47.9783 | 123.9187 | -21.6699 |
| Deaths | Republic of Korea | Both | All ages | Multiple myeloma | Metabolic risks | Number | 2021 | 47.47598 | 125.6431 | -12.5178 |
| Deaths | Nigeria | Both | All ages | Multiple myeloma | Metabolic risks | Number | 2021 | 47.27176 | 146.2506 | -16.5555 |
| Deaths | Peru | Both | All ages | Multiple myeloma | Metabolic risks | Number | 2021 | 40.92747 | 110.7319 | -16.8737 |
| Deaths | Hungary | Both | All ages | Multiple myeloma | Metabolic risks | Number | 2021 | 37.75621 | 97.52445 | -17.3472 |
| Deaths | Algeria | Both | All ages | Multiple myeloma | Metabolic risks | Number | 2021 | 37.26645 | 98.98211 | -15.518 |
| Deaths | Indonesia | Both | All ages | Multiple myeloma | Metabolic risks | Number | 2021 | 35.32329 | 104.1319 | -12.0233 |
| Deaths | Taiwan (Province of China) | Both | All ages | Multiple myeloma | Metabolic risks | Number | 2021 | 35.09628 | 90.8612 | -11.9287 |
| Deaths | Austria | Both | All ages | Multiple myeloma | Metabolic risks | Number | 2021 | 34.5538 | 92.20648 | -13.5695 |
| Deaths | Switzerland | Both | All ages | Multiple myeloma | Metabolic risks | Number | 2021 | 34.50135 | 89.03179 | -11.5753 |
| Deaths | Thailand | Both | All ages | Multiple myeloma | Metabolic risks | Number | 2021 | 33.76792 | 108.8009 | -12.2685 |
| Deaths | Cuba | Both | All ages | Multiple myeloma | Metabolic risks | Number | 2021 | 33.49886 | 88.42639 | -13.964 |
| Deaths | Egypt | Both | All ages | Multiple myeloma | Metabolic risks | Number | 2021 | 33.0603 | 102.6789 | -16.154 |
| Deaths | Israel | Both | All ages | Multiple myeloma | Metabolic risks | Number | 2021 | 31.59088 | 79.6252 | -12.8996 |
| Deaths | Iraq | Both | All ages | Multiple myeloma | Metabolic risks | Number | 2021 | 30.87012 | 83.14693 | -14.0167 |
| Deaths | Finland | Both | All ages | Multiple myeloma | Metabolic risks | Number | 2021 | 30.10376 | 77.03237 | -12.3017 |
| Deaths | Serbia | Both | All ages | Multiple myeloma | Metabolic risks | Number | 2021 | 29.44583 | 72.99967 | -14.1275 |
| Deaths | Belarus | Both | All ages | Multiple myeloma | Metabolic risks | Number | 2021 | 28.43258 | 72.97058 | -13.6771 |
| Deaths | Saudi Arabia | Both | All ages | Multiple myeloma | Metabolic risks | Number | 2021 | 27.5399 | 70.55821 | -14.6381 |
| Deaths | New Zealand | Both | All ages | Multiple myeloma | Metabolic risks | Number | 2021 | 27.27965 | 67.4355 | -11.728 |
| Deaths | Denmark | Both | All ages | Multiple myeloma | Metabolic risks | Number | 2021 | 26.18927 | 67.58214 | -9.28333 |
| Deaths | Ecuador | Both | All ages | Multiple myeloma | Metabolic risks | Number | 2021 | 26.01379 | 65.45981 | -11.5786 |
| Deaths | Norway | Both | All ages | Multiple myeloma | Metabolic risks | Number | 2021 | 25.78548 | 66.81349 | -8.71649 |
| Deaths | Bangladesh | Both | All ages | Multiple myeloma | Metabolic risks | Number | 2021 | 25.73472 | 78.9735 | -5.91172 |
| Deaths | Slovakia | Both | All ages | Multiple myeloma | Metabolic risks | Number | 2021 | 25.24377 | 63.50592 | -12.1928 |
| Deaths | Morocco | Both | All ages | Multiple myeloma | Metabolic risks | Number | 2021 | 24.57778 | 67.30234 | -9.96522 |
| Deaths | Croatia | Both | All ages | Multiple myeloma | Metabolic risks | Number | 2021 | 22.45346 | 57.66755 | -9.56489 |
| Deaths | Puerto Rico | Both | All ages | Multiple myeloma | Metabolic risks | Number | 2021 | 20.11683 | 50.53533 | -8.98143 |
| Deaths | United Republic of Tanzania | Both | All ages | Multiple myeloma | Metabolic risks | Number | 2021 | 19.62607 | 54.03747 | -6.44854 |
| Deaths | Ireland | Both | All ages | Multiple myeloma | Metabolic risks | Number | 2021 | 19.09827 | 47.58833 | -7.48928 |
| Deaths | Lebanon | Both | All ages | Multiple myeloma | Metabolic risks | Number | 2021 | 18.14208 | 48.16009 | -8.22664 |
| Deaths | Tunisia | Both | All ages | Multiple myeloma | Metabolic risks | Number | 2021 | 18.07292 | 47.82482 | -7.99062 |
| Deaths | Zimbabwe | Both | All ages | Multiple myeloma | Metabolic risks | Number | 2021 | 17.30296 | 48.49524 | -6.53925 |
| Deaths | Bulgaria | Both | All ages | Multiple myeloma | Metabolic risks | Number | 2021 | 17.04666 | 45.80715 | -6.98432 |
| Deaths | Philippines | Both | All ages | Multiple myeloma | Metabolic risks | Number | 2021 | 16.92804 | 45.31889 | -5.14751 |
| Deaths | Uganda | Both | All ages | Multiple myeloma | Metabolic risks | Number | 2021 | 16.43136 | 46.9403 | -4.30732 |
| Deaths | Uruguay | Both | All ages | Multiple myeloma | Metabolic risks | Number | 2021 | 15.71662 | 41.56573 | -6.65667 |
| Deaths | Kenya | Both | All ages | Multiple myeloma | Metabolic risks | Number | 2021 | 15.53101 | 39.12858 | -5.09562 |
| Deaths | Malaysia | Both | All ages | Multiple myeloma | Metabolic risks | Number | 2021 | 15.36815 | 41.64495 | -5.63784 |
| Deaths | Lithuania | Both | All ages | Multiple myeloma | Metabolic risks | Number | 2021 | 14.98169 | 38.4842 | -6.07416 |
| Deaths | Costa Rica | Both | All ages | Multiple myeloma | Metabolic risks | Number | 2021 | 13.29126 | 34.89967 | -5.43293 |
| Deaths | Libya | Both | All ages | Multiple myeloma | Metabolic risks | Number | 2021 | 12.82211 | 34.17639 | -6.30708 |
| Deaths | Slovenia | Both | All ages | Multiple myeloma | Metabolic risks | Number | 2021 | 12.72426 | 33.52549 | -5.57613 |
| Deaths | Plurinational State of Bolivia | Both | All ages | Multiple myeloma | Metabolic risks | Number | 2021 | 12.67554 | 35.42421 | -5.28626 |
| Deaths | Sri Lanka | Both | All ages | Multiple myeloma | Metabolic risks | Number | 2021 | 12.17531 | 34.4802 | -3.69568 |
| Deaths | Dominican Republic | Both | All ages | Multiple myeloma | Metabolic risks | Number | 2021 | 11.78011 | 32.06172 | -4.81408 |
| Deaths | United Arab Emirates | Both | All ages | Multiple myeloma | Metabolic risks | Number | 2021 | 11.47837 | 28.68698 | -5.76039 |
| Deaths | Sudan | Both | All ages | Multiple myeloma | Metabolic risks | Number | 2021 | 11.03461 | 31.93499 | -4.63566 |
| Deaths | Kazakhstan | Both | All ages | Multiple myeloma | Metabolic risks | Number | 2021 | 10.70665 | 27.81935 | -4.87559 |
| Deaths | Latvia | Both | All ages | Multiple myeloma | Metabolic risks | Number | 2021 | 10.03815 | 25.61234 | -4.3067 |
| Deaths | Jamaica | Both | All ages | Multiple myeloma | Metabolic risks | Number | 2021 | 9.834141 | 25.71295 | -4.2039 |
| Deaths | Jordan | Both | All ages | Multiple myeloma | Metabolic risks | Number | 2021 | 9.799468 | 26.87516 | -4.91046 |
| Deaths | Panama | Both | All ages | Multiple myeloma | Metabolic risks | Number | 2021 | 9.741965 | 24.53463 | -4.23596 |
| Deaths | Zambia | Both | All ages | Multiple myeloma | Metabolic risks | Number | 2021 | 8.969453 | 27.54589 | -2.71353 |
| Deaths | Republic of C么te d'Ivoire | Both | All ages | Multiple myeloma | Metabolic risks | Number | 2021 | 8.169963 | 22.45153 | -2.71639 |
| Deaths | Syrian Arab Republic | Both | All ages | Multiple myeloma | Metabolic risks | Number | 2021 | 8.047908 | 21.81565 | -3.63919 |
| Deaths | Ethiopia | Both | All ages | Multiple myeloma | Metabolic risks | Number | 2021 | 7.827241 | 21.70937 | -1.6371 |
| Deaths | Estonia | Both | All ages | Multiple myeloma | Metabolic risks | Number | 2021 | 7.472247 | 19.3192 | -3.14366 |
| Deaths | Bosnia and Herzegovina | Both | All ages | Multiple myeloma | Metabolic risks | Number | 2021 | 7.023645 | 18.16054 | -3.02503 |
| Deaths | Georgia | Both | All ages | Multiple myeloma | Metabolic risks | Number | 2021 | 6.805236 | 17.47728 | -2.7542 |
| Deaths | Honduras | Both | All ages | Multiple myeloma | Metabolic risks | Number | 2021 | 6.678106 | 19.5432 | -2.79696 |
| Deaths | Paraguay | Both | All ages | Multiple myeloma | Metabolic risks | Number | 2021 | 6.495363 | 17.25723 | -2.75489 |
| Deaths | Democratic Republic of the Congo | Both | All ages | Multiple myeloma | Metabolic risks | Number | 2021 | 6.27647 | 19.12632 | -2.06983 |
| Deaths | Myanmar | Both | All ages | Multiple myeloma | Metabolic risks | Number | 2021 | 5.785673 | 17.31286 | -1.59136 |
| Deaths | Republic of Moldova | Both | All ages | Multiple myeloma | Metabolic risks | Number | 2021 | 5.685993 | 14.23872 | -2.58879 |
| Deaths | Mozambique | Both | All ages | Multiple myeloma | Metabolic risks | Number | 2021 | 5.452181 | 16.25281 | -1.37452 |
| Deaths | Uzbekistan | Both | All ages | Multiple myeloma | Metabolic risks | Number | 2021 | 5.417943 | 14.24455 | -2.31914 |
| Deaths | Trinidad and Tobago | Both | All ages | Multiple myeloma | Metabolic risks | Number | 2021 | 5.192367 | 13.81651 | -2.31585 |
| Deaths | Socialist Republic of Viet Nam | Both | All ages | Multiple myeloma | Metabolic risks | Number | 2021 | 5.168538 | 14.12262 | -1.06719 |
| Deaths | Guatemala | Both | All ages | Multiple myeloma | Metabolic risks | Number | 2021 | 5.092169 | 13.16273 | -2.2569 |
| Deaths | Nepal | Both | All ages | Multiple myeloma | Metabolic risks | Number | 2021 | 5.054108 | 16.00188 | -1.23066 |
| Deaths | Palestine | Both | All ages | Multiple myeloma | Metabolic risks | Number | 2021 | 4.622344 | 11.5882 | -2.04416 |
| Deaths | Yemen | Both | All ages | Multiple myeloma | Metabolic risks | Number | 2021 | 4.43214 | 12.85571 | -1.50767 |
| Deaths | Afghanistan | Both | All ages | Multiple myeloma | Metabolic risks | Number | 2021 | 4.28102 | 14.62651 | -1.53599 |
| Deaths | Cyprus | Both | All ages | Multiple myeloma | Metabolic risks | Number | 2021 | 4.108916 | 11.30435 | -1.54855 |
| Deaths | Turkmenistan | Both | All ages | Multiple myeloma | Metabolic risks | Number | 2021 | 3.815277 | 10.30796 | -1.55643 |
| Deaths | Armenia | Both | All ages | Multiple myeloma | Metabolic risks | Number | 2021 | 3.717796 | 9.441539 | -1.60939 |
| Deaths | El Salvador | Both | All ages | Multiple myeloma | Metabolic risks | Number | 2021 | 3.669612 | 9.336049 | -1.62468 |
| Deaths | Democratic People's Republic of Korea | Both | All ages | Multiple myeloma | Metabolic risks | Number | 2021 | 3.620882 | 11.98324 | -1.06237 |
| Deaths | Singapore | Both | All ages | Multiple myeloma | Metabolic risks | Number | 2021 | 3.459463 | 8.990837 | -1.07093 |
| Deaths | Kuwait | Both | All ages | Multiple myeloma | Metabolic risks | Number | 2021 | 3.37225 | 8.556189 | -1.77339 |
| Deaths | Haiti | Both | All ages | Multiple myeloma | Metabolic risks | Number | 2021 | 3.307675 | 10.82149 | -1.12882 |
| Deaths | North Macedonia | Both | All ages | Multiple myeloma | Metabolic risks | Number | 2021 | 3.306412 | 8.371657 | -1.43689 |
| Deaths | Madagascar | Both | All ages | Multiple myeloma | Metabolic risks | Number | 2021 | 3.076601 | 9.209703 | -0.82833 |
| Deaths | Angola | Both | All ages | Multiple myeloma | Metabolic risks | Number | 2021 | 3.007835 | 8.576996 | -0.80234 |
| Deaths | Rwanda | Both | All ages | Multiple myeloma | Metabolic risks | Number | 2021 | 2.782492 | 7.366562 | -0.60043 |
| Deaths | Azerbaijan | Both | All ages | Multiple myeloma | Metabolic risks | Number | 2021 | 2.738761 | 7.651029 | -1.17045 |
| Deaths | Oman | Both | All ages | Multiple myeloma | Metabolic risks | Number | 2021 | 2.720398 | 7.209866 | -1.25603 |
| Deaths | Luxembourg | Both | All ages | Multiple myeloma | Metabolic risks | Number | 2021 | 2.430655 | 6.479181 | -0.96553 |
| Deaths | Nicaragua | Both | All ages | Multiple myeloma | Metabolic risks | Number | 2021 | 2.311029 | 5.839863 | -1.02616 |
| Deaths | Mauritius | Both | All ages | Multiple myeloma | Metabolic risks | Number | 2021 | 2.283884 | 5.997581 | -0.83985 |
| Deaths | Albania | Both | All ages | Multiple myeloma | Metabolic risks | Number | 2021 | 2.172534 | 5.737403 | -0.92578 |
| Deaths | Botswana | Both | All ages | Multiple myeloma | Metabolic risks | Number | 2021 | 2.147367 | 5.61856 | -0.83627 |
| Deaths | Commonwealth of the Bahamas | Both | All ages | Multiple myeloma | Metabolic risks | Number | 2021 | 1.991306 | 5.017983 | -0.90164 |
| Deaths | Bahrain | Both | All ages | Multiple myeloma | Metabolic risks | Number | 2021 | 1.895778 | 5.037418 | -0.8316 |
| Deaths | Malawi | Both | All ages | Multiple myeloma | Metabolic risks | Number | 2021 | 1.692492 | 4.755859 | -0.45602 |
| Deaths | Lesotho | Both | All ages | Multiple myeloma | Metabolic risks | Number | 2021 | 1.562675 | 4.595308 | -0.68899 |
| Deaths | Iceland | Both | All ages | Multiple myeloma | Metabolic risks | Number | 2021 | 1.559731 | 4.040659 | -0.62593 |
| Deaths | Malta | Both | All ages | Multiple myeloma | Metabolic risks | Number | 2021 | 1.557816 | 3.903119 | -0.59972 |
| Deaths | Kyrgyzstan | Both | All ages | Multiple myeloma | Metabolic risks | Number | 2021 | 1.526828 | 3.959501 | -0.69079 |
| Deaths | Kingdom of Eswatini | Both | All ages | Multiple myeloma | Metabolic risks | Number | 2021 | 1.471959 | 4.244216 | -0.70044 |
| Deaths | Namibia | Both | All ages | Multiple myeloma | Metabolic risks | Number | 2021 | 1.45176 | 3.826806 | -0.57866 |
| Deaths | Cameroon | Both | All ages | Multiple myeloma | Metabolic risks | Number | 2021 | 1.365122 | 4.239618 | -0.48088 |
| Deaths | Qatar | Both | All ages | Multiple myeloma | Metabolic risks | Number | 2021 | 1.350059 | 3.8827 | -0.66486 |
| Deaths | Cambodia | Both | All ages | Multiple myeloma | Metabolic risks | Number | 2021 | 1.348282 | 4.094808 | -0.30749 |
| Deaths | Montenegro | Both | All ages | Multiple myeloma | Metabolic risks | Number | 2021 | 1.323238 | 3.301735 | -0.61374 |
| Deaths | Ghana | Both | All ages | Multiple myeloma | Metabolic risks | Number | 2021 | 1.227789 | 3.944166 | -0.33705 |
| Deaths | Congo | Both | All ages | Multiple myeloma | Metabolic risks | Number | 2021 | 1.164967 | 3.279713 | -0.43063 |
| Deaths | Tajikistan | Both | All ages | Multiple myeloma | Metabolic risks | Number | 2021 | 1.130507 | 3.129616 | -0.44134 |
| Deaths | Somalia | Both | All ages | Multiple myeloma | Metabolic risks | Number | 2021 | 1.127213 | 3.934884 | -0.30346 |
| Deaths | Barbados | Both | All ages | Multiple myeloma | Metabolic risks | Number | 2021 | 1.118015 | 2.974293 | -0.49623 |
| Deaths | Gabon | Both | All ages | Multiple myeloma | Metabolic risks | Number | 2021 | 1.107616 | 2.920703 | -0.49027 |
| Deaths | Burundi | Both | All ages | Multiple myeloma | Metabolic risks | Number | 2021 | 0.865722 | 2.573081 | -0.16228 |
| Deaths | South Sudan | Both | All ages | Multiple myeloma | Metabolic risks | Number | 2021 | 0.852489 | 2.475558 | -0.1267 |
| Deaths | Suriname | Both | All ages | Multiple myeloma | Metabolic risks | Number | 2021 | 0.776897 | 1.998261 | -0.27164 |
| Deaths | Fiji | Both | All ages | Multiple myeloma | Metabolic risks | Number | 2021 | 0.726473 | 1.858293 | -0.32843 |
| Deaths | Eritrea | Both | All ages | Multiple myeloma | Metabolic risks | Number | 2021 | 0.724556 | 2.185934 | -0.16307 |
| Deaths | Saint Lucia | Both | All ages | Multiple myeloma | Metabolic risks | Number | 2021 | 0.645616 | 1.673112 | -0.25163 |
| Deaths | Papua New Guinea | Both | All ages | Multiple myeloma | Metabolic risks | Number | 2021 | 0.612125 | 1.898428 | -0.20434 |
| Deaths | Lao People's Democratic Republic | Both | All ages | Multiple myeloma | Metabolic risks | Number | 2021 | 0.609588 | 2.176843 | -0.17439 |
| Deaths | Guyana | Both | All ages | Multiple myeloma | Metabolic risks | Number | 2021 | 0.594333 | 1.550692 | -0.24488 |
| Deaths | Mongolia | Both | All ages | Multiple myeloma | Metabolic risks | Number | 2021 | 0.56774 | 1.51886 | -0.23117 |
| Deaths | Guinea | Both | All ages | Multiple myeloma | Metabolic risks | Number | 2021 | 0.470868 | 1.314332 | -0.11087 |
| Deaths | United States Virgin Islands | Both | All ages | Multiple myeloma | Metabolic risks | Number | 2021 | 0.441395 | 1.22101 | -0.19233 |
| Deaths | Principality of Monaco | Both | All ages | Multiple myeloma | Metabolic risks | Number | 2021 | 0.430005 | 1.205806 | -0.1912 |
| Deaths | Brunei Darussalam | Both | All ages | Multiple myeloma | Metabolic risks | Number | 2021 | 0.412854 | 1.064 | -0.14195 |
| Deaths | Bermuda | Both | All ages | Multiple myeloma | Metabolic risks | Number | 2021 | 0.39941 | 1.039209 | -0.18757 |
| Deaths | Senegal | Both | All ages | Multiple myeloma | Metabolic risks | Number | 2021 | 0.397092 | 1.188355 | -0.11156 |
| Deaths | Equatorial Guinea | Both | All ages | Multiple myeloma | Metabolic risks | Number | 2021 | 0.372469 | 1.062613 | -0.14377 |
| Deaths | Bhutan | Both | All ages | Multiple myeloma | Metabolic risks | Number | 2021 | 0.366915 | 1.0691 | -0.12551 |
| Deaths | Comoros | Both | All ages | Multiple myeloma | Metabolic risks | Number | 2021 | 0.345846 | 0.984864 | -0.10092 |
| Deaths | Grenada | Both | All ages | Multiple myeloma | Metabolic risks | Number | 2021 | 0.342666 | 0.889327 | -0.13682 |
| Deaths | Central African Republic | Both | All ages | Multiple myeloma | Metabolic risks | Number | 2021 | 0.315786 | 0.932771 | -0.09015 |
| Deaths | Antigua and Barbuda | Both | All ages | Multiple myeloma | Metabolic risks | Number | 2021 | 0.272881 | 0.703861 | -0.11103 |
| Deaths | Belize | Both | All ages | Multiple myeloma | Metabolic risks | Number | 2021 | 0.256504 | 0.640905 | -0.11453 |
| Deaths | Dominica | Both | All ages | Multiple myeloma | Metabolic risks | Number | 2021 | 0.253248 | 0.699107 | -0.11148 |
| Deaths | Benin | Both | All ages | Multiple myeloma | Metabolic risks | Number | 2021 | 0.244905 | 0.747421 | -0.06726 |
| Deaths | Djibouti | Both | All ages | Multiple myeloma | Metabolic risks | Number | 2021 | 0.242996 | 0.694188 | -0.0497 |
| Deaths | Mauritania | Both | All ages | Multiple myeloma | Metabolic risks | Number | 2021 | 0.204648 | 0.642274 | -0.07054 |
| Deaths | Andorra | Both | All ages | Multiple myeloma | Metabolic risks | Number | 2021 | 0.19779 | 0.543961 | -0.07731 |
| Deaths | Burkina Faso | Both | All ages | Multiple myeloma | Metabolic risks | Number | 2021 | 0.191662 | 0.58838 | -0.02742 |
| Deaths | Tonga | Both | All ages | Multiple myeloma | Metabolic risks | Number | 2021 | 0.186508 | 0.461425 | -0.09255 |
| Deaths | Saint Vincent and the Grenadines | Both | All ages | Multiple myeloma | Metabolic risks | Number | 2021 | 0.180391 | 0.4711 | -0.06486 |
| Deaths | Saint Kitts and Nevis | Both | All ages | Multiple myeloma | Metabolic risks | Number | 2021 | 0.176974 | 0.462534 | -0.07876 |
| Deaths | Togo | Both | All ages | Multiple myeloma | Metabolic risks | Number | 2021 | 0.17296 | 0.540803 | -0.05006 |
| Deaths | Seychelles | Both | All ages | Multiple myeloma | Metabolic risks | Number | 2021 | 0.160791 | 0.408118 | -0.07165 |
| Deaths | Niger | Both | All ages | Multiple myeloma | Metabolic risks | Number | 2021 | 0.149956 | 0.497298 | -0.03619 |
| Deaths | Chad | Both | All ages | Multiple myeloma | Metabolic risks | Number | 2021 | 0.148269 | 0.456617 | -0.0353 |
| Deaths | Greenland | Both | All ages | Multiple myeloma | Metabolic risks | Number | 2021 | 0.134644 | 0.346178 | -0.05495 |
| Deaths | Liberia | Both | All ages | Multiple myeloma | Metabolic risks | Number | 2021 | 0.121619 | 0.406634 | -0.03803 |
| Deaths | Sierra Leone | Both | All ages | Multiple myeloma | Metabolic risks | Number | 2021 | 0.120494 | 0.355406 | -0.02842 |
| Deaths | Republic of San Marino | Both | All ages | Multiple myeloma | Metabolic risks | Number | 2021 | 0.112665 | 0.310061 | -0.0466 |
| Deaths | Samoa | Both | All ages | Multiple myeloma | Metabolic risks | Number | 2021 | 0.109133 | 0.315963 | -0.05059 |
| Deaths | Guam | Both | All ages | Multiple myeloma | Metabolic risks | Number | 2021 | 0.102256 | 0.26512 | -0.04627 |
| Deaths | Maldives | Both | All ages | Multiple myeloma | Metabolic risks | Number | 2021 | 0.090929 | 0.255219 | -0.032 |
| Deaths | Republic of the Gambia | Both | All ages | Multiple myeloma | Metabolic risks | Number | 2021 | 0.074432 | 0.210618 | -0.02393 |
| Deaths | Northern Mariana Islands | Both | All ages | Multiple myeloma | Metabolic risks | Number | 2021 | 0.073021 | 0.184518 | -0.03685 |
| Deaths | American Samoa | Both | All ages | Multiple myeloma | Metabolic risks | Number | 2021 | 0.069908 | 0.169288 | -0.03593 |
| Deaths | Solomon Islands | Both | All ages | Multiple myeloma | Metabolic risks | Number | 2021 | 0.068204 | 0.221518 | -0.02458 |
| Deaths | Vanuatu | Both | All ages | Multiple myeloma | Metabolic risks | Number | 2021 | 0.037301 | 0.105877 | -0.01408 |
| Deaths | Republic of Cabo Verde | Both | All ages | Multiple myeloma | Metabolic risks | Number | 2021 | 0.036503 | 0.111966 | -0.01217 |
| Deaths | Guinea-Bissau | Both | All ages | Multiple myeloma | Metabolic risks | Number | 2021 | 0.034555 | 0.105021 | -0.00975 |
| Deaths | Timor-Leste | Both | All ages | Multiple myeloma | Metabolic risks | Number | 2021 | 0.034051 | 0.113437 | -0.00604 |
| Deaths | Federated States of Micronesia | Both | All ages | Multiple myeloma | Metabolic risks | Number | 2021 | 0.03405 | 0.101236 | -0.01612 |
| Deaths | Marshall Islands | Both | All ages | Multiple myeloma | Metabolic risks | Number | 2021 | 0.013872 | 0.042492 | -0.00654 |
| Deaths | Cook Islands | Both | All ages | Multiple myeloma | Metabolic risks | Number | 2021 | 0.008566 | 0.022226 | -0.00428 |
| Deaths | Sao Tome and Principe | Both | All ages | Multiple myeloma | Metabolic risks | Number | 2021 | 0.008327 | 0.024988 | -0.00258 |
| Deaths | Kiribati | Both | All ages | Multiple myeloma | Metabolic risks | Number | 2021 | 0.005383 | 0.014574 | -0.00243 |
| Deaths | Republic of Nauru | Both | All ages | Multiple myeloma | Metabolic risks | Number | 2021 | 0.004255 | 0.013489 | -0.00201 |
| Deaths | Tuvalu | Both | All ages | Multiple myeloma | Metabolic risks | Number | 2021 | 0.00398 | 0.011177 | -0.0018 |
| Deaths | Republic of Palau | Both | All ages | Multiple myeloma | Metabolic risks | Number | 2021 | 0.001417 | 0.003754 | -0.00075 |
| Deaths | Republic of Niue | Both | All ages | Multiple myeloma | Metabolic risks | Number | 2021 | 0.001307 | 0.003451 | -0.0006 |
| Deaths | Tokelau | Both | All ages | Multiple myeloma | Metabolic risks | Number | 2021 | 0.000674 | 0.001843 | -0.00032 |
| Deaths | Mali | Both | All ages | Multiple myeloma | Metabolic risks | Number | 2021 | 0.000185 | 0.000539 | -0.00004 |

The ASMR of multiple myeloma by age and sex from 1990 to 2021 under the influence of metabolic factors

| measure | location | sex | age | cause | rei | metric | year | val | upper | lower |
| --- | --- | --- | --- | --- | --- | --- | --- | --- | --- | --- |
| Deaths | Commonwealth of the Bahamas | Both | Age-standardized | Multiple myeloma | Metabolic risks | Rate | 2021 | 0.485058 | 1.225163 | -0.21715 |
| Deaths | Principality of Monaco | Both | Age-standardized | Multiple myeloma | Metabolic risks | Rate | 2021 | 0.410829 | 1.148553 | -0.18574 |
| Deaths | United Arab Emirates | Both | Age-standardized | Multiple myeloma | Metabolic risks | Rate | 2021 | 0.353382 | 0.871326 | -0.17153 |
| Deaths | Jamaica | Both | Age-standardized | Multiple myeloma | Metabolic risks | Rate | 2021 | 0.320354 | 0.836478 | -0.13715 |
| Deaths | United States of America | Both | Age-standardized | Multiple myeloma | Metabolic risks | Rate | 2021 | 0.318042 | 0.800796 | -0.14076 |
| Deaths | New Zealand | Both | Age-standardized | Multiple myeloma | Metabolic risks | Rate | 2021 | 0.310556 | 0.765263 | -0.13433 |
| Deaths | Dominica | Both | Age-standardized | Multiple myeloma | Metabolic risks | Rate | 2021 | 0.299273 | 0.823969 | -0.13139 |
| Deaths | Lebanon | Both | Age-standardized | Multiple myeloma | Metabolic risks | Rate | 2021 | 0.296684 | 0.790241 | -0.13359 |
| Deaths | Grenada | Both | Age-standardized | Multiple myeloma | Metabolic risks | Rate | 2021 | 0.295308 | 0.770745 | -0.11393 |
| Deaths | Australia | Both | Age-standardized | Multiple myeloma | Metabolic risks | Rate | 2021 | 0.291879 | 0.732221 | -0.12867 |
| Deaths | Bermuda | Both | Age-standardized | Multiple myeloma | Metabolic risks | Rate | 2021 | 0.284841 | 0.734334 | -0.1342 |
| Deaths | Puerto Rico | Both | Age-standardized | Multiple myeloma | Metabolic risks | Rate | 2021 | 0.284427 | 0.710902 | -0.12963 |
| Deaths | United Kingdom of Great Britain and Northern Ireland | Both | Age-standardized | Multiple myeloma | Metabolic risks | Rate | 2021 | 0.281566 | 0.713127 | -0.12293 |
| Deaths | Uruguay | Both | Age-standardized | Multiple myeloma | Metabolic risks | Rate | 2021 | 0.280316 | 0.739381 | -0.11928 |
| Deaths | Estonia | Both | Age-standardized | Multiple myeloma | Metabolic risks | Rate | 2021 | 0.2794 | 0.714279 | -0.12006 |
| Deaths | Slovenia | Both | Age-standardized | Multiple myeloma | Metabolic risks | Rate | 2021 | 0.272583 | 0.71869 | -0.11938 |
| Deaths | Kingdom of Eswatini | Both | Age-standardized | Multiple myeloma | Metabolic risks | Rate | 2021 | 0.270484 | 0.773863 | -0.13005 |
| Deaths | Lithuania | Both | Age-standardized | Multiple myeloma | Metabolic risks | Rate | 2021 | 0.266809 | 0.681849 | -0.10739 |
| Deaths | Saint Lucia | Both | Age-standardized | Multiple myeloma | Metabolic risks | Rate | 2021 | 0.265688 | 0.689139 | -0.10272 |
| Deaths | Trinidad and Tobago | Both | Age-standardized | Multiple myeloma | Metabolic risks | Rate | 2021 | 0.262459 | 0.699033 | -0.11636 |
| Deaths | Saint Kitts and Nevis | Both | Age-standardized | Multiple myeloma | Metabolic risks | Rate | 2021 | 0.260951 | 0.685526 | -0.11007 |
| Deaths | Latvia | Both | Age-standardized | Multiple myeloma | Metabolic risks | Rate | 2021 | 0.259192 | 0.653275 | -0.11142 |
| Deaths | Slovakia | Both | Age-standardized | Multiple myeloma | Metabolic risks | Rate | 2021 | 0.257196 | 0.649686 | -0.12456 |
| Deaths | Iceland | Both | Age-standardized | Multiple myeloma | Metabolic risks | Rate | 2021 | 0.255329 | 0.659135 | -0.10211 |
| Deaths | Antigua and Barbuda | Both | Age-standardized | Multiple myeloma | Metabolic risks | Rate | 2021 | 0.254214 | 0.660332 | -0.10231 |
| Deaths | Libya | Both | Age-standardized | Multiple myeloma | Metabolic risks | Rate | 2021 | 0.254129 | 0.680218 | -0.12203 |
| Deaths | Chile | Both | Age-standardized | Multiple myeloma | Metabolic risks | Rate | 2021 | 0.252635 | 0.637622 | -0.11075 |
| Deaths | Zimbabwe | Both | Age-standardized | Multiple myeloma | Metabolic risks | Rate | 2021 | 0.248722 | 0.694583 | -0.09078 |
| Deaths | Canada | Both | Age-standardized | Multiple myeloma | Metabolic risks | Rate | 2021 | 0.245911 | 0.626181 | -0.10731 |
| Deaths | Israel | Both | Age-standardized | Multiple myeloma | Metabolic risks | Rate | 2021 | 0.244612 | 0.619309 | -0.10075 |
| Deaths | Netherlands | Both | Age-standardized | Multiple myeloma | Metabolic risks | Rate | 2021 | 0.243908 | 0.628183 | -0.0903 |
| Deaths | United States Virgin Islands | Both | Age-standardized | Multiple myeloma | Metabolic risks | Rate | 2021 | 0.242329 | 0.68195 | -0.10568 |
| Deaths | Poland | Both | Age-standardized | Multiple myeloma | Metabolic risks | Rate | 2021 | 0.241359 | 0.61838 | -0.10606 |
| Deaths | Croatia | Both | Age-standardized | Multiple myeloma | Metabolic risks | Rate | 2021 | 0.240604 | 0.616387 | -0.10383 |
| Deaths | Costa Rica | Both | Age-standardized | Multiple myeloma | Metabolic risks | Rate | 2021 | 0.240505 | 0.631242 | -0.09796 |
| Deaths | Czech Republic | Both | Age-standardized | Multiple myeloma | Metabolic risks | Rate | 2021 | 0.240415 | 0.633385 | -0.10509 |
| Deaths | Norway | Both | Age-standardized | Multiple myeloma | Metabolic risks | Rate | 2021 | 0.239975 | 0.618193 | -0.08179 |
| Deaths | Tonga | Both | Age-standardized | Multiple myeloma | Metabolic risks | Rate | 2021 | 0.232841 | 0.574558 | -0.11475 |
| Deaths | Ireland | Both | Age-standardized | Multiple myeloma | Metabolic risks | Rate | 2021 | 0.231762 | 0.576592 | -0.0914 |
| Deaths | Greece | Both | Age-standardized | Multiple myeloma | Metabolic risks | Rate | 2021 | 0.228502 | 0.58591 | -0.0898 |
| Deaths | Bahrain | Both | Age-standardized | Multiple myeloma | Metabolic risks | Rate | 2021 | 0.225771 | 0.600691 | -0.09726 |
| Deaths | Panama | Both | Age-standardized | Multiple myeloma | Metabolic risks | Rate | 2021 | 0.221376 | 0.557575 | -0.09629 |
| Deaths | Turkey | Both | Age-standardized | Multiple myeloma | Metabolic risks | Rate | 2021 | 0.220361 | 0.576707 | -0.10631 |
| Deaths | South Africa | Both | Age-standardized | Multiple myeloma | Metabolic risks | Rate | 2021 | 0.219601 | 0.567144 | -0.10272 |
| Deaths | Luxembourg | Both | Age-standardized | Multiple myeloma | Metabolic risks | Rate | 2021 | 0.219152 | 0.57942 | -0.08753 |
| Deaths | Spain | Both | Age-standardized | Multiple myeloma | Metabolic risks | Rate | 2021 | 0.218805 | 0.556391 | -0.09464 |
| Deaths | France | Both | Age-standardized | Multiple myeloma | Metabolic risks | Rate | 2021 | 0.217314 | 0.537527 | -0.08518 |
| Deaths | Finland | Both | Age-standardized | Multiple myeloma | Metabolic risks | Rate | 2021 | 0.216714 | 0.55115 | -0.08839 |
| Deaths | Belgium | Both | Age-standardized | Multiple myeloma | Metabolic risks | Rate | 2021 | 0.212887 | 0.552401 | -0.0851 |
| Deaths | Barbados | Both | Age-standardized | Multiple myeloma | Metabolic risks | Rate | 2021 | 0.212803 | 0.565398 | -0.09451 |
| Deaths | Germany | Both | Age-standardized | Multiple myeloma | Metabolic risks | Rate | 2021 | 0.212055 | 0.556987 | -0.08495 |
| Deaths | Italy | Both | Age-standardized | Multiple myeloma | Metabolic risks | Rate | 2021 | 0.21001 | 0.54392 | -0.08569 |
| Deaths | Portugal | Both | Age-standardized | Multiple myeloma | Metabolic risks | Rate | 2021 | 0.206444 | 0.527114 | -0.0821 |
| Deaths | Denmark | Both | Age-standardized | Multiple myeloma | Metabolic risks | Rate | 2021 | 0.203928 | 0.524527 | -0.07341 |
| Deaths | Sweden | Both | Age-standardized | Multiple myeloma | Metabolic risks | Rate | 2021 | 0.202721 | 0.521886 | -0.07266 |
| Deaths | Greenland | Both | Age-standardized | Multiple myeloma | Metabolic risks | Rate | 2021 | 0.196942 | 0.50933 | -0.07972 |
| Deaths | Cyprus | Both | Age-standardized | Multiple myeloma | Metabolic risks | Rate | 2021 | 0.196167 | 0.532962 | -0.0753 |
| Deaths | Hungary | Both | Age-standardized | Multiple myeloma | Metabolic risks | Rate | 2021 | 0.188666 | 0.484922 | -0.08763 |
| Deaths | Palestine | Both | Age-standardized | Multiple myeloma | Metabolic risks | Rate | 2021 | 0.188101 | 0.467123 | -0.08153 |
| Deaths | Austria | Both | Age-standardized | Multiple myeloma | Metabolic risks | Rate | 2021 | 0.175715 | 0.468325 | -0.06905 |
| Deaths | Belarus | Both | Age-standardized | Multiple myeloma | Metabolic risks | Rate | 2021 | 0.175072 | 0.448595 | -0.08427 |
| Deaths | Switzerland | Both | Age-standardized | Multiple myeloma | Metabolic risks | Rate | 2021 | 0.173439 | 0.443388 | -0.05838 |
| Deaths | Serbia | Both | Age-standardized | Multiple myeloma | Metabolic risks | Rate | 2021 | 0.172648 | 0.429563 | -0.08263 |
| Deaths | Cuba | Both | Age-standardized | Multiple myeloma | Metabolic risks | Rate | 2021 | 0.170595 | 0.451337 | -0.07132 |
| Deaths | Argentina | Both | Age-standardized | Multiple myeloma | Metabolic risks | Rate | 2021 | 0.169407 | 0.42785 | -0.07267 |
| Deaths | Brazil | Both | Age-standardized | Multiple myeloma | Metabolic risks | Rate | 2021 | 0.164811 | 0.424202 | -0.06923 |
| Deaths | Ecuador | Both | Age-standardized | Multiple myeloma | Metabolic risks | Rate | 2021 | 0.159063 | 0.401169 | -0.07061 |
| Deaths | Bolivarian Republic of Venezuela | Both | Age-standardized | Multiple myeloma | Metabolic risks | Rate | 2021 | 0.157405 | 0.407479 | -0.07057 |
| Deaths | Botswana | Both | Age-standardized | Multiple myeloma | Metabolic risks | Rate | 2021 | 0.151145 | 0.393756 | -0.05947 |
| Deaths | Qatar | Both | Age-standardized | Multiple myeloma | Metabolic risks | Rate | 2021 | 0.150315 | 0.417716 | -0.07524 |
| Deaths | Malta | Both | Age-standardized | Multiple myeloma | Metabolic risks | Rate | 2021 | 0.150254 | 0.374864 | -0.05822 |
| Deaths | Russian Federation | Both | Age-standardized | Multiple myeloma | Metabolic risks | Rate | 2021 | 0.150041 | 0.374916 | -0.06731 |
| Deaths | Lesotho | Both | Age-standardized | Multiple myeloma | Metabolic risks | Rate | 2021 | 0.146119 | 0.434214 | -0.06426 |
| Deaths | American Samoa | Both | Age-standardized | Multiple myeloma | Metabolic risks | Rate | 2021 | 0.145122 | 0.351619 | -0.0725 |
| Deaths | Saudi Arabia | Both | Age-standardized | Multiple myeloma | Metabolic risks | Rate | 2021 | 0.142337 | 0.362878 | -0.06935 |
| Deaths | Colombia | Both | Age-standardized | Multiple myeloma | Metabolic risks | Rate | 2021 | 0.142013 | 0.376083 | -0.05953 |
| Deaths | Oman | Both | Age-standardized | Multiple myeloma | Metabolic risks | Rate | 2021 | 0.140582 | 0.381271 | -0.06693 |
| Deaths | Republic of San Marino | Both | Age-standardized | Multiple myeloma | Metabolic risks | Rate | 2021 | 0.140391 | 0.381985 | -0.05861 |
| Deaths | Northern Mariana Islands | Both | Age-standardized | Multiple myeloma | Metabolic risks | Rate | 2021 | 0.140228 | 0.35678 | -0.06768 |
| Deaths | Mexico | Both | Age-standardized | Multiple myeloma | Metabolic risks | Rate | 2021 | 0.13926 | 0.350796 | -0.06396 |
| Deaths | Plurinational State of Bolivia | Both | Age-standardized | Multiple myeloma | Metabolic risks | Rate | 2021 | 0.138144 | 0.38727 | -0.05622 |
| Deaths | Tunisia | Both | Age-standardized | Multiple myeloma | Metabolic risks | Rate | 2021 | 0.136973 | 0.366382 | -0.06036 |
| Deaths | Jordan | Both | Age-standardized | Multiple myeloma | Metabolic risks | Rate | 2021 | 0.135598 | 0.370871 | -0.06757 |
| Deaths | Seychelles | Both | Age-standardized | Multiple myeloma | Metabolic risks | Rate | 2021 | 0.134242 | 0.341906 | -0.05919 |
| Deaths | Montenegro | Both | Age-standardized | Multiple myeloma | Metabolic risks | Rate | 2021 | 0.13267 | 0.33058 | -0.06145 |
| Deaths | Iraq | Both | Age-standardized | Multiple myeloma | Metabolic risks | Rate | 2021 | 0.132556 | 0.357216 | -0.05896 |
| Deaths | Romania | Both | Age-standardized | Multiple myeloma | Metabolic risks | Rate | 2021 | 0.130255 | 0.330253 | -0.0555 |
| Deaths | Andorra | Both | Age-standardized | Multiple myeloma | Metabolic risks | Rate | 2021 | 0.125256 | 0.345895 | -0.04933 |
| Deaths | Saint Vincent and the Grenadines | Both | Age-standardized | Multiple myeloma | Metabolic risks | Rate | 2021 | 0.124563 | 0.325994 | -0.04452 |
| Deaths | Zambia | Both | Age-standardized | Multiple myeloma | Metabolic risks | Rate | 2021 | 0.12269 | 0.37846 | -0.03674 |
| Deaths | Peru | Both | Age-standardized | Multiple myeloma | Metabolic risks | Rate | 2021 | 0.122678 | 0.332814 | -0.05042 |
| Deaths | Suriname | Both | Age-standardized | Multiple myeloma | Metabolic risks | Rate | 2021 | 0.120193 | 0.311148 | -0.04134 |
| Deaths | Mauritius | Both | Age-standardized | Multiple myeloma | Metabolic risks | Rate | 2021 | 0.119837 | 0.314739 | -0.04335 |
| Deaths | Georgia | Both | Age-standardized | Multiple myeloma | Metabolic risks | Rate | 2021 | 0.118577 | 0.303889 | -0.04804 |
| Deaths | Bulgaria | Both | Age-standardized | Multiple myeloma | Metabolic risks | Rate | 2021 | 0.117942 | 0.316627 | -0.04875 |
| Deaths | Dominican Republic | Both | Age-standardized | Multiple myeloma | Metabolic risks | Rate | 2021 | 0.117709 | 0.320002 | -0.04803 |
| Deaths | Kuwait | Both | Age-standardized | Multiple myeloma | Metabolic risks | Rate | 2021 | 0.11687 | 0.299209 | -0.06051 |
| Deaths | Paraguay | Both | Age-standardized | Multiple myeloma | Metabolic risks | Rate | 2021 | 0.112708 | 0.300482 | -0.04746 |
| Deaths | Brunei Darussalam | Both | Age-standardized | Multiple myeloma | Metabolic risks | Rate | 2021 | 0.112605 | 0.288671 | -0.034 |
| Deaths | Algeria | Both | Age-standardized | Multiple myeloma | Metabolic risks | Rate | 2021 | 0.112521 | 0.302625 | -0.04635 |
| Deaths | Uganda | Both | Age-standardized | Multiple myeloma | Metabolic risks | Rate | 2021 | 0.110472 | 0.31094 | -0.02914 |
| Deaths | Namibia | Both | Age-standardized | Multiple myeloma | Metabolic risks | Rate | 2021 | 0.109794 | 0.287072 | -0.04451 |
| Deaths | Bosnia and Herzegovina | Both | Age-standardized | Multiple myeloma | Metabolic risks | Rate | 2021 | 0.10927 | 0.281721 | -0.04715 |
| Deaths | Honduras | Both | Age-standardized | Multiple myeloma | Metabolic risks | Rate | 2021 | 0.104933 | 0.303539 | -0.04402 |
| Deaths | Gabon | Both | Age-standardized | Multiple myeloma | Metabolic risks | Rate | 2021 | 0.104096 | 0.278951 | -0.04483 |
| Deaths | North Macedonia | Both | Age-standardized | Multiple myeloma | Metabolic risks | Rate | 2021 | 0.099282 | 0.25098 | -0.04235 |
| Deaths | Fiji | Both | Age-standardized | Multiple myeloma | Metabolic risks | Rate | 2021 | 0.097842 | 0.246356 | -0.04345 |
| Deaths | Ukraine | Both | Age-standardized | Multiple myeloma | Metabolic risks | Rate | 2021 | 0.097817 | 0.249065 | -0.04751 |
| Deaths | Republic of Moldova | Both | Age-standardized | Multiple myeloma | Metabolic risks | Rate | 2021 | 0.093685 | 0.234491 | -0.04255 |
| Deaths | Guyana | Both | Age-standardized | Multiple myeloma | Metabolic risks | Rate | 2021 | 0.090131 | 0.234475 | -0.0366 |
| Deaths | Pakistan | Both | Age-standardized | Multiple myeloma | Metabolic risks | Rate | 2021 | 0.087033 | 0.237445 | -0.02652 |
| Deaths | Belize | Both | Age-standardized | Multiple myeloma | Metabolic risks | Rate | 2021 | 0.08584 | 0.215189 | -0.03823 |
| Deaths | Turkmenistan | Both | Age-standardized | Multiple myeloma | Metabolic risks | Rate | 2021 | 0.085246 | 0.23039 | -0.03455 |
| Deaths | Armenia | Both | Age-standardized | Multiple myeloma | Metabolic risks | Rate | 2021 | 0.083762 | 0.212428 | -0.03619 |
| Deaths | Taiwan (Province of China) | Both | Age-standardized | Multiple myeloma | Metabolic risks | Rate | 2021 | 0.083446 | 0.216042 | -0.02848 |
| Deaths | Islamic Republic of Iran | Both | Age-standardized | Multiple myeloma | Metabolic risks | Rate | 2021 | 0.081077 | 0.206654 | -0.03607 |
| Deaths | United Republic of Tanzania | Both | Age-standardized | Multiple myeloma | Metabolic risks | Rate | 2021 | 0.078349 | 0.213155 | -0.02474 |
| Deaths | Republic of C么te d'Ivoire | Both | Age-standardized | Multiple myeloma | Metabolic risks | Rate | 2021 | 0.075539 | 0.203178 | -0.02474 |
| Deaths | Equatorial Guinea | Both | Age-standardized | Multiple myeloma | Metabolic risks | Rate | 2021 | 0.0749 | 0.210887 | -0.02817 |
| Deaths | Samoa | Both | Age-standardized | Multiple myeloma | Metabolic risks | Rate | 2021 | 0.07473 | 0.214661 | -0.03428 |
| Deaths | Comoros | Both | Age-standardized | Multiple myeloma | Metabolic risks | Rate | 2021 | 0.071562 | 0.202304 | -0.0208 |
| Deaths | Morocco | Both | Age-standardized | Multiple myeloma | Metabolic risks | Rate | 2021 | 0.070814 | 0.191388 | -0.0285 |
| Deaths | Republic of Nauru | Both | Age-standardized | Multiple myeloma | Metabolic risks | Rate | 2021 | 0.069873 | 0.219602 | -0.03265 |
| Deaths | Kenya | Both | Age-standardized | Multiple myeloma | Metabolic risks | Rate | 2021 | 0.066925 | 0.16966 | -0.02147 |
| Deaths | Syrian Arab Republic | Both | Age-standardized | Multiple myeloma | Metabolic risks | Rate | 2021 | 0.06078 | 0.165446 | -0.02736 |
| Deaths | Republic of Niue | Both | Age-standardized | Multiple myeloma | Metabolic risks | Rate | 2021 | 0.060691 | 0.16018 | -0.02796 |
| Deaths | Bhutan | Both | Age-standardized | Multiple myeloma | Metabolic risks | Rate | 2021 | 0.060613 | 0.176508 | -0.02046 |
| Deaths | El Salvador | Both | Age-standardized | Multiple myeloma | Metabolic risks | Rate | 2021 | 0.060351 | 0.153646 | -0.02675 |
| Deaths | Nigeria | Both | Age-standardized | Multiple myeloma | Metabolic risks | Rate | 2021 | 0.056712 | 0.172433 | -0.01986 |
| Deaths | Kazakhstan | Both | Age-standardized | Multiple myeloma | Metabolic risks | Rate | 2021 | 0.055826 | 0.145596 | -0.02523 |
| Deaths | Sudan | Both | Age-standardized | Multiple myeloma | Metabolic risks | Rate | 2021 | 0.055789 | 0.159575 | -0.02315 |
| Deaths | Egypt | Both | Age-standardized | Multiple myeloma | Metabolic risks | Rate | 2021 | 0.053979 | 0.170813 | -0.02586 |
| Deaths | Malaysia | Both | Age-standardized | Multiple myeloma | Metabolic risks | Rate | 2021 | 0.053021 | 0.144386 | -0.01938 |
| Deaths | Japan | Both | Age-standardized | Multiple myeloma | Metabolic risks | Rate | 2021 | 0.051076 | 0.133617 | -0.01207 |
| Deaths | Republic of Korea | Both | Age-standardized | Multiple myeloma | Metabolic risks | Rate | 2021 | 0.049745 | 0.131221 | -0.01306 |
| Deaths | Guam | Both | Age-standardized | Multiple myeloma | Metabolic risks | Rate | 2021 | 0.048591 | 0.126094 | -0.02186 |
| Deaths | Albania | Both | Age-standardized | Multiple myeloma | Metabolic risks | Rate | 2021 | 0.04817 | 0.126828 | -0.02043 |
| Deaths | Mozambique | Both | Age-standardized | Multiple myeloma | Metabolic risks | Rate | 2021 | 0.047078 | 0.138912 | -0.01127 |
| Deaths | Nicaragua | Both | Age-standardized | Multiple myeloma | Metabolic risks | Rate | 2021 | 0.046872 | 0.118963 | -0.02072 |
| Deaths | Guatemala | Both | Age-standardized | Multiple myeloma | Metabolic risks | Rate | 2021 | 0.04552 | 0.117892 | -0.02011 |
| Deaths | Tokelau | Both | Age-standardized | Multiple myeloma | Metabolic risks | Rate | 2021 | 0.045208 | 0.123351 | -0.02134 |
| Deaths | Haiti | Both | Age-standardized | Multiple myeloma | Metabolic risks | Rate | 2021 | 0.044946 | 0.148375 | -0.01496 |
| Deaths | Rwanda | Both | Age-standardized | Multiple myeloma | Metabolic risks | Rate | 2021 | 0.044109 | 0.117711 | -0.0094 |
| Deaths | Sri Lanka | Both | Age-standardized | Multiple myeloma | Metabolic risks | Rate | 2021 | 0.043899 | 0.124048 | -0.0133 |
| Deaths | Federated States of Micronesia | Both | Age-standardized | Multiple myeloma | Metabolic risks | Rate | 2021 | 0.043794 | 0.127195 | -0.02025 |
| Deaths | Afghanistan | Both | Age-standardized | Multiple myeloma | Metabolic risks | Rate | 2021 | 0.043112 | 0.140573 | -0.01659 |
| Deaths | Congo | Both | Age-standardized | Multiple myeloma | Metabolic risks | Rate | 2021 | 0.040962 | 0.115122 | -0.01472 |
| Deaths | Singapore | Both | Age-standardized | Multiple myeloma | Metabolic risks | Rate | 2021 | 0.04038 | 0.104575 | -0.01238 |
| Deaths | Marshall Islands | Both | Age-standardized | Multiple myeloma | Metabolic risks | Rate | 2021 | 0.038663 | 0.119675 | -0.0186 |
| Deaths | Djibouti | Both | Age-standardized | Multiple myeloma | Metabolic risks | Rate | 2021 | 0.037607 | 0.10886 | -0.0069 |
| Deaths | Tuvalu | Both | Age-standardized | Multiple myeloma | Metabolic risks | Rate | 2021 | 0.037399 | 0.104466 | -0.01653 |
| Deaths | China | Both | Age-standardized | Multiple myeloma | Metabolic risks | Rate | 2021 | 0.033188 | 0.085436 | -0.01063 |
| Deaths | Cook Islands | Both | Age-standardized | Multiple myeloma | Metabolic risks | Rate | 2021 | 0.033186 | 0.085859 | -0.01649 |
| Deaths | India | Both | Age-standardized | Multiple myeloma | Metabolic risks | Rate | 2021 | 0.03231 | 0.083238 | -0.0106 |
| Deaths | Yemen | Both | Age-standardized | Multiple myeloma | Metabolic risks | Rate | 2021 | 0.031641 | 0.091015 | -0.01094 |
| Deaths | Thailand | Both | Age-standardized | Multiple myeloma | Metabolic risks | Rate | 2021 | 0.030581 | 0.09826 | -0.01106 |
| Deaths | Kyrgyzstan | Both | Age-standardized | Multiple myeloma | Metabolic risks | Rate | 2021 | 0.028735 | 0.074742 | -0.013 |
| Deaths | Madagascar | Both | Age-standardized | Multiple myeloma | Metabolic risks | Rate | 2021 | 0.028633 | 0.084837 | -0.00751 |
| Deaths | Eritrea | Both | Age-standardized | Multiple myeloma | Metabolic risks | Rate | 2021 | 0.025955 | 0.077544 | -0.00539 |
| Deaths | Maldives | Both | Age-standardized | Multiple myeloma | Metabolic risks | Rate | 2021 | 0.024801 | 0.065228 | -0.00851 |
| Deaths | Angola | Both | Age-standardized | Multiple myeloma | Metabolic risks | Rate | 2021 | 0.024573 | 0.070817 | -0.00634 |
| Deaths | Azerbaijan | Both | Age-standardized | Multiple myeloma | Metabolic risks | Rate | 2021 | 0.024288 | 0.066396 | -0.0102 |
| Deaths | Malawi | Both | Age-standardized | Multiple myeloma | Metabolic risks | Rate | 2021 | 0.022963 | 0.063907 | -0.00597 |
| Deaths | Mongolia | Both | Age-standardized | Multiple myeloma | Metabolic risks | Rate | 2021 | 0.022036 | 0.057588 | -0.00888 |
| Deaths | South Sudan | Both | Age-standardized | Multiple myeloma | Metabolic risks | Rate | 2021 | 0.021833 | 0.062357 | -0.00322 |
| Deaths | Nepal | Both | Age-standardized | Multiple myeloma | Metabolic risks | Rate | 2021 | 0.02101 | 0.065996 | -0.00497 |
| Deaths | Vanuatu | Both | Age-standardized | Multiple myeloma | Metabolic risks | Rate | 2021 | 0.020614 | 0.059931 | -0.00744 |
| Deaths | Philippines | Both | Age-standardized | Multiple myeloma | Metabolic risks | Rate | 2021 | 0.019743 | 0.052534 | -0.00605 |
| Deaths | Uzbekistan | Both | Age-standardized | Multiple myeloma | Metabolic risks | Rate | 2021 | 0.018865 | 0.049873 | -0.00814 |
| Deaths | Solomon Islands | Both | Age-standardized | Multiple myeloma | Metabolic risks | Rate | 2021 | 0.0185 | 0.060149 | -0.00637 |
| Deaths | Bangladesh | Both | Age-standardized | Multiple myeloma | Metabolic risks | Rate | 2021 | 0.018407 | 0.055812 | -0.00414 |
| Deaths | Ethiopia | Both | Age-standardized | Multiple myeloma | Metabolic risks | Rate | 2021 | 0.018198 | 0.050709 | -0.00372 |
| Deaths | Burundi | Both | Age-standardized | Multiple myeloma | Metabolic risks | Rate | 2021 | 0.018158 | 0.054195 | -0.00337 |
| Deaths | Democratic Republic of the Congo | Both | Age-standardized | Multiple myeloma | Metabolic risks | Rate | 2021 | 0.017572 | 0.051745 | -0.00584 |
| Deaths | Tajikistan | Both | Age-standardized | Multiple myeloma | Metabolic risks | Rate | 2021 | 0.01733 | 0.048662 | -0.00673 |
| Deaths | Somalia | Both | Age-standardized | Multiple myeloma | Metabolic risks | Rate | 2021 | 0.017316 | 0.059125 | -0.00435 |
| Deaths | Indonesia | Both | Age-standardized | Multiple myeloma | Metabolic risks | Rate | 2021 | 0.013698 | 0.041119 | -0.00479 |
| Deaths | Central African Republic | Both | Age-standardized | Multiple myeloma | Metabolic risks | Rate | 2021 | 0.013204 | 0.040247 | -0.00363 |
| Deaths | Lao People's Democratic Republic | Both | Age-standardized | Multiple myeloma | Metabolic risks | Rate | 2021 | 0.012588 | 0.044107 | -0.00364 |
| Deaths | Cameroon | Both | Age-standardized | Multiple myeloma | Metabolic risks | Rate | 2021 | 0.011404 | 0.034668 | -0.00393 |
| Deaths | Democratic People's Republic of Korea | Both | Age-standardized | Multiple myeloma | Metabolic risks | Rate | 2021 | 0.011342 | 0.038319 | -0.00336 |
| Deaths | Myanmar | Both | Age-standardized | Multiple myeloma | Metabolic risks | Rate | 2021 | 0.011191 | 0.033869 | -0.00304 |
| Deaths | Papua New Guinea | Both | Age-standardized | Multiple myeloma | Metabolic risks | Rate | 2021 | 0.010741 | 0.033167 | -0.00301 |
| Deaths | Cambodia | Both | Age-standardized | Multiple myeloma | Metabolic risks | Rate | 2021 | 0.010258 | 0.031823 | -0.00226 |
| Deaths | Mauritania | Both | Age-standardized | Multiple myeloma | Metabolic risks | Rate | 2021 | 0.010036 | 0.03136 | -0.00341 |
| Deaths | Guinea | Both | Age-standardized | Multiple myeloma | Metabolic risks | Rate | 2021 | 0.008572 | 0.02374 | -0.00198 |
| Deaths | Republic of Cabo Verde | Both | Age-standardized | Multiple myeloma | Metabolic risks | Rate | 2021 | 0.008477 | 0.026014 | -0.00277 |
| Deaths | Sao Tome and Principe | Both | Age-standardized | Multiple myeloma | Metabolic risks | Rate | 2021 | 0.007805 | 0.023761 | -0.00233 |
| Deaths | Republic of the Gambia | Both | Age-standardized | Multiple myeloma | Metabolic risks | Rate | 2021 | 0.007769 | 0.02178 | -0.00243 |
| Deaths | Ghana | Both | Age-standardized | Multiple myeloma | Metabolic risks | Rate | 2021 | 0.007615 | 0.024829 | -0.00204 |
| Deaths | Kiribati | Both | Age-standardized | Multiple myeloma | Metabolic risks | Rate | 2021 | 0.007148 | 0.019921 | -0.00307 |
| Deaths | Republic of Palau | Both | Age-standardized | Multiple myeloma | Metabolic risks | Rate | 2021 | 0.006093 | 0.016124 | -0.00314 |
| Deaths | Liberia | Both | Age-standardized | Multiple myeloma | Metabolic risks | Rate | 2021 | 0.005839 | 0.019553 | -0.00174 |
| Deaths | Senegal | Both | Age-standardized | Multiple myeloma | Metabolic risks | Rate | 2021 | 0.005254 | 0.01571 | -0.00144 |
| Deaths | Socialist Republic of Viet Nam | Both | Age-standardized | Multiple myeloma | Metabolic risks | Rate | 2021 | 0.005139 | 0.013991 | -0.00103 |
| Deaths | Benin | Both | Age-standardized | Multiple myeloma | Metabolic risks | Rate | 2021 | 0.004818 | 0.0148 | -0.00133 |
| Deaths | Guinea-Bissau | Both | Age-standardized | Multiple myeloma | Metabolic risks | Rate | 2021 | 0.004795 | 0.014936 | -0.00135 |
| Deaths | Togo | Both | Age-standardized | Multiple myeloma | Metabolic risks | Rate | 2021 | 0.004792 | 0.014863 | -0.00131 |
| Deaths | Timor-Leste | Both | Age-standardized | Multiple myeloma | Metabolic risks | Rate | 2021 | 0.003885 | 0.01297 | -0.00068 |
| Deaths | Sierra Leone | Both | Age-standardized | Multiple myeloma | Metabolic risks | Rate | 2021 | 0.003299 | 0.009733 | -0.00079 |
| Deaths | Chad | Both | Age-standardized | Multiple myeloma | Metabolic risks | Rate | 2021 | 0.002658 | 0.008289 | -0.0006 |
| Deaths | Burkina Faso | Both | Age-standardized | Multiple myeloma | Metabolic risks | Rate | 2021 | 0.002055 | 0.006379 | -0.00027 |
| Deaths | Niger | Both | Age-standardized | Multiple myeloma | Metabolic risks | Rate | 2021 | 0.001852 | 0.006015 | -0.00043 |
| Deaths | Mali | Both | Age-standardized | Multiple myeloma | Metabolic risks | Rate | 2021 | 2.17E-06 | 6.27E-06 | -5.10E-07 |

The ASDR of multiple myeloma by age and sex from 1990 to 2021 under the influence of metabolic factors

| measure | location | sex | age | cause | rei | metric | year | val | upper | lower |
| --- | --- | --- | --- | --- | --- | --- | --- | --- | --- | --- |
| DALYs (Disability-Adjusted Life Years) | Commonwealth of the Bahamas | Both | Age-standardized | Multiple myeloma | Metabolic risks | Rate | 2021 | 12.70935 | 32.18757 | -5.78294 |
| DALYs (Disability-Adjusted Life Years) | Principality of Monaco | Both | Age-standardized | Multiple myeloma | Metabolic risks | Rate | 2021 | 8.963703 | 26.1374 | -4.15591 |
| DALYs (Disability-Adjusted Life Years) | Jamaica | Both | Age-standardized | Multiple myeloma | Metabolic risks | Rate | 2021 | 8.164621 | 21.07267 | -3.5651 |
| DALYs (Disability-Adjusted Life Years) | United Arab Emirates | Both | Age-standardized | Multiple myeloma | Metabolic risks | Rate | 2021 | 7.802345 | 18.91567 | -3.92506 |
| DALYs (Disability-Adjusted Life Years) | Grenada | Both | Age-standardized | Multiple myeloma | Metabolic risks | Rate | 2021 | 7.562699 | 19.55534 | -3.15584 |
| DALYs (Disability-Adjusted Life Years) | Puerto Rico | Both | Age-standardized | Multiple myeloma | Metabolic risks | Rate | 2021 | 7.348631 | 18.23986 | -3.44523 |
| DALYs (Disability-Adjusted Life Years) | Dominica | Both | Age-standardized | Multiple myeloma | Metabolic risks | Rate | 2021 | 7.344105 | 19.97268 | -3.24631 |
| DALYs (Disability-Adjusted Life Years) | Saint Lucia | Both | Age-standardized | Multiple myeloma | Metabolic risks | Rate | 2021 | 7.007336 | 17.96849 | -2.84987 |
| DALYs (Disability-Adjusted Life Years) | Trinidad and Tobago | Both | Age-standardized | Multiple myeloma | Metabolic risks | Rate | 2021 | 6.989221 | 18.48086 | -3.17279 |
| DALYs (Disability-Adjusted Life Years) | Estonia | Both | Age-standardized | Multiple myeloma | Metabolic risks | Rate | 2021 | 6.984372 | 18.02812 | -3.07163 |
| DALYs (Disability-Adjusted Life Years) | New Zealand | Both | Age-standardized | Multiple myeloma | Metabolic risks | Rate | 2021 | 6.95773 | 17.03201 | -3.14921 |
| DALYs (Disability-Adjusted Life Years) | Kingdom of Eswatini | Both | Age-standardized | Multiple myeloma | Metabolic risks | Rate | 2021 | 6.944892 | 19.99924 | -3.30633 |
| DALYs (Disability-Adjusted Life Years) | Lithuania | Both | Age-standardized | Multiple myeloma | Metabolic risks | Rate | 2021 | 6.902429 | 17.52218 | -2.76238 |
| DALYs (Disability-Adjusted Life Years) | Bermuda | Both | Age-standardized | Multiple myeloma | Metabolic risks | Rate | 2021 | 6.880059 | 17.41888 | -3.25592 |
| DALYs (Disability-Adjusted Life Years) | Lebanon | Both | Age-standardized | Multiple myeloma | Metabolic risks | Rate | 2021 | 6.845941 | 18.111 | -3.06558 |
| DALYs (Disability-Adjusted Life Years) | United States of America | Both | Age-standardized | Multiple myeloma | Metabolic risks | Rate | 2021 | 6.758323 | 16.74119 | -3.09733 |
| DALYs (Disability-Adjusted Life Years) | Latvia | Both | Age-standardized | Multiple myeloma | Metabolic risks | Rate | 2021 | 6.704736 | 16.73266 | -2.91263 |
| DALYs (Disability-Adjusted Life Years) | Uruguay | Both | Age-standardized | Multiple myeloma | Metabolic risks | Rate | 2021 | 6.659114 | 17.49948 | -2.86746 |
| DALYs (Disability-Adjusted Life Years) | Zimbabwe | Both | Age-standardized | Multiple myeloma | Metabolic risks | Rate | 2021 | 6.568837 | 18.27202 | -2.48269 |
| DALYs (Disability-Adjusted Life Years) | Costa Rica | Both | Age-standardized | Multiple myeloma | Metabolic risks | Rate | 2021 | 6.442923 | 16.60565 | -2.69445 |
| DALYs (Disability-Adjusted Life Years) | Australia | Both | Age-standardized | Multiple myeloma | Metabolic risks | Rate | 2021 | 6.340318 | 15.94225 | -2.92981 |
| DALYs (Disability-Adjusted Life Years) | Libya | Both | Age-standardized | Multiple myeloma | Metabolic risks | Rate | 2021 | 6.323681 | 16.81558 | -3.1282 |
| DALYs (Disability-Adjusted Life Years) | United States Virgin Islands | Both | Age-standardized | Multiple myeloma | Metabolic risks | Rate | 2021 | 6.292431 | 17.84014 | -2.78705 |
| DALYs (Disability-Adjusted Life Years) | Saint Kitts and Nevis | Both | Age-standardized | Multiple myeloma | Metabolic risks | Rate | 2021 | 6.270447 | 16.33816 | -2.79207 |
| DALYs (Disability-Adjusted Life Years) | Antigua and Barbuda | Both | Age-standardized | Multiple myeloma | Metabolic risks | Rate | 2021 | 6.256985 | 16.06401 | -2.62253 |
| DALYs (Disability-Adjusted Life Years) | Tonga | Both | Age-standardized | Multiple myeloma | Metabolic risks | Rate | 2021 | 6.202549 | 15.40029 | -3.16838 |
| DALYs (Disability-Adjusted Life Years) | Slovakia | Both | Age-standardized | Multiple myeloma | Metabolic risks | Rate | 2021 | 6.141759 | 15.31821 | -2.97722 |
| DALYs (Disability-Adjusted Life Years) | United Kingdom of Great Britain and Northern Ireland | Both | Age-standardized | Multiple myeloma | Metabolic risks | Rate | 2021 | 6.01024 | 15.03197 | -2.70433 |
| DALYs (Disability-Adjusted Life Years) | Chile | Both | Age-standardized | Multiple myeloma | Metabolic risks | Rate | 2021 | 5.988135 | 15.07909 | -2.6945 |
| DALYs (Disability-Adjusted Life Years) | Slovenia | Both | Age-standardized | Multiple myeloma | Metabolic risks | Rate | 2021 | 5.982347 | 15.6547 | -2.61562 |
| DALYs (Disability-Adjusted Life Years) | South Africa | Both | Age-standardized | Multiple myeloma | Metabolic risks | Rate | 2021 | 5.693889 | 14.5501 | -2.69393 |
| DALYs (Disability-Adjusted Life Years) | Croatia | Both | Age-standardized | Multiple myeloma | Metabolic risks | Rate | 2021 | 5.509709 | 14.2077 | -2.41145 |
| DALYs (Disability-Adjusted Life Years) | Turkey | Both | Age-standardized | Multiple myeloma | Metabolic risks | Rate | 2021 | 5.506736 | 14.37548 | -2.6983 |
| DALYs (Disability-Adjusted Life Years) | Czech Republic | Both | Age-standardized | Multiple myeloma | Metabolic risks | Rate | 2021 | 5.504197 | 14.43997 | -2.40991 |
| DALYs (Disability-Adjusted Life Years) | Panama | Both | Age-standardized | Multiple myeloma | Metabolic risks | Rate | 2021 | 5.477702 | 13.77793 | -2.36959 |
| DALYs (Disability-Adjusted Life Years) | Iceland | Both | Age-standardized | Multiple myeloma | Metabolic risks | Rate | 2021 | 5.456812 | 13.92831 | -2.19788 |
| DALYs (Disability-Adjusted Life Years) | Poland | Both | Age-standardized | Multiple myeloma | Metabolic risks | Rate | 2021 | 5.361317 | 13.74161 | -2.40112 |
| DALYs (Disability-Adjusted Life Years) | Barbados | Both | Age-standardized | Multiple myeloma | Metabolic risks | Rate | 2021 | 5.312213 | 13.81233 | -2.43065 |
| DALYs (Disability-Adjusted Life Years) | Bahrain | Both | Age-standardized | Multiple myeloma | Metabolic risks | Rate | 2021 | 5.267209 | 13.75293 | -2.2916 |
| DALYs (Disability-Adjusted Life Years) | Canada | Both | Age-standardized | Multiple myeloma | Metabolic risks | Rate | 2021 | 5.215846 | 13.41478 | -2.36463 |
| DALYs (Disability-Adjusted Life Years) | Netherlands | Both | Age-standardized | Multiple myeloma | Metabolic risks | Rate | 2021 | 5.164045 | 13.22676 | -1.98323 |
| DALYs (Disability-Adjusted Life Years) | Israel | Both | Age-standardized | Multiple myeloma | Metabolic risks | Rate | 2021 | 5.115621 | 13.00643 | -2.20992 |
| DALYs (Disability-Adjusted Life Years) | Norway | Both | Age-standardized | Multiple myeloma | Metabolic risks | Rate | 2021 | 4.974319 | 12.84521 | -1.7463 |
| DALYs (Disability-Adjusted Life Years) | Greece | Both | Age-standardized | Multiple myeloma | Metabolic risks | Rate | 2021 | 4.961885 | 12.71013 | -2.03552 |
| DALYs (Disability-Adjusted Life Years) | Ireland | Both | Age-standardized | Multiple myeloma | Metabolic risks | Rate | 2021 | 4.910952 | 11.97779 | -2.01802 |
| DALYs (Disability-Adjusted Life Years) | Greenland | Both | Age-standardized | Multiple myeloma | Metabolic risks | Rate | 2021 | 4.706379 | 12.13072 | -1.90699 |
| DALYs (Disability-Adjusted Life Years) | Palestine | Both | Age-standardized | Multiple myeloma | Metabolic risks | Rate | 2021 | 4.647868 | 11.63841 | -2.06058 |
| DALYs (Disability-Adjusted Life Years) | Belarus | Both | Age-standardized | Multiple myeloma | Metabolic risks | Rate | 2021 | 4.6467 | 11.76883 | -2.26136 |
| DALYs (Disability-Adjusted Life Years) | Spain | Both | Age-standardized | Multiple myeloma | Metabolic risks | Rate | 2021 | 4.643906 | 11.57757 | -2.08303 |
| DALYs (Disability-Adjusted Life Years) | Finland | Both | Age-standardized | Multiple myeloma | Metabolic risks | Rate | 2021 | 4.602685 | 11.7213 | -1.88171 |
| DALYs (Disability-Adjusted Life Years) | Germany | Both | Age-standardized | Multiple myeloma | Metabolic risks | Rate | 2021 | 4.573971 | 11.81727 | -1.87697 |
| DALYs (Disability-Adjusted Life Years) | Luxembourg | Both | Age-standardized | Multiple myeloma | Metabolic risks | Rate | 2021 | 4.551376 | 11.70549 | -1.82851 |
| DALYs (Disability-Adjusted Life Years) | Hungary | Both | Age-standardized | Multiple myeloma | Metabolic risks | Rate | 2021 | 4.509983 | 11.48105 | -2.10806 |
| DALYs (Disability-Adjusted Life Years) | France | Both | Age-standardized | Multiple myeloma | Metabolic risks | Rate | 2021 | 4.487314 | 11.02349 | -1.77739 |
| DALYs (Disability-Adjusted Life Years) | Cuba | Both | Age-standardized | Multiple myeloma | Metabolic risks | Rate | 2021 | 4.46246 | 11.77028 | -1.96875 |
| DALYs (Disability-Adjusted Life Years) | Belgium | Both | Age-standardized | Multiple myeloma | Metabolic risks | Rate | 2021 | 4.462243 | 11.51007 | -1.80541 |
| DALYs (Disability-Adjusted Life Years) | Italy | Both | Age-standardized | Multiple myeloma | Metabolic risks | Rate | 2021 | 4.428536 | 11.35685 | -1.82569 |
| DALYs (Disability-Adjusted Life Years) | Portugal | Both | Age-standardized | Multiple myeloma | Metabolic risks | Rate | 2021 | 4.402335 | 11.23299 | -1.81766 |
| DALYs (Disability-Adjusted Life Years) | Denmark | Both | Age-standardized | Multiple myeloma | Metabolic risks | Rate | 2021 | 4.213659 | 10.74741 | -1.58764 |
| DALYs (Disability-Adjusted Life Years) | Bolivarian Republic of Venezuela | Both | Age-standardized | Multiple myeloma | Metabolic risks | Rate | 2021 | 4.159531 | 10.76705 | -1.88995 |
| DALYs (Disability-Adjusted Life Years) | Cyprus | Both | Age-standardized | Multiple myeloma | Metabolic risks | Rate | 2021 | 4.132778 | 10.96392 | -1.5804 |
| DALYs (Disability-Adjusted Life Years) | Argentina | Both | Age-standardized | Multiple myeloma | Metabolic risks | Rate | 2021 | 4.102808 | 10.32531 | -1.79768 |
| DALYs (Disability-Adjusted Life Years) | Brazil | Both | Age-standardized | Multiple myeloma | Metabolic risks | Rate | 2021 | 4.101274 | 10.48425 | -1.75133 |
| DALYs (Disability-Adjusted Life Years) | Serbia | Both | Age-standardized | Multiple myeloma | Metabolic risks | Rate | 2021 | 4.08823 | 10.10294 | -1.99516 |
| DALYs (Disability-Adjusted Life Years) | Ecuador | Both | Age-standardized | Multiple myeloma | Metabolic risks | Rate | 2021 | 4.078088 | 10.06085 | -1.83763 |
| DALYs (Disability-Adjusted Life Years) | Sweden | Both | Age-standardized | Multiple myeloma | Metabolic risks | Rate | 2021 | 4.008607 | 10.22778 | -1.4589 |
| DALYs (Disability-Adjusted Life Years) | American Samoa | Both | Age-standardized | Multiple myeloma | Metabolic risks | Rate | 2021 | 3.930937 | 9.570887 | -2.04219 |
| DALYs (Disability-Adjusted Life Years) | Russian Federation | Both | Age-standardized | Multiple myeloma | Metabolic risks | Rate | 2021 | 3.88914 | 9.619284 | -1.75467 |
| DALYs (Disability-Adjusted Life Years) | Mexico | Both | Age-standardized | Multiple myeloma | Metabolic risks | Rate | 2021 | 3.831457 | 9.599376 | -1.78326 |
| DALYs (Disability-Adjusted Life Years) | Lesotho | Both | Age-standardized | Multiple myeloma | Metabolic risks | Rate | 2021 | 3.816298 | 11.16209 | -1.67981 |
| DALYs (Disability-Adjusted Life Years) | Botswana | Both | Age-standardized | Multiple myeloma | Metabolic risks | Rate | 2021 | 3.783156 | 9.945243 | -1.46975 |
| DALYs (Disability-Adjusted Life Years) | Austria | Both | Age-standardized | Multiple myeloma | Metabolic risks | Rate | 2021 | 3.648354 | 9.606045 | -1.44673 |
| DALYs (Disability-Adjusted Life Years) | Colombia | Both | Age-standardized | Multiple myeloma | Metabolic risks | Rate | 2021 | 3.646869 | 9.689675 | -1.55187 |
| DALYs (Disability-Adjusted Life Years) | Northern Mariana Islands | Both | Age-standardized | Multiple myeloma | Metabolic risks | Rate | 2021 | 3.637333 | 9.282168 | -1.83338 |
| DALYs (Disability-Adjusted Life Years) | Saudi Arabia | Both | Age-standardized | Multiple myeloma | Metabolic risks | Rate | 2021 | 3.607852 | 9.17328 | -1.85806 |
| DALYs (Disability-Adjusted Life Years) | Seychelles | Both | Age-standardized | Multiple myeloma | Metabolic risks | Rate | 2021 | 3.586776 | 9.07082 | -1.61346 |
| DALYs (Disability-Adjusted Life Years) | Switzerland | Both | Age-standardized | Multiple myeloma | Metabolic risks | Rate | 2021 | 3.55579 | 8.870899 | -1.21195 |
| DALYs (Disability-Adjusted Life Years) | Plurinational State of Bolivia | Both | Age-standardized | Multiple myeloma | Metabolic risks | Rate | 2021 | 3.538725 | 9.777147 | -1.51151 |
| DALYs (Disability-Adjusted Life Years) | Qatar | Both | Age-standardized | Multiple myeloma | Metabolic risks | Rate | 2021 | 3.530726 | 9.796226 | -1.74989 |
| DALYs (Disability-Adjusted Life Years) | Georgia | Both | Age-standardized | Multiple myeloma | Metabolic risks | Rate | 2021 | 3.495307 | 8.975716 | -1.42495 |
| DALYs (Disability-Adjusted Life Years) | Oman | Both | Age-standardized | Multiple myeloma | Metabolic risks | Rate | 2021 | 3.487026 | 9.335146 | -1.6468 |
| DALYs (Disability-Adjusted Life Years) | Zambia | Both | Age-standardized | Multiple myeloma | Metabolic risks | Rate | 2021 | 3.398069 | 10.40644 | -1.03465 |
| DALYs (Disability-Adjusted Life Years) | Jordan | Both | Age-standardized | Multiple myeloma | Metabolic risks | Rate | 2021 | 3.34986 | 9.183769 | -1.67105 |
| DALYs (Disability-Adjusted Life Years) | Iraq | Both | Age-standardized | Multiple myeloma | Metabolic risks | Rate | 2021 | 3.345852 | 9.019176 | -1.51998 |
| DALYs (Disability-Adjusted Life Years) | Saint Vincent and the Grenadines | Both | Age-standardized | Multiple myeloma | Metabolic risks | Rate | 2021 | 3.335162 | 8.730011 | -1.23715 |
| DALYs (Disability-Adjusted Life Years) | Tunisia | Both | Age-standardized | Multiple myeloma | Metabolic risks | Rate | 2021 | 3.333837 | 8.827343 | -1.49043 |
| DALYs (Disability-Adjusted Life Years) | Malta | Both | Age-standardized | Multiple myeloma | Metabolic risks | Rate | 2021 | 3.292908 | 8.236457 | -1.28399 |
| DALYs (Disability-Adjusted Life Years) | Mauritius | Both | Age-standardized | Multiple myeloma | Metabolic risks | Rate | 2021 | 3.250455 | 8.578651 | -1.23154 |
| DALYs (Disability-Adjusted Life Years) | Romania | Both | Age-standardized | Multiple myeloma | Metabolic risks | Rate | 2021 | 3.239232 | 8.242015 | -1.40496 |
| DALYs (Disability-Adjusted Life Years) | Suriname | Both | Age-standardized | Multiple myeloma | Metabolic risks | Rate | 2021 | 3.213866 | 8.268979 | -1.1675 |
| DALYs (Disability-Adjusted Life Years) | Peru | Both | Age-standardized | Multiple myeloma | Metabolic risks | Rate | 2021 | 3.198682 | 8.674623 | -1.34756 |
| DALYs (Disability-Adjusted Life Years) | Dominican Republic | Both | Age-standardized | Multiple myeloma | Metabolic risks | Rate | 2021 | 3.182144 | 8.634436 | -1.31757 |
| DALYs (Disability-Adjusted Life Years) | Republic of San Marino | Both | Age-standardized | Multiple myeloma | Metabolic risks | Rate | 2021 | 3.076391 | 8.355497 | -1.29424 |
| DALYs (Disability-Adjusted Life Years) | Montenegro | Both | Age-standardized | Multiple myeloma | Metabolic risks | Rate | 2021 | 3.034442 | 7.573332 | -1.41435 |
| DALYs (Disability-Adjusted Life Years) | Brunei Darussalam | Both | Age-standardized | Multiple myeloma | Metabolic risks | Rate | 2021 | 2.994646 | 7.680442 | -1.02945 |
| DALYs (Disability-Adjusted Life Years) | Bulgaria | Both | Age-standardized | Multiple myeloma | Metabolic risks | Rate | 2021 | 2.965293 | 7.983309 | -1.2462 |
| DALYs (Disability-Adjusted Life Years) | Uganda | Both | Age-standardized | Multiple myeloma | Metabolic risks | Rate | 2021 | 2.913696 | 8.355476 | -0.76315 |
| DALYs (Disability-Adjusted Life Years) | Kuwait | Both | Age-standardized | Multiple myeloma | Metabolic risks | Rate | 2021 | 2.906607 | 7.343034 | -1.5286 |
| DALYs (Disability-Adjusted Life Years) | Paraguay | Both | Age-standardized | Multiple myeloma | Metabolic risks | Rate | 2021 | 2.820217 | 7.437164 | -1.18619 |
| DALYs (Disability-Adjusted Life Years) | Ukraine | Both | Age-standardized | Multiple myeloma | Metabolic risks | Rate | 2021 | 2.796354 | 7.164247 | -1.35717 |
| DALYs (Disability-Adjusted Life Years) | Andorra | Both | Age-standardized | Multiple myeloma | Metabolic risks | Rate | 2021 | 2.760175 | 7.57725 | -1.14765 |
| DALYs (Disability-Adjusted Life Years) | Gabon | Both | Age-standardized | Multiple myeloma | Metabolic risks | Rate | 2021 | 2.737565 | 7.196298 | -1.2112 |
| DALYs (Disability-Adjusted Life Years) | Namibia | Both | Age-standardized | Multiple myeloma | Metabolic risks | Rate | 2021 | 2.723056 | 7.228598 | -1.07359 |
| DALYs (Disability-Adjusted Life Years) | Honduras | Both | Age-standardized | Multiple myeloma | Metabolic risks | Rate | 2021 | 2.714484 | 8.034254 | -1.13631 |
| DALYs (Disability-Adjusted Life Years) | Turkmenistan | Both | Age-standardized | Multiple myeloma | Metabolic risks | Rate | 2021 | 2.662462 | 7.176614 | -1.06848 |
| DALYs (Disability-Adjusted Life Years) | Bosnia and Herzegovina | Both | Age-standardized | Multiple myeloma | Metabolic risks | Rate | 2021 | 2.611379 | 6.686166 | -1.11615 |
| DALYs (Disability-Adjusted Life Years) | Algeria | Both | Age-standardized | Multiple myeloma | Metabolic risks | Rate | 2021 | 2.576231 | 6.691538 | -1.08389 |
| DALYs (Disability-Adjusted Life Years) | Republic of Moldova | Both | Age-standardized | Multiple myeloma | Metabolic risks | Rate | 2021 | 2.537474 | 6.387541 | -1.14509 |
| DALYs (Disability-Adjusted Life Years) | Fiji | Both | Age-standardized | Multiple myeloma | Metabolic risks | Rate | 2021 | 2.484224 | 6.351106 | -1.11947 |
| DALYs (Disability-Adjusted Life Years) | Guyana | Both | Age-standardized | Multiple myeloma | Metabolic risks | Rate | 2021 | 2.427261 | 6.379622 | -1.00681 |
| DALYs (Disability-Adjusted Life Years) | Armenia | Both | Age-standardized | Multiple myeloma | Metabolic risks | Rate | 2021 | 2.319646 | 5.85575 | -1.00405 |
| DALYs (Disability-Adjusted Life Years) | Belize | Both | Age-standardized | Multiple myeloma | Metabolic risks | Rate | 2021 | 2.26218 | 5.611355 | -1.01781 |
| DALYs (Disability-Adjusted Life Years) | North Macedonia | Both | Age-standardized | Multiple myeloma | Metabolic risks | Rate | 2021 | 2.260199 | 5.62501 | -1.00004 |
| DALYs (Disability-Adjusted Life Years) | Pakistan | Both | Age-standardized | Multiple myeloma | Metabolic risks | Rate | 2021 | 2.224009 | 6.116882 | -0.71153 |
| DALYs (Disability-Adjusted Life Years) | Taiwan (Province of China) | Both | Age-standardized | Multiple myeloma | Metabolic risks | Rate | 2021 | 2.192047 | 5.662556 | -0.77354 |
| DALYs (Disability-Adjusted Life Years) | Islamic Republic of Iran | Both | Age-standardized | Multiple myeloma | Metabolic risks | Rate | 2021 | 2.045136 | 5.259249 | -0.91905 |
| DALYs (Disability-Adjusted Life Years) | Samoa | Both | Age-standardized | Multiple myeloma | Metabolic risks | Rate | 2021 | 2.019435 | 5.885734 | -0.95297 |
| DALYs (Disability-Adjusted Life Years) | Republic of Nauru | Both | Age-standardized | Multiple myeloma | Metabolic risks | Rate | 2021 | 1.990654 | 6.34391 | -0.9467 |
| DALYs (Disability-Adjusted Life Years) | United Republic of Tanzania | Both | Age-standardized | Multiple myeloma | Metabolic risks | Rate | 2021 | 1.989244 | 5.490615 | -0.66869 |
| DALYs (Disability-Adjusted Life Years) | Equatorial Guinea | Both | Age-standardized | Multiple myeloma | Metabolic risks | Rate | 2021 | 1.918892 | 5.436205 | -0.73945 |
| DALYs (Disability-Adjusted Life Years) | Republic of C么te d'Ivoire | Both | Age-standardized | Multiple myeloma | Metabolic risks | Rate | 2021 | 1.902593 | 5.247773 | -0.63245 |
| DALYs (Disability-Adjusted Life Years) | Morocco | Both | Age-standardized | Multiple myeloma | Metabolic risks | Rate | 2021 | 1.839598 | 5.014003 | -0.75101 |
| DALYs (Disability-Adjusted Life Years) | Comoros | Both | Age-standardized | Multiple myeloma | Metabolic risks | Rate | 2021 | 1.814943 | 5.20526 | -0.53203 |
| DALYs (Disability-Adjusted Life Years) | Kenya | Both | Age-standardized | Multiple myeloma | Metabolic risks | Rate | 2021 | 1.756253 | 4.440777 | -0.5798 |
| DALYs (Disability-Adjusted Life Years) | El Salvador | Both | Age-standardized | Multiple myeloma | Metabolic risks | Rate | 2021 | 1.668078 | 4.297497 | -0.73882 |
| DALYs (Disability-Adjusted Life Years) | Republic of Niue | Both | Age-standardized | Multiple myeloma | Metabolic risks | Rate | 2021 | 1.649583 | 4.291993 | -0.8008 |
| DALYs (Disability-Adjusted Life Years) | Kazakhstan | Both | Age-standardized | Multiple myeloma | Metabolic risks | Rate | 2021 | 1.645157 | 4.274301 | -0.74086 |
| DALYs (Disability-Adjusted Life Years) | Bhutan | Both | Age-standardized | Multiple myeloma | Metabolic risks | Rate | 2021 | 1.575152 | 4.652431 | -0.56577 |
| DALYs (Disability-Adjusted Life Years) | Syrian Arab Republic | Both | Age-standardized | Multiple myeloma | Metabolic risks | Rate | 2021 | 1.541884 | 4.13046 | -0.69413 |
| DALYs (Disability-Adjusted Life Years) | Sudan | Both | Age-standardized | Multiple myeloma | Metabolic risks | Rate | 2021 | 1.475328 | 4.242801 | -0.62316 |
| DALYs (Disability-Adjusted Life Years) | Guam | Both | Age-standardized | Multiple myeloma | Metabolic risks | Rate | 2021 | 1.464129 | 3.721931 | -0.68436 |
| DALYs (Disability-Adjusted Life Years) | Malaysia | Both | Age-standardized | Multiple myeloma | Metabolic risks | Rate | 2021 | 1.406474 | 3.773641 | -0.52202 |
| DALYs (Disability-Adjusted Life Years) | Egypt | Both | Age-standardized | Multiple myeloma | Metabolic risks | Rate | 2021 | 1.337469 | 4.129813 | -0.65393 |
| DALYs (Disability-Adjusted Life Years) | Nigeria | Both | Age-standardized | Multiple myeloma | Metabolic risks | Rate | 2021 | 1.316817 | 4.074484 | -0.46168 |
| DALYs (Disability-Adjusted Life Years) | Mozambique | Both | Age-standardized | Multiple myeloma | Metabolic risks | Rate | 2021 | 1.289747 | 3.842611 | -0.32915 |
| DALYs (Disability-Adjusted Life Years) | Guatemala | Both | Age-standardized | Multiple myeloma | Metabolic risks | Rate | 2021 | 1.273323 | 3.268434 | -0.56609 |
| DALYs (Disability-Adjusted Life Years) | Nicaragua | Both | Age-standardized | Multiple myeloma | Metabolic risks | Rate | 2021 | 1.262583 | 3.185214 | -0.56717 |
| DALYs (Disability-Adjusted Life Years) | Tokelau | Both | Age-standardized | Multiple myeloma | Metabolic risks | Rate | 2021 | 1.242524 | 3.336368 | -0.62596 |
| DALYs (Disability-Adjusted Life Years) | Federated States of Micronesia | Both | Age-standardized | Multiple myeloma | Metabolic risks | Rate | 2021 | 1.220597 | 3.644568 | -0.57728 |
| DALYs (Disability-Adjusted Life Years) | Haiti | Both | Age-standardized | Multiple myeloma | Metabolic risks | Rate | 2021 | 1.210108 | 3.988902 | -0.41302 |
| DALYs (Disability-Adjusted Life Years) | Afghanistan | Both | Age-standardized | Multiple myeloma | Metabolic risks | Rate | 2021 | 1.190195 | 4.143792 | -0.44077 |
| DALYs (Disability-Adjusted Life Years) | Rwanda | Both | Age-standardized | Multiple myeloma | Metabolic risks | Rate | 2021 | 1.134408 | 3.005955 | -0.24608 |
| DALYs (Disability-Adjusted Life Years) | Sri Lanka | Both | Age-standardized | Multiple myeloma | Metabolic risks | Rate | 2021 | 1.128855 | 3.159015 | -0.34345 |
| DALYs (Disability-Adjusted Life Years) | Congo | Both | Age-standardized | Multiple myeloma | Metabolic risks | Rate | 2021 | 1.121499 | 3.166114 | -0.4141 |
| DALYs (Disability-Adjusted Life Years) | Republic of Korea | Both | Age-standardized | Multiple myeloma | Metabolic risks | Rate | 2021 | 1.117714 | 2.941858 | -0.29012 |
| DALYs (Disability-Adjusted Life Years) | Japan | Both | Age-standardized | Multiple myeloma | Metabolic risks | Rate | 2021 | 1.063941 | 2.744763 | -0.26097 |
| DALYs (Disability-Adjusted Life Years) | Marshall Islands | Both | Age-standardized | Multiple myeloma | Metabolic risks | Rate | 2021 | 1.061844 | 3.252049 | -0.49917 |
| DALYs (Disability-Adjusted Life Years) | Albania | Both | Age-standardized | Multiple myeloma | Metabolic risks | Rate | 2021 | 1.060043 | 2.748097 | -0.45489 |
| DALYs (Disability-Adjusted Life Years) | Tuvalu | Both | Age-standardized | Multiple myeloma | Metabolic risks | Rate | 2021 | 1.03504 | 2.887303 | -0.47839 |
| DALYs (Disability-Adjusted Life Years) | Djibouti | Both | Age-standardized | Multiple myeloma | Metabolic risks | Rate | 2021 | 0.978706 | 2.790697 | -0.19648 |
| DALYs (Disability-Adjusted Life Years) | Singapore | Both | Age-standardized | Multiple myeloma | Metabolic risks | Rate | 2021 | 0.905219 | 2.335636 | -0.29393 |
| DALYs (Disability-Adjusted Life Years) | China | Both | Age-standardized | Multiple myeloma | Metabolic risks | Rate | 2021 | 0.900527 | 2.315735 | -0.29615 |
| DALYs (Disability-Adjusted Life Years) | Cook Islands | Both | Age-standardized | Multiple myeloma | Metabolic risks | Rate | 2021 | 0.883788 | 2.27358 | -0.45281 |
| DALYs (Disability-Adjusted Life Years) | Kyrgyzstan | Both | Age-standardized | Multiple myeloma | Metabolic risks | Rate | 2021 | 0.856679 | 2.219012 | -0.38752 |
| DALYs (Disability-Adjusted Life Years) | Thailand | Both | Age-standardized | Multiple myeloma | Metabolic risks | Rate | 2021 | 0.839813 | 2.651485 | -0.30785 |
| DALYs (Disability-Adjusted Life Years) | India | Both | Age-standardized | Multiple myeloma | Metabolic risks | Rate | 2021 | 0.824084 | 2.109792 | -0.27038 |
| DALYs (Disability-Adjusted Life Years) | Yemen | Both | Age-standardized | Multiple myeloma | Metabolic risks | Rate | 2021 | 0.816559 | 2.379352 | -0.27812 |
| DALYs (Disability-Adjusted Life Years) | Madagascar | Both | Age-standardized | Multiple myeloma | Metabolic risks | Rate | 2021 | 0.718104 | 2.131425 | -0.19246 |
| DALYs (Disability-Adjusted Life Years) | Maldives | Both | Age-standardized | Multiple myeloma | Metabolic risks | Rate | 2021 | 0.704206 | 1.964745 | -0.25185 |
| DALYs (Disability-Adjusted Life Years) | Azerbaijan | Both | Age-standardized | Multiple myeloma | Metabolic risks | Rate | 2021 | 0.70183 | 1.97958 | -0.29465 |
| DALYs (Disability-Adjusted Life Years) | Eritrea | Both | Age-standardized | Multiple myeloma | Metabolic risks | Rate | 2021 | 0.687346 | 2.072029 | -0.15321 |
| DALYs (Disability-Adjusted Life Years) | Angola | Both | Age-standardized | Multiple myeloma | Metabolic risks | Rate | 2021 | 0.664227 | 1.893588 | -0.17562 |
| DALYs (Disability-Adjusted Life Years) | Mongolia | Both | Age-standardized | Multiple myeloma | Metabolic risks | Rate | 2021 | 0.662108 | 1.758753 | -0.27154 |
| DALYs (Disability-Adjusted Life Years) | Malawi | Both | Age-standardized | Multiple myeloma | Metabolic risks | Rate | 2021 | 0.597597 | 1.670081 | -0.1627 |
| DALYs (Disability-Adjusted Life Years) | Nepal | Both | Age-standardized | Multiple myeloma | Metabolic risks | Rate | 2021 | 0.585683 | 1.878402 | -0.14494 |
| DALYs (Disability-Adjusted Life Years) | Vanuatu | Both | Age-standardized | Multiple myeloma | Metabolic risks | Rate | 2021 | 0.58017 | 1.64318 | -0.21911 |
| DALYs (Disability-Adjusted Life Years) | South Sudan | Both | Age-standardized | Multiple myeloma | Metabolic risks | Rate | 2021 | 0.574883 | 1.672552 | -0.0844 |
| DALYs (Disability-Adjusted Life Years) | Uzbekistan | Both | Age-standardized | Multiple myeloma | Metabolic risks | Rate | 2021 | 0.556203 | 1.459448 | -0.23579 |
| DALYs (Disability-Adjusted Life Years) | Philippines | Both | Age-standardized | Multiple myeloma | Metabolic risks | Rate | 2021 | 0.548906 | 1.482134 | -0.16591 |
| DALYs (Disability-Adjusted Life Years) | Solomon Islands | Both | Age-standardized | Multiple myeloma | Metabolic risks | Rate | 2021 | 0.536031 | 1.741965 | -0.19313 |
| DALYs (Disability-Adjusted Life Years) | Tajikistan | Both | Age-standardized | Multiple myeloma | Metabolic risks | Rate | 2021 | 0.514717 | 1.422413 | -0.20053 |
| DALYs (Disability-Adjusted Life Years) | Bangladesh | Both | Age-standardized | Multiple myeloma | Metabolic risks | Rate | 2021 | 0.500026 | 1.572447 | -0.1192 |
| DALYs (Disability-Adjusted Life Years) | Somalia | Both | Age-standardized | Multiple myeloma | Metabolic risks | Rate | 2021 | 0.482375 | 1.677309 | -0.12925 |
| DALYs (Disability-Adjusted Life Years) | Ethiopia | Both | Age-standardized | Multiple myeloma | Metabolic risks | Rate | 2021 | 0.470902 | 1.312199 | -0.10004 |
| DALYs (Disability-Adjusted Life Years) | Burundi | Both | Age-standardized | Multiple myeloma | Metabolic risks | Rate | 2021 | 0.461774 | 1.371378 | -0.08575 |
| DALYs (Disability-Adjusted Life Years) | Democratic Republic of the Congo | Both | Age-standardized | Multiple myeloma | Metabolic risks | Rate | 2021 | 0.448101 | 1.363675 | -0.14703 |
| DALYs (Disability-Adjusted Life Years) | Indonesia | Both | Age-standardized | Multiple myeloma | Metabolic risks | Rate | 2021 | 0.384271 | 1.127587 | -0.13037 |
| DALYs (Disability-Adjusted Life Years) | Central African Republic | Both | Age-standardized | Multiple myeloma | Metabolic risks | Rate | 2021 | 0.369311 | 1.0939 | -0.10545 |
| DALYs (Disability-Adjusted Life Years) | Lao People's Democratic Republic | Both | Age-standardized | Multiple myeloma | Metabolic risks | Rate | 2021 | 0.348976 | 1.239538 | -0.0993 |
| DALYs (Disability-Adjusted Life Years) | Myanmar | Both | Age-standardized | Multiple myeloma | Metabolic risks | Rate | 2021 | 0.317362 | 0.941526 | -0.08886 |
| DALYs (Disability-Adjusted Life Years) | Papua New Guinea | Both | Age-standardized | Multiple myeloma | Metabolic risks | Rate | 2021 | 0.315809 | 0.98073 | -0.10436 |
| DALYs (Disability-Adjusted Life Years) | Cambodia | Both | Age-standardized | Multiple myeloma | Metabolic risks | Rate | 2021 | 0.286198 | 0.8621 | -0.06575 |
| DALYs (Disability-Adjusted Life Years) | Cameroon | Both | Age-standardized | Multiple myeloma | Metabolic risks | Rate | 2021 | 0.280132 | 0.871322 | -0.0989 |
| DALYs (Disability-Adjusted Life Years) | Democratic People's Republic of Korea | Both | Age-standardized | Multiple myeloma | Metabolic risks | Rate | 2021 | 0.263455 | 0.861593 | -0.06831 |
| DALYs (Disability-Adjusted Life Years) | Mauritania | Both | Age-standardized | Multiple myeloma | Metabolic risks | Rate | 2021 | 0.232466 | 0.733438 | -0.0813 |
| DALYs (Disability-Adjusted Life Years) | Guinea | Both | Age-standardized | Multiple myeloma | Metabolic risks | Rate | 2021 | 0.216354 | 0.60895 | -0.05158 |
| DALYs (Disability-Adjusted Life Years) | Republic of Cabo Verde | Both | Age-standardized | Multiple myeloma | Metabolic risks | Rate | 2021 | 0.205729 | 0.632065 | -0.06895 |
| DALYs (Disability-Adjusted Life Years) | Kiribati | Both | Age-standardized | Multiple myeloma | Metabolic risks | Rate | 2021 | 0.201211 | 0.546713 | -0.09115 |
| DALYs (Disability-Adjusted Life Years) | Republic of the Gambia | Both | Age-standardized | Multiple myeloma | Metabolic risks | Rate | 2021 | 0.199617 | 0.57023 | -0.06579 |
| DALYs (Disability-Adjusted Life Years) | Sao Tome and Principe | Both | Age-standardized | Multiple myeloma | Metabolic risks | Rate | 2021 | 0.191885 | 0.578065 | -0.05972 |
| DALYs (Disability-Adjusted Life Years) | Ghana | Both | Age-standardized | Multiple myeloma | Metabolic risks | Rate | 2021 | 0.188484 | 0.601472 | -0.05217 |
| DALYs (Disability-Adjusted Life Years) | Republic of Palau | Both | Age-standardized | Multiple myeloma | Metabolic risks | Rate | 2021 | 0.158676 | 0.42626 | -0.08368 |
| DALYs (Disability-Adjusted Life Years) | Liberia | Both | Age-standardized | Multiple myeloma | Metabolic risks | Rate | 2021 | 0.150028 | 0.495825 | -0.04726 |
| DALYs (Disability-Adjusted Life Years) | Socialist Republic of Viet Nam | Both | Age-standardized | Multiple myeloma | Metabolic risks | Rate | 2021 | 0.133942 | 0.369353 | -0.02808 |
| DALYs (Disability-Adjusted Life Years) | Senegal | Both | Age-standardized | Multiple myeloma | Metabolic risks | Rate | 2021 | 0.130686 | 0.388707 | -0.03736 |
| DALYs (Disability-Adjusted Life Years) | Guinea-Bissau | Both | Age-standardized | Multiple myeloma | Metabolic risks | Rate | 2021 | 0.125249 | 0.382159 | -0.03549 |
| DALYs (Disability-Adjusted Life Years) | Benin | Both | Age-standardized | Multiple myeloma | Metabolic risks | Rate | 2021 | 0.123268 | 0.374654 | -0.03391 |
| DALYs (Disability-Adjusted Life Years) | Togo | Both | Age-standardized | Multiple myeloma | Metabolic risks | Rate | 2021 | 0.117552 | 0.367444 | -0.03402 |
| DALYs (Disability-Adjusted Life Years) | Timor-Leste | Both | Age-standardized | Multiple myeloma | Metabolic risks | Rate | 2021 | 0.110395 | 0.362925 | -0.02179 |
| DALYs (Disability-Adjusted Life Years) | Sierra Leone | Both | Age-standardized | Multiple myeloma | Metabolic risks | Rate | 2021 | 0.082123 | 0.24464 | -0.01925 |
| DALYs (Disability-Adjusted Life Years) | Chad | Both | Age-standardized | Multiple myeloma | Metabolic risks | Rate | 2021 | 0.066548 | 0.204833 | -0.01598 |
| DALYs (Disability-Adjusted Life Years) | Burkina Faso | Both | Age-standardized | Multiple myeloma | Metabolic risks | Rate | 2021 | 0.053945 | 0.164316 | -0.00804 |
| DALYs (Disability-Adjusted Life Years) | Niger | Both | Age-standardized | Multiple myeloma | Metabolic risks | Rate | 2021 | 0.046898 | 0.156349 | -0.01137 |
| DALYs (Disability-Adjusted Life Years) | Mali | Both | Age-standardized | Multiple myeloma | Metabolic risks | Rate | 2021 | 5.44E-05 | 0.000158 | -1.34E-05 |

The EAPC of multiple myeloma ASMR by age and sex from 1990 to 2021 under the influence of metabolic factors

|  | location | EAPC | LCI | UCI | EAPC_CI |
| --- | --- | --- | --- | --- | --- |
| 1 | Democratic People's Republic of Korea | 2.79 | 2.63 | 2.96 | 2.79  (2.63 to 2.96) |
| 2 | China | 6.02 | 5.37 | 6.68 | 6.02  (5.37 to 6.68) |
| 3 | Taiwan | 3.58 | 3.22 | 3.94 | 3.58  (3.22 to 3.94) |
| 4 | Indonesia | 4.36 | 4.25 | 4.47 | 4.36  (4.25 to 4.47) |
| 5 | Cambodia | 3.68 | 3.47 | 3.89 | 3.68  (3.47 to 3.89) |
| 6 | Lao People's Democratic Republic | 4.1 | 4 | 4.19 | 4.1  (4 to 4.19) |
| 7 | Malaysia | 2.83 | 2.63 | 3.04 | 2.83  (2.63 to 3.04) |
| 8 | Maldives | 2.84 | 2.71 | 2.97 | 2.84  (2.71 to 2.97) |
| 9 | Myanmar | 3.44 | 3.38 | 3.5 | 3.44  (3.38 to 3.5) |
| 10 | Philippines | 3.34 | 3.25 | 3.43 | 3.34  (3.25 to 3.43) |
| 11 | Sri Lanka | 3.29 | 3.1 | 3.47 | 3.29  (3.1 to 3.47) |
| 12 | Thailand | 3.47 | 3.26 | 3.68 | 3.47  (3.26 to 3.68) |
| 13 | Timor-Leste | 3.56 | 3.29 | 3.83 | 3.56  (3.29 to 3.83) |
| 14 | Viet Nam | 5.81 | 5.64 | 5.99 | 5.81  (5.64 to 5.99) |
| 15 | Fiji | 2.53 | 2.4 | 2.66 | 2.53  (2.4 to 2.66) |
| 16 | Kiribati | 1.46 | 1.36 | 1.56 | 1.46  (1.36 to 1.56) |
| 17 | Marshall Islands | 1.49 | 1.43 | 1.55 | 1.49  (1.43 to 1.55) |
| 18 | Micronesia (Federated States of) | 1.29 | 1.21 | 1.37 | 1.29  (1.21 to 1.37) |
| 19 | Papua New Guinea | 1.55 | 1.46 | 1.64 | 1.55  (1.46 to 1.64) |
| 20 | Samoa | 1.1 | 1.05 | 1.15 | 1.1  (1.05 to 1.15) |
| 21 | Solomon Islands | 1.91 | 1.78 | 2.04 | 1.91  (1.78 to 2.04) |
| 22 | Tonga | 1.52 | 1.34 | 1.7 | 1.52  (1.34 to 1.7) |
| 23 | Vanuatu | 1.77 | 1.68 | 1.86 | 1.77  (1.68 to 1.86) |
| 24 | Armenia | 4.77 | 4.17 | 5.38 | 4.77  (4.17 to 5.38) |
| 25 | Azerbaijan | 2.5 | 2.11 | 2.9 | 2.5  (2.11 to 2.9) |
| 26 | Georgia | 6.73 | 5.97 | 7.5 | 6.73  (5.97 to 7.5) |
| 27 | Kazakhstan | 1.93 | 1.64 | 2.23 | 1.93  (1.64 to 2.23) |
| 28 | Kyrgyzstan | 4.15 | 2.99 | 5.33 | 4.15  (2.99 to 5.33) |
| 29 | Mongolia | 2.52 | 2.38 | 2.67 | 2.52  (2.38 to 2.67) |
| 30 | Tajikistan | 0 | -0.21 | 0.21 | 0  (-0.21 to 0.21) |
| 31 | Turkmenistan | 6.47 | 5.87 | 7.07 | 6.47  (5.87 to 7.07) |
| 32 | Uzbekistan | 1.99 | 1.62 | 2.36 | 1.99  (1.62 to 2.36) |
| 33 | Albania | 2.3 | 2.13 | 2.47 | 2.3  (2.13 to 2.47) |
| 34 | Bosnia and Herzegovina | 2.5 | 2.37 | 2.63 | 2.5  (2.37 to 2.63) |
| 35 | Bulgaria | 3.06 | 2.78 | 3.33 | 3.06  (2.78 to 3.33) |
| 36 | Croatia | 1.85 | 1.64 | 2.05 | 1.85  (1.64 to 2.05) |
| 37 | Czechia | 0.21 | 0.06 | 0.36 | 0.21  (0.06 to 0.36) |
| 38 | Hungary | 0.63 | 0.38 | 0.89 | 0.63  (0.38 to 0.89) |
| 39 | North Macedonia | 1.74 | 1.57 | 1.92 | 1.74  (1.57 to 1.92) |
| 40 | Montenegro | 2.09 | 1.9 | 2.29 | 2.09  (1.9 to 2.29) |
| 41 | Romania | 2.15 | 2.03 | 2.28 | 2.15  (2.03 to 2.28) |
| 42 | Poland | 2.07 | 1.67 | 2.47 | 2.07  (1.67 to 2.47) |
| 43 | Serbia | 1.74 | 1.64 | 1.84 | 1.74  (1.64 to 1.84) |
| 44 | Slovakia | 1.09 | 0.99 | 1.18 | 1.09  (0.99 to 1.18) |
| 45 | Slovenia | 1.03 | 0.84 | 1.22 | 1.03  (0.84 to 1.22) |
| 46 | Belarus | 3.83 | 3.54 | 4.13 | 3.83  (3.54 to 4.13) |
| 47 | Estonia | 2.35 | 2.05 | 2.65 | 2.35  (2.05 to 2.65) |
| 48 | Latvia | 2.77 | 2.57 | 2.98 | 2.77  (2.57 to 2.98) |
| 49 | Lithuania | 2.6 | 2.44 | 2.76 | 2.6  (2.44 to 2.76) |
| 50 | Republic of Moldova | 2.52 | 2.16 | 2.87 | 2.52  (2.16 to 2.87) |
| 51 | Russian Federation | 2.33 | 2.11 | 2.56 | 2.33  (2.11 to 2.56) |
| 52 | Ukraine | 2.45 | 2.18 | 2.72 | 2.45  (2.18 to 2.72) |
| 53 | Brunei Darussalam | 2.2 | 2.02 | 2.37 | 2.2  (2.02 to 2.37) |
| 54 | Japan | -0.11 | -0.24 | 0.03 | -0.11  (-0.24 to 0.03) |
| 55 | Republic of Korea | 2.66 | 2.47 | 2.85 | 2.66  (2.47 to 2.85) |
| 56 | Singapore | 0.92 | 0.74 | 1.11 | 0.92  (0.74 to 1.11) |
| 57 | New Zealand | 0.99 | 0.84 | 1.13 | 0.99  (0.84 to 1.13) |
| 58 | Australia | 1.38 | 1.27 | 1.49 | 1.38  (1.27 to 1.49) |
| 59 | Andorra | 0.5 | 0.29 | 0.7 | 0.5  (0.29 to 0.7) |
| 60 | Austria | 0.72 | 0.57 | 0.87 | 0.72  (0.57 to 0.87) |
| 61 | Belgium | 0.66 | 0.52 | 0.79 | 0.66  (0.52 to 0.79) |
| 62 | Cyprus | 1.3 | 1.07 | 1.52 | 1.3  (1.07 to 1.52) |
| 63 | Denmark | 1.25 | 0.91 | 1.58 | 1.25  (0.91 to 1.58) |
| 64 | Finland | 0.52 | 0.33 | 0.7 | 0.52  (0.33 to 0.7) |
| 65 | France | 1.12 | 0.85 | 1.38 | 1.12  (0.85 to 1.38) |
| 66 | Germany | 0.67 | 0.48 | 0.87 | 0.67  (0.48 to 0.87) |
| 67 | Greece | 2.08 | 1.94 | 2.22 | 2.08  (1.94 to 2.22) |
| 68 | Iceland | 0.63 | 0.45 | 0.82 | 0.63  (0.45 to 0.82) |
| 69 | Ireland | 0.26 | 0.06 | 0.45 | 0.26  (0.06 to 0.45) |
| 70 | Israel | 0.3 | 0.1 | 0.5 | 0.3  (0.1 to 0.5) |
| 71 | Italy | 1.1 | 0.92 | 1.28 | 1.1  (0.92 to 1.28) |
| 72 | Luxembourg | 0.68 | 0.48 | 0.89 | 0.68  (0.48 to 0.89) |
| 73 | Malta | 1.11 | 0.9 | 1.32 | 1.11  (0.9 to 1.32) |
| 74 | Netherlands | 0.12 | 0.01 | 0.23 | 0.12  (0.01 to 0.23) |
| 75 | Norway | 0.16 | 0.08 | 0.25 | 0.16  (0.08 to 0.25) |
| 76 | Spain | 0.52 | 0.27 | 0.78 | 0.52  (0.27 to 0.78) |
| 77 | Portugal | 1.3 | 1.08 | 1.52 | 1.3  (1.08 to 1.52) |
| 78 | Sweden | 0 | -0.09 | 0.1 | 0  (-0.09 to 0.1) |
| 79 | Switzerland | 0.1 | -0.21 | 0.42 | 0.1  (-0.21 to 0.42) |
| 80 | United Kingdom | 0.67 | 0.58 | 0.77 | 0.67  (0.58 to 0.77) |
| 81 | Argentina | 0.53 | 0.36 | 0.71 | 0.53  (0.36 to 0.71) |
| 82 | Chile | 0.81 | 0.55 | 1.08 | 0.81  (0.55 to 1.08) |
| 83 | Uruguay | 0.98 | 0.87 | 1.1 | 0.98  (0.87 to 1.1) |
| 84 | United States of America | 0.24 | 0.06 | 0.43 | 0.24  (0.06 to 0.43) |
| 85 | Canada | 0.01 | -0.12 | 0.14 | 0.01  (-0.12 to 0.14) |
| 86 | Antigua and Barbuda | 1.9 | 1.67 | 2.13 | 1.9  (1.67 to 2.13) |
| 87 | Bahamas | 1.35 | 1.29 | 1.41 | 1.35  (1.29 to 1.41) |
| 88 | Barbados | 1.53 | 1.41 | 1.65 | 1.53  (1.41 to 1.65) |
| 89 | Belize | 1.52 | 1.12 | 1.91 | 1.52  (1.12 to 1.91) |
| 90 | Dominica | 1.31 | 1.21 | 1.4 | 1.31  (1.21 to 1.4) |
| 91 | Cuba | 1.93 | 1.83 | 2.02 | 1.93  (1.83 to 2.02) |
| 92 | Dominican Republic | 3.74 | 3.59 | 3.89 | 3.74  (3.59 to 3.89) |
| 93 | Grenada | 3.18 | 2.9 | 3.47 | 3.18  (2.9 to 3.47) |
| 94 | Guyana | 4.52 | 3.55 | 5.49 | 4.52  (3.55 to 5.49) |
| 95 | Haiti | 2.62 | 2.53 | 2.71 | 2.62  (2.53 to 2.71) |
| 96 | Jamaica | 4.2 | 3.75 | 4.65 | 4.2  (3.75 to 4.65) |
| 97 | Saint Lucia | 1.25 | 1.07 | 1.43 | 1.25  (1.07 to 1.43) |
| 98 | Saint Vincent and the Grenadines | 2.17 | 1.99 | 2.34 | 2.17  (1.99 to 2.34) |
| 99 | Suriname | 2.35 | 2.19 | 2.52 | 2.35  (2.19 to 2.52) |
| 100 | Trinidad and Tobago | 1.27 | 1.14 | 1.4 | 1.27  (1.14 to 1.4) |
| 101 | Ecuador | 5.1 | 4.79 | 5.4 | 5.1  (4.79 to 5.4) |
| 102 | Bolivia (Plurinational State of) | 2.28 | 2.23 | 2.33 | 2.28  (2.23 to 2.33) |
| 103 | Peru | 1.56 | 1.32 | 1.79 | 1.56  (1.32 to 1.79) |
| 104 | Costa Rica | 2.76 | 2.56 | 2.95 | 2.76  (2.56 to 2.95) |
| 105 | Guatemala | 1.98 | 1.85 | 2.11 | 1.98  (1.85 to 2.11) |
| 106 | Colombia | 2.03 | 1.84 | 2.22 | 2.03  (1.84 to 2.22) |
| 107 | El Salvador | 2.46 | 2.37 | 2.55 | 2.46  (2.37 to 2.55) |
| 108 | Honduras | 3.09 | 2.97 | 3.21 | 3.09  (2.97 to 3.21) |
| 109 | Nicaragua | 2.25 | 2.1 | 2.4 | 2.25  (2.1 to 2.4) |
| 110 | Mexico | 1.84 | 1.72 | 1.95 | 1.84  (1.72 to 1.95) |
| 111 | Venezuela (Bolivarian Republic of) | 1.45 | 1.28 | 1.62 | 1.45  (1.28 to 1.62) |
| 112 | Panama | 1.57 | 1.41 | 1.74 | 1.57  (1.41 to 1.74) |
| 113 | Brazil | 2.35 | 2.18 | 2.53 | 2.35  (2.18 to 2.53) |
| 114 | Algeria | 2.95 | 2.81 | 3.09 | 2.95  (2.81 to 3.09) |
| 115 | Paraguay | 2.66 | 2.55 | 2.77 | 2.66  (2.55 to 2.77) |
| 116 | Egypt | 2.7 | 2.53 | 2.87 | 2.7  (2.53 to 2.87) |
| 117 | Iraq | 2.17 | 1.97 | 2.38 | 2.17  (1.97 to 2.38) |
| 118 | Bahrain | 1.75 | 1.59 | 1.92 | 1.75  (1.59 to 1.92) |
| 119 | Kuwait | 1.07 | 0.23 | 1.92 | 1.07  (0.23 to 1.92) |
| 120 | Iran (Islamic Republic of) | 4.01 | 3.89 | 4.12 | 4.01  (3.89 to 4.12) |
| 121 | Jordan | 0.62 | 0.36 | 0.87 | 0.62  (0.36 to 0.87) |
| 122 | Libya | 2.67 | 2.43 | 2.91 | 2.67  (2.43 to 2.91) |
| 123 | Lebanon | 1.9 | 1.72 | 2.08 | 1.9  (1.72 to 2.08) |
| 124 | Palestine | 1.89 | 1.82 | 1.96 | 1.89  (1.82 to 1.96) |
| 125 | Qatar | 1.18 | 0.81 | 1.56 | 1.18  (0.81 to 1.56) |
| 126 | Morocco | 3.55 | 3.41 | 3.68 | 3.55  (3.41 to 3.68) |
| 127 | Oman | 3.99 | 3.62 | 4.36 | 3.99  (3.62 to 4.36) |
| 128 | Syrian Arab Republic | 2.92 | 2.77 | 3.08 | 2.92  (2.77 to 3.08) |
| 129 | T眉rkiye | 1.81 | 1.61 | 2.01 | 1.81  (1.61 to 2.01) |
| 130 | Saudi Arabia | 3.31 | 3.2 | 3.41 | 3.31  (3.2 to 3.41) |
| 131 | Yemen | 3.55 | 3.42 | 3.69 | 3.55  (3.42 to 3.69) |
| 132 | Tunisia | 3.04 | 2.96 | 3.12 | 3.04  (2.96 to 3.12) |
| 133 | United Arab Emirates | 3.51 | 3.07 | 3.96 | 3.51  (3.07 to 3.96) |
| 134 | Afghanistan | 2.13 | 1.78 | 2.47 | 2.13  (1.78 to 2.47) |
| 135 | Bhutan | 3.1 | 3.04 | 3.15 | 3.1  (3.04 to 3.15) |
| 136 | Bangladesh | 4.19 | 4.04 | 4.35 | 4.19  (4.04 to 4.35) |
| 137 | Nepal | 4.6 | 4.29 | 4.91 | 4.6  (4.29 to 4.91) |
| 138 | India | 4.7 | 4.6 | 4.8 | 4.7  (4.6 to 4.8) |
| 139 | Pakistan | 4.16 | 3.97 | 4.36 | 4.16  (3.97 to 4.36) |
| 140 | Angola | 4.11 | 3.94 | 4.27 | 4.11  (3.94 to 4.27) |
| 141 | Central African Republic | 2.5 | 2.45 | 2.55 | 2.5  (2.45 to 2.55) |
| 142 | Congo | 2.74 | 2.63 | 2.85 | 2.74  (2.63 to 2.85) |
| 143 | Equatorial Guinea | 6.54 | 6.29 | 6.79 | 6.54  (6.29 to 6.79) |
| 144 | Democratic Republic of the Congo | 2.77 | 2.33 | 3.21 | 2.77  (2.33 to 3.21) |
| 145 | Gabon | 2.59 | 2.51 | 2.67 | 2.59  (2.51 to 2.67) |
| 146 | Comoros | 2.84 | 2.75 | 2.93 | 2.84  (2.75 to 2.93) |
| 147 | Burundi | 0.2 | -0.06 | 0.46 | 0.2  (-0.06 to 0.46) |
| 148 | Eritrea | 2.91 | 2.74 | 3.09 | 2.91  (2.74 to 3.09) |
| 149 | Kenya | 4.32 | 4.21 | 4.43 | 4.32  (4.21 to 4.43) |
| 150 | Djibouti | 2.42 | 2.33 | 2.51 | 2.42  (2.33 to 2.51) |
| 151 | Ethiopia | 2.56 | 2.22 | 2.9 | 2.56  (2.22 to 2.9) |
| 152 | Malawi | 3.2 | 3.13 | 3.27 | 3.2  (3.13 to 3.27) |
| 153 | Madagascar | 1.81 | 1.55 | 2.06 | 1.81  (1.55 to 2.06) |
| 154 | Mozambique | 3.69 | 3.55 | 3.83 | 3.69  (3.55 to 3.83) |
| 155 | Mauritius | 4.99 | 4.08 | 5.92 | 4.99  (4.08 to 5.92) |
| 156 | Seychelles | 2.54 | 2.33 | 2.75 | 2.54  (2.33 to 2.75) |
| 157 | Rwanda | 1.55 | 1.26 | 1.84 | 1.55  (1.26 to 1.84) |
| 158 | United Republic of Tanzania | 2.79 | 2.72 | 2.87 | 2.79  (2.72 to 2.87) |
| 159 | Zambia | 5.07 | 4.69 | 5.46 | 5.07  (4.69 to 5.46) |
| 160 | Somalia | 1.14 | 1.06 | 1.22 | 1.14  (1.06 to 1.22) |
| 161 | Uganda | 3.52 | 3.4 | 3.65 | 3.52  (3.4 to 3.65) |
| 162 | Lesotho | 4.36 | 4.05 | 4.66 | 4.36  (4.05 to 4.66) |
| 163 | South Africa | 2.46 | 2.34 | 2.58 | 2.46  (2.34 to 2.58) |
| 164 | Botswana | 4.14 | 3.95 | 4.34 | 4.14  (3.95 to 4.34) |
| 165 | Namibia | 3.68 | 3.52 | 3.84 | 3.68  (3.52 to 3.84) |
| 166 | Zimbabwe | 3.95 | 3.48 | 4.41 | 3.95  (3.48 to 4.41) |
| 167 | Eswatini | 3.57 | 3.19 | 3.95 | 3.57  (3.19 to 3.95) |
| 168 | Benin | 5 | 4.85 | 5.15 | 5  (4.85 to 5.15) |
| 169 | Cameroon | 5.15 | 4.97 | 5.32 | 5.15  (4.97 to 5.32) |
| 170 | Chad | 5.69 | 5.46 | 5.92 | 5.69  (5.46 to 5.92) |
| 171 | Burkina Faso | 5.11 | 4.98 | 5.25 | 5.11  (4.98 to 5.25) |
| 172 | Cabo Verde | 4.68 | 4.61 | 4.75 | 4.68  (4.61 to 4.75) |
| 173 | Gambia | 2.95 | 2.77 | 3.12 | 2.95  (2.77 to 3.12) |
| 174 | C么te d'Ivoire | 2.52 | 2.47 | 2.58 | 2.52  (2.47 to 2.58) |
| 175 | Guinea | 4.06 | 4 | 4.12 | 4.06  (4 to 4.12) |
| 176 | Ghana | 7.52 | 7.31 | 7.73 | 7.52  (7.31 to 7.73) |
| 177 | Guinea-Bissau | 5.12 | 4.96 | 5.28 | 5.12  (4.96 to 5.28) |
| 178 | Liberia | 4.69 | 4.33 | 5.05 | 4.69  (4.33 to 5.05) |
| 179 | Mauritania | 4.85 | 4.72 | 4.99 | 4.85  (4.72 to 4.99) |
| 180 | Mali | 1.97 | 1.85 | 2.08 | 1.97  (1.85 to 2.08) |
| 181 | Nigeria | 4.35 | 4.18 | 4.52 | 4.35  (4.18 to 4.52) |
| 182 | Niger | 4.46 | 4.29 | 4.63 | 4.46  (4.29 to 4.63) |
| 183 | Senegal | 5.12 | 4.93 | 5.31 | 5.12  (4.93 to 5.31) |
| 184 | Sao Tome and Principe | 6.28 | 6.08 | 6.47 | 6.28  (6.08 to 6.47) |
| 185 | Togo | 5.74 | 5.58 | 5.89 | 5.74  (5.58 to 5.89) |
| 186 | Sierra Leone | 5.31 | 5.09 | 5.53 | 5.31  (5.09 to 5.53) |
| 187 | Bermuda | 0.41 | 0.21 | 0.62 | 0.41  (0.21 to 0.62) |
| 188 | American Samoa | 1.3 | 1.05 | 1.54 | 1.3  (1.05 to 1.54) |
| 189 | Greenland | -0.21 | -0.38 | -0.04 | -0.21  (-0.38 to -0.04) |
| 190 | Cook Islands | 1.66 | 1.45 | 1.86 | 1.66  (1.45 to 1.86) |
| 191 | Monaco | 1.3 | 1.28 | 1.32 | 1.3  (1.28 to 1.32) |
| 192 | Guam | 0.51 | 0.23 | 0.79 | 0.51  (0.23 to 0.79) |
| 193 | Niue | 1.83 | 1.76 | 1.91 | 1.83  (1.76 to 1.91) |
| 194 | Nauru | 0.71 | 0.47 | 0.95 | 0.71  (0.47 to 0.95) |
| 195 | Northern Mariana Islands | -0.46 | -0.69 | -0.23 | -0.46  (-0.69 to -0.23) |
| 196 | Palau | 1.02 | 0.96 | 1.08 | 1.02  (0.96 to 1.08) |
| 197 | Saint Kitts and Nevis | 1.44 | 1.21 | 1.67 | 1.44  (1.21 to 1.67) |
| 198 | Tokelau | 1.86 | 1.81 | 1.91 | 1.86  (1.81 to 1.91) |
| 199 | Puerto Rico | 0.54 | 0.35 | 0.74 | 0.54  (0.35 to 0.74) |
| 200 | San Marino | 0.16 | -0.26 | 0.57 | 0.16  (-0.26 to 0.57) |
| 201 | United States Virgin Islands | 0.28 | 0.04 | 0.51 | 0.28  (0.04 to 0.51) |
| 202 | Tuvalu | 2.13 | 2.03 | 2.24 | 2.13  (2.03 to 2.24) |
| 203 | South Sudan | 1.3 | 1.16 | 1.43 | 1.3  (1.16 to 1.43) |
| 204 | Sudan | 2.87 | 2.72 | 3.03 | 2.87  (2.72 to 3.03) |

The EAPC of ASDR of multiple myeloma across all ages and genders from 1990 to 2021 under the influence of metabolic factors

|  | location | EAPC | LCI | UCI | EAPC_CI |
| --- | --- | --- | --- | --- | --- |
| 1 | China | 6.09 | 5.47 | 6.71 | 6.09  (5.47 to 6.71) |
| 2 | Democratic People's Republic of Korea | 2.52 | 2.35 | 2.68 | 2.52  (2.35 to 2.68) |
| 3 | Taiwan | 3.51 | 3.19 | 3.84 | 3.51  (3.19 to 3.84) |
| 4 | Cambodia | 3.62 | 3.42 | 3.83 | 3.62  (3.42 to 3.83) |
| 5 | Indonesia | 4.27 | 4.13 | 4.41 | 4.27  (4.13 to 4.41) |
| 6 | Maldives | 2.79 | 2.66 | 2.93 | 2.79  (2.66 to 2.93) |
| 7 | Lao People's Democratic Republic | 4.12 | 4.01 | 4.23 | 4.12  (4.01 to 4.23) |
| 8 | Myanmar | 3.32 | 3.27 | 3.37 | 3.32  (3.27 to 3.37) |
| 9 | Malaysia | 2.7 | 2.52 | 2.87 | 2.7  (2.52 to 2.87) |
| 10 | Philippines | 3.23 | 3.14 | 3.33 | 3.23  (3.14 to 3.33) |
| 11 | Sri Lanka | 3.13 | 2.94 | 3.32 | 3.13  (2.94 to 3.32) |
| 12 | Viet Nam | 5.95 | 5.75 | 6.16 | 5.95  (5.75 to 6.16) |
| 13 | Thailand | 3.46 | 3.24 | 3.68 | 3.46  (3.24 to 3.68) |
| 14 | Timor-Leste | 3.73 | 3.44 | 4.02 | 3.73  (3.44 to 4.02) |
| 15 | Fiji | 2.5 | 2.33 | 2.66 | 2.5  (2.33 to 2.66) |
| 16 | Micronesia (Federated States of) | 1.23 | 1.16 | 1.31 | 1.23  (1.16 to 1.31) |
| 17 | Papua New Guinea | 1.62 | 1.53 | 1.71 | 1.62  (1.53 to 1.71) |
| 18 | Kiribati | 1.33 | 1.22 | 1.43 | 1.33  (1.22 to 1.43) |
| 19 | Samoa | 1.07 | 1.03 | 1.11 | 1.07  (1.03 to 1.11) |
| 20 | Marshall Islands | 1.42 | 1.36 | 1.49 | 1.42  (1.36 to 1.49) |
| 21 | Vanuatu | 1.82 | 1.71 | 1.93 | 1.82  (1.71 to 1.93) |
| 22 | Solomon Islands | 1.98 | 1.85 | 2.12 | 1.98  (1.85 to 2.12) |
| 23 | Armenia | 4.74 | 4.13 | 5.35 | 4.74  (4.13 to 5.35) |
| 24 | Tonga | 1.4 | 1.23 | 1.57 | 1.4  (1.23 to 1.57) |
| 25 | Kazakhstan | 1.84 | 1.58 | 2.1 | 1.84  (1.58 to 2.1) |
| 26 | Azerbaijan | 2.3 | 1.91 | 2.7 | 2.3  (1.91 to 2.7) |
| 27 | Kyrgyzstan | 4.29 | 3.28 | 5.32 | 4.29  (3.28 to 5.32) |
| 28 | Mongolia | 2.62 | 2.47 | 2.77 | 2.62  (2.47 to 2.77) |
| 29 | Georgia | 6.59 | 5.83 | 7.34 | 6.59  (5.83 to 7.34) |
| 30 | Uzbekistan | 2.11 | 1.74 | 2.47 | 2.11  (1.74 to 2.47) |
| 31 | Tajikistan | -0.02 | -0.25 | 0.22 | -0.02  (-0.25 to 0.22) |
| 32 | Croatia | 1.69 | 1.47 | 1.92 | 1.69  (1.47 to 1.92) |
| 33 | Turkmenistan | 6.53 | 5.94 | 7.14 | 6.53  (5.94 to 7.14) |
| 34 | Albania | 2.26 | 2.08 | 2.44 | 2.26  (2.08 to 2.44) |
| 35 | Bosnia and Herzegovina | 2.42 | 2.26 | 2.58 | 2.42  (2.26 to 2.58) |
| 36 | Czechia | 0.04 | -0.1 | 0.18 | 0.04  (-0.1 to 0.18) |
| 37 | Bulgaria | 3 | 2.74 | 3.27 | 3  (2.74 to 3.27) |
| 38 | Hungary | 0.46 | 0.21 | 0.72 | 0.46  (0.21 to 0.72) |
| 39 | North Macedonia | 1.55 | 1.39 | 1.7 | 1.55  (1.39 to 1.7) |
| 40 | Romania | 1.91 | 1.77 | 2.04 | 1.91  (1.77 to 2.04) |
| 41 | Serbia | 1.73 | 1.63 | 1.83 | 1.73  (1.63 to 1.83) |
| 42 | Montenegro | 1.9 | 1.76 | 2.05 | 1.9  (1.76 to 2.05) |
| 43 | Belarus | 3.84 | 3.52 | 4.17 | 3.84  (3.52 to 4.17) |
| 44 | Poland | 1.63 | 1.24 | 2.01 | 1.63  (1.24 to 2.01) |
| 45 | Slovakia | 0.95 | 0.86 | 1.04 | 0.95  (0.86 to 1.04) |
| 46 | Estonia | 2.16 | 1.81 | 2.52 | 2.16  (1.81 to 2.52) |
| 47 | Slovenia | 0.71 | 0.51 | 0.9 | 0.71  (0.51 to 0.9) |
| 48 | Latvia | 2.79 | 2.56 | 3.02 | 2.79  (2.56 to 3.02) |
| 49 | Lithuania | 2.63 | 2.43 | 2.82 | 2.63  (2.43 to 2.82) |
| 50 | Republic of Moldova | 2.5 | 2.15 | 2.86 | 2.5  (2.15 to 2.86) |
| 51 | Russian Federation | 1.95 | 1.75 | 2.15 | 1.95  (1.75 to 2.15) |
| 52 | Ukraine | 2.33 | 2.08 | 2.58 | 2.33  (2.08 to 2.58) |
| 53 | Brunei Darussalam | 2.15 | 1.99 | 2.3 | 2.15  (1.99 to 2.3) |
| 54 | Japan | -0.43 | -0.56 | -0.29 | -0.43  (-0.56 to -0.29) |
| 55 | Republic of Korea | 2.37 | 2.13 | 2.6 | 2.37  (2.13 to 2.6) |
| 56 | Singapore | 0.5 | 0.28 | 0.72 | 0.5  (0.28 to 0.72) |
| 57 | Australia | 1.18 | 1.08 | 1.28 | 1.18  (1.08 to 1.28) |
| 58 | New Zealand | 0.77 | 0.64 | 0.91 | 0.77  (0.64 to 0.91) |
| 59 | Andorra | 0.45 | 0.25 | 0.65 | 0.45  (0.25 to 0.65) |
| 60 | Austria | 0.46 | 0.29 | 0.64 | 0.46  (0.29 to 0.64) |
| 61 | Belgium | 0.53 | 0.33 | 0.74 | 0.53  (0.33 to 0.74) |
| 62 | Cyprus | 1.37 | 1.12 | 1.62 | 1.37  (1.12 to 1.62) |
| 63 | Denmark | 0.79 | 0.46 | 1.13 | 0.79  (0.46 to 1.13) |
| 64 | Finland | 0.28 | 0.09 | 0.47 | 0.28  (0.09 to 0.47) |
| 65 | France | 0.91 | 0.63 | 1.18 | 0.91  (0.63 to 1.18) |
| 66 | Germany | 0.43 | 0.23 | 0.64 | 0.43  (0.23 to 0.64) |
| 67 | Greece | 1.97 | 1.86 | 2.08 | 1.97  (1.86 to 2.08) |
| 68 | Iceland | 0.37 | 0.2 | 0.55 | 0.37  (0.2 to 0.55) |
| 69 | Ireland | 0.03 | -0.18 | 0.23 | 0.03  (-0.18 to 0.23) |
| 70 | Israel | 0.18 | -0.03 | 0.4 | 0.18  (-0.03 to 0.4) |
| 71 | Italy | 0.74 | 0.56 | 0.92 | 0.74  (0.56 to 0.92) |
| 72 | Luxembourg | 0.48 | 0.26 | 0.69 | 0.48  (0.26 to 0.69) |
| 73 | Malta | 1.11 | 0.9 | 1.32 | 1.11  (0.9 to 1.32) |
| 74 | Netherlands | 0.02 | -0.09 | 0.12 | 0.02  (-0.09 to 0.12) |
| 75 | Norway | -0.07 | -0.19 | 0.05 | -0.07  (-0.19 to 0.05) |
| 76 | Portugal | 0.95 | 0.72 | 1.18 | 0.95  (0.72 to 1.18) |
| 77 | Spain | 0.17 | -0.08 | 0.42 | 0.17  (-0.08 to 0.42) |
| 78 | Sweden | -0.2 | -0.35 | -0.04 | -0.2  (-0.35 to -0.04) |
| 79 | Switzerland | -0.27 | -0.57 | 0.05 | -0.27  (-0.57 to 0.05) |
| 80 | United Kingdom | 0.41 | 0.31 | 0.51 | 0.41  (0.31 to 0.51) |
| 81 | Argentina | 0.49 | 0.32 | 0.66 | 0.49  (0.32 to 0.66) |
| 82 | Chile | 0.65 | 0.4 | 0.91 | 0.65  (0.4 to 0.91) |
| 83 | Uruguay | 0.88 | 0.77 | 0.99 | 0.88  (0.77 to 0.99) |
| 84 | Canada | -0.33 | -0.46 | -0.19 | -0.33  (-0.46 to -0.19) |
| 85 | United States of America | -0.15 | -0.34 | 0.04 | -0.15  (-0.34 to 0.04) |
| 86 | Antigua and Barbuda | 1.79 | 1.59 | 2 | 1.79  (1.59 to 2) |
| 87 | Bahamas | 1.27 | 1.21 | 1.32 | 1.27  (1.21 to 1.32) |
| 88 | Barbados | 1.45 | 1.34 | 1.57 | 1.45  (1.34 to 1.57) |
| 89 | Belize | 1.55 | 1.18 | 1.93 | 1.55  (1.18 to 1.93) |
| 90 | Cuba | 1.89 | 1.77 | 2.01 | 1.89  (1.77 to 2.01) |
| 91 | Dominica | 1.41 | 1.31 | 1.51 | 1.41  (1.31 to 1.51) |
| 92 | Dominican Republic | 3.73 | 3.6 | 3.86 | 3.73  (3.6 to 3.86) |
| 93 | Grenada | 3.03 | 2.8 | 3.27 | 3.03  (2.8 to 3.27) |
| 94 | Guyana | 4.5 | 3.55 | 5.45 | 4.5  (3.55 to 5.45) |
| 95 | Haiti | 2.59 | 2.5 | 2.68 | 2.59  (2.5 to 2.68) |
| 96 | Jamaica | 4.07 | 3.61 | 4.52 | 4.07  (3.61 to 4.52) |
| 97 | Saint Lucia | 1.31 | 1.13 | 1.49 | 1.31  (1.13 to 1.49) |
| 98 | Saint Vincent and the Grenadines | 2.24 | 2.09 | 2.39 | 2.24  (2.09 to 2.39) |
| 99 | Suriname | 2.34 | 2.19 | 2.5 | 2.34  (2.19 to 2.5) |
| 100 | Trinidad and Tobago | 1.32 | 1.19 | 1.45 | 1.32  (1.19 to 1.45) |
| 101 | Bolivia (Plurinational State of) | 2.12 | 2.07 | 2.17 | 2.12  (2.07 to 2.17) |
| 102 | Ecuador | 4.92 | 4.61 | 5.22 | 4.92  (4.61 to 5.22) |
| 103 | Peru | 1.52 | 1.28 | 1.76 | 1.52  (1.28 to 1.76) |
| 104 | Colombia | 1.92 | 1.73 | 2.1 | 1.92  (1.73 to 2.1) |
| 105 | Costa Rica | 2.97 | 2.76 | 3.17 | 2.97  (2.76 to 3.17) |
| 106 | El Salvador | 2.47 | 2.38 | 2.57 | 2.47  (2.38 to 2.57) |
| 107 | Guatemala | 2.08 | 1.94 | 2.22 | 2.08  (1.94 to 2.22) |
| 108 | Honduras | 2.94 | 2.82 | 3.06 | 2.94  (2.82 to 3.06) |
| 109 | Mexico | 1.83 | 1.71 | 1.95 | 1.83  (1.71 to 1.95) |
| 110 | Nicaragua | 2.25 | 2.11 | 2.39 | 2.25  (2.11 to 2.39) |
| 111 | Panama | 1.71 | 1.55 | 1.87 | 1.71  (1.55 to 1.87) |
| 112 | Venezuela (Bolivarian Republic of) | 1.41 | 1.24 | 1.58 | 1.41  (1.24 to 1.58) |
| 113 | Brazil | 2.03 | 1.85 | 2.2 | 2.03  (1.85 to 2.2) |
| 114 | Paraguay | 2.56 | 2.46 | 2.65 | 2.56  (2.46 to 2.65) |
| 115 | Algeria | 2.78 | 2.67 | 2.88 | 2.78  (2.67 to 2.88) |
| 116 | Bahrain | 1.57 | 1.44 | 1.7 | 1.57  (1.44 to 1.7) |
| 117 | Egypt | 2.7 | 2.53 | 2.87 | 2.7  (2.53 to 2.87) |
| 118 | Iran (Islamic Republic of) | 3.83 | 3.72 | 3.94 | 3.83  (3.72 to 3.94) |
| 119 | Iraq | 2 | 1.82 | 2.18 | 2  (1.82 to 2.18) |
| 120 | Jordan | 0.51 | 0.25 | 0.77 | 0.51  (0.25 to 0.77) |
| 121 | Kuwait | 0.98 | 0.22 | 1.75 | 0.98  (0.22 to 1.75) |
| 122 | Lebanon | 1.7 | 1.53 | 1.87 | 1.7  (1.53 to 1.87) |
| 123 | Libya | 2.59 | 2.35 | 2.82 | 2.59  (2.35 to 2.82) |
| 124 | Morocco | 3.48 | 3.36 | 3.6 | 3.48  (3.36 to 3.6) |
| 125 | Palestine | 1.82 | 1.77 | 1.87 | 1.82  (1.77 to 1.87) |
| 126 | Oman | 3.75 | 3.37 | 4.14 | 3.75  (3.37 to 4.14) |
| 127 | Qatar | 1.19 | 0.87 | 1.51 | 1.19  (0.87 to 1.51) |
| 128 | Saudi Arabia | 3.4 | 3.29 | 3.52 | 3.4  (3.29 to 3.52) |
| 129 | Syrian Arab Republic | 2.79 | 2.65 | 2.93 | 2.79  (2.65 to 2.93) |
| 130 | Tunisia | 2.95 | 2.89 | 3.02 | 2.95  (2.89 to 3.02) |
| 131 | T眉rkiye | 1.68 | 1.48 | 1.87 | 1.68  (1.48 to 1.87) |
| 132 | United Arab Emirates | 2.58 | 2.22 | 2.95 | 2.58  (2.22 to 2.95) |
| 133 | Yemen | 3.38 | 3.24 | 3.51 | 3.38  (3.24 to 3.51) |
| 134 | Afghanistan | 2.05 | 1.71 | 2.38 | 2.05  (1.71 to 2.38) |
| 135 | Bangladesh | 4.39 | 4.2 | 4.58 | 4.39  (4.2 to 4.58) |
| 136 | Bhutan | 2.93 | 2.87 | 2.98 | 2.93  (2.87 to 2.98) |
| 137 | India | 4.56 | 4.47 | 4.65 | 4.56  (4.47 to 4.65) |
| 138 | Nepal | 4.55 | 4.23 | 4.86 | 4.55  (4.23 to 4.86) |
| 139 | Pakistan | 4.16 | 3.96 | 4.35 | 4.16  (3.96 to 4.35) |
| 140 | Angola | 4.03 | 3.86 | 4.2 | 4.03  (3.86 to 4.2) |
| 141 | Central African Republic | 2.48 | 2.42 | 2.53 | 2.48  (2.42 to 2.53) |
| 142 | Congo | 2.61 | 2.5 | 2.72 | 2.61  (2.5 to 2.72) |
| 143 | Democratic Republic of the Congo | 2.74 | 2.3 | 3.18 | 2.74  (2.3 to 3.18) |
| 144 | Equatorial Guinea | 6.19 | 5.95 | 6.43 | 6.19  (5.95 to 6.43) |
| 145 | Gabon | 2.45 | 2.36 | 2.53 | 2.45  (2.36 to 2.53) |
| 146 | Burundi | 0.07 | -0.2 | 0.35 | 0.07  (-0.2 to 0.35) |
| 147 | Comoros | 2.72 | 2.61 | 2.84 | 2.72  (2.61 to 2.84) |
| 148 | Djibouti | 2.38 | 2.29 | 2.46 | 2.38  (2.29 to 2.46) |
| 149 | Eritrea | 2.82 | 2.64 | 3 | 2.82  (2.64 to 3) |
| 150 | Ethiopia | 2.35 | 2.02 | 2.68 | 2.35  (2.02 to 2.68) |
| 151 | Kenya | 4.3 | 4.2 | 4.41 | 4.3  (4.2 to 4.41) |
| 152 | Madagascar | 1.77 | 1.51 | 2.03 | 1.77  (1.51 to 2.03) |
| 153 | Malawi | 3.25 | 3.17 | 3.32 | 3.25  (3.17 to 3.32) |
| 154 | Mauritius | 4.99 | 4.07 | 5.91 | 4.99  (4.07 to 5.91) |
| 155 | Mozambique | 3.78 | 3.64 | 3.92 | 3.78  (3.64 to 3.92) |
| 156 | Rwanda | 1.34 | 1.02 | 1.66 | 1.34  (1.02 to 1.66) |
| 157 | Seychelles | 2.37 | 2.17 | 2.57 | 2.37  (2.17 to 2.57) |
| 158 | Somalia | 1.07 | 0.99 | 1.16 | 1.07  (0.99 to 1.16) |
| 159 | United Republic of Tanzania | 2.68 | 2.61 | 2.75 | 2.68  (2.61 to 2.75) |
| 160 | Uganda | 3.46 | 3.31 | 3.61 | 3.46  (3.31 to 3.61) |
| 161 | Zambia | 5.16 | 4.77 | 5.55 | 5.16  (4.77 to 5.55) |
| 162 | Botswana | 3.97 | 3.78 | 4.15 | 3.97  (3.78 to 4.15) |
| 163 | Lesotho | 4.4 | 4.09 | 4.72 | 4.4  (4.09 to 4.72) |
| 164 | Namibia | 3.53 | 3.35 | 3.72 | 3.53  (3.35 to 3.72) |
| 165 | South Africa | 2.39 | 2.27 | 2.52 | 2.39  (2.27 to 2.52) |
| 166 | Eswatini | 3.64 | 3.21 | 4.06 | 3.64  (3.21 to 4.06) |
| 167 | Zimbabwe | 3.99 | 3.47 | 4.51 | 3.99  (3.47 to 4.51) |
| 168 | Benin | 4.78 | 4.64 | 4.92 | 4.78  (4.64 to 4.92) |
| 169 | Burkina Faso | 5.03 | 4.91 | 5.16 | 5.03  (4.91 to 5.16) |
| 170 | Cameroon | 4.98 | 4.81 | 5.15 | 4.98  (4.81 to 5.15) |
| 171 | Cabo Verde | 4.51 | 4.44 | 4.57 | 4.51  (4.44 to 4.57) |
| 172 | Chad | 5.59 | 5.36 | 5.81 | 5.59  (5.36 to 5.81) |
| 173 | C么te d'Ivoire | 2.45 | 2.37 | 2.52 | 2.45  (2.37 to 2.52) |
| 174 | Gambia | 2.87 | 2.67 | 3.07 | 2.87  (2.67 to 3.07) |
| 175 | Ghana | 7.22 | 7.01 | 7.42 | 7.22  (7.01 to 7.42) |
| 176 | Guinea | 4.03 | 3.97 | 4.1 | 4.03  (3.97 to 4.1) |
| 177 | Guinea-Bissau | 5.01 | 4.85 | 5.17 | 5.01  (4.85 to 5.17) |
| 178 | Liberia | 4.56 | 4.2 | 4.92 | 4.56  (4.2 to 4.92) |
| 179 | Mali | 1.94 | 1.82 | 2.06 | 1.94  (1.82 to 2.06) |
| 180 | Mauritania | 4.66 | 4.53 | 4.8 | 4.66  (4.53 to 4.8) |
| 181 | Niger | 4.34 | 4.18 | 4.5 | 4.34  (4.18 to 4.5) |
| 182 | Nigeria | 4.4 | 4.22 | 4.58 | 4.4  (4.22 to 4.58) |
| 183 | Sao Tome and Principe | 6.06 | 5.87 | 6.26 | 6.06  (5.87 to 6.26) |
| 184 | Senegal | 5.01 | 4.81 | 5.21 | 5.01  (4.81 to 5.21) |
| 185 | Sierra Leone | 5.28 | 5.06 | 5.51 | 5.28  (5.06 to 5.51) |
| 186 | Togo | 5.61 | 5.46 | 5.76 | 5.61  (5.46 to 5.76) |
| 187 | American Samoa | 1.28 | 1.03 | 1.53 | 1.28  (1.03 to 1.53) |
| 188 | Bermuda | 0.35 | 0.15 | 0.56 | 0.35  (0.15 to 0.56) |
| 189 | Cook Islands | 1.66 | 1.43 | 1.88 | 1.66  (1.43 to 1.88) |
| 190 | Greenland | -0.23 | -0.38 | -0.07 | -0.23  (-0.38 to -0.07) |
| 191 | Guam | 0.97 | 0.72 | 1.22 | 0.97  (0.72 to 1.22) |
| 192 | Monaco | 1.14 | 1.13 | 1.16 | 1.14  (1.13 to 1.16) |
| 193 | Nauru | 0.68 | 0.46 | 0.91 | 0.68  (0.46 to 0.91) |
| 194 | Niue | 1.7 | 1.62 | 1.77 | 1.7  (1.62 to 1.77) |
| 195 | Northern Mariana Islands | -0.44 | -0.69 | -0.19 | -0.44  (-0.69 to -0.19) |
| 196 | Palau | 0.85 | 0.8 | 0.91 | 0.85  (0.8 to 0.91) |
| 197 | Puerto Rico | 0.59 | 0.41 | 0.78 | 0.59  (0.41 to 0.78) |
| 198 | Saint Kitts and Nevis | 1.25 | 1.03 | 1.47 | 1.25  (1.03 to 1.47) |
| 199 | San Marino | 0.22 | -0.18 | 0.63 | 0.22  (-0.18 to 0.63) |
| 200 | Tokelau | 1.79 | 1.75 | 1.84 | 1.79  (1.75 to 1.84) |
| 201 | Tuvalu | 2.02 | 1.91 | 2.12 | 2.02  (1.91 to 2.12) |
| 202 | United States Virgin Islands | 0.48 | 0.28 | 0.69 | 0.48  (0.28 to 0.69) |
| 203 | South Sudan | 1.29 | 1.15 | 1.44 | 1.29  (1.15 to 1.44) |
| 204 | Sudan | 2.83 | 2.68 | 2.99 | 2.83  (2.68 to 2.99) |
